# The 18-year risk of cancer, angioedema, insomnia, depression, and erectile dysfunction in association with antihypertensive drugs: post-trial analyses from ALLHAT–Medicare linked data

**DOI:** 10.3389/fcvm.2023.1272385

**Published:** 2023-11-17

**Authors:** Xianglin L. Du, Journey Martinez, Jose-Miguel Yamal, Lara M. Simpson, Barry R. Davis

**Affiliations:** ^1^ Department of Epidemiology, Human Genetics and Environmental Sciences, School of Public Health, The University of Texas Health Science Center at Houston, Houston, TX, United States; ^2^ Department of Biostatistics and Data Science, Coordinating Center for Clinical Trials, School of Public Health, The University of Texas Health Science Center at Houston, Houston, TX, United States

**Keywords:** cancer, angioedema, antihypertensive drugs, ALLHAT, Medicare claims data

## Abstract

**Purpose:**

This study aimed to determine the 18-year risk of cancer, angioedema, insomnia, depression, and erectile dysfunction in association with antihypertensive drug use.

**Methods:**

This is a post-trial passive follow-up study of Antihypertensive and Lipid-Lowering Treatment to Prevent Heart Attack Trial (ALLHAT) participants between 1994 and 1998 that was conducted by linking their follow-up data with Medicare claims data until 2017 of subjects who were free of outcomes at baseline on 1 January 1999. The main outcomes were the occurrence of cancer (among *n* = 17,332), angioedema (among *n* = 17,340), insomnia (among *n* = 17,340), depression (among *n* = 17,330), and erectile dysfunction (among *n* = 7,444 men) over 18 years of follow-up.

**Results:**

The 18-year cumulative incidence rate of cancer other than non-melanoma skin cancer from Medicare inpatient claims was 23.9% for chlorthalidone, 23.4% for amlodipine, and 25.3% for lisinopril. There were no statistically significant differences in the 18-year risk of cancer, depression, and erectile dysfunction among the three drugs based on the adjusted hazard ratios. The adjusted 18-year risk of angioedema was elevated in those receiving lisinopril than in those receiving amlodipine (hazard ratio: 1.63, 95% CI: 1.14–2.33) or in those receiving chlorthalidone (1.33, 1.00–1.79), whereas the adjusted 18-year risk of insomnia was statistically significantly decreased in those receiving lisinopril than in those receiving amlodipine (0.90, 0.81–1.00).

**Conclusions:**

The 18-year risk of angioedema was significantly higher in patients receiving lisinopril than in those receiving amlodipine or chlorthalidone; the risk of insomnia was significantly lower in patients receiving lisinopril than in those receiving amlodipine; and the risk of cancer, depression, and erectile dysfunction (in men) was not statistically significantly different among the three drug groups.

## Introduction

The Antihypertensive and Lipid-Lowering Treatment to Prevent Heart Attack Trial (ALLHAT), which was a multicenter, randomized, double-blind, active-controlled trial in 623 North American centers and was completed with in-trial follow-up in 2002, produced some major findings (1, 2) that have translated into clinical practice guidance (3–8). For example, this study found that amlodipine (calcium channel blocker), lisinopril [angiotensin-converting enzyme (ACE) inhibitor], and doxazosin were no superior to chlorthalidone (thiazide diuretic) in preventing most types of cardiovascular disease (CVD) and chlorthalidone was superior in preventing heart failure (1, 2). This study also found that the secondary outcomes, such as cancer and hospitalized gastrointestinal (GI) bleeding, had no significantly different 6-year incidence rates and adjusted hazard ratios between these three treatment groups (9). The cumulative incidence rates of non-hospitalized GI bleeding were also similar across the three groups (12.0%, 12.2%, and 12.0% for amlodipine, lisinopril, and chlorthalidone, respectively) (10). According to previous studies, the use of antihypertensive drugs might be associated with an increased risk of angioedema, insomnia, depression, and erectile dysfunction, in addition to cancer, GI bleeding, and dementia (1, 2, 9–17). Some of these side effects were reported from clinical observations, cross-sectional surveys, or retrospective cohort studies, which are extremely vulnerable to selection bias and confounding. The causations have not been well established through the confirmation of long-term follow-up studies of well-conducted randomized clinical trials or cohort studies. Now, data of ALLHAT participants were linked with their Medicare data until December 2017, which enabled us to examine the long-term outcomes associated with different antihypertensive drugs used among the trial participants. Therefore, this study aimed to determine the risk of cancer, angioedema, insomnia, depression, and erectile dysfunction in association with three antihypertensive drugs and other factors among ALLHAT participants with up to 23-year follow-up from 1994 to 2017 and 18-year Medicare-linked data from 1999 to 2017.

## Methods

### Study design, population, and data sources

**Figure 1:**
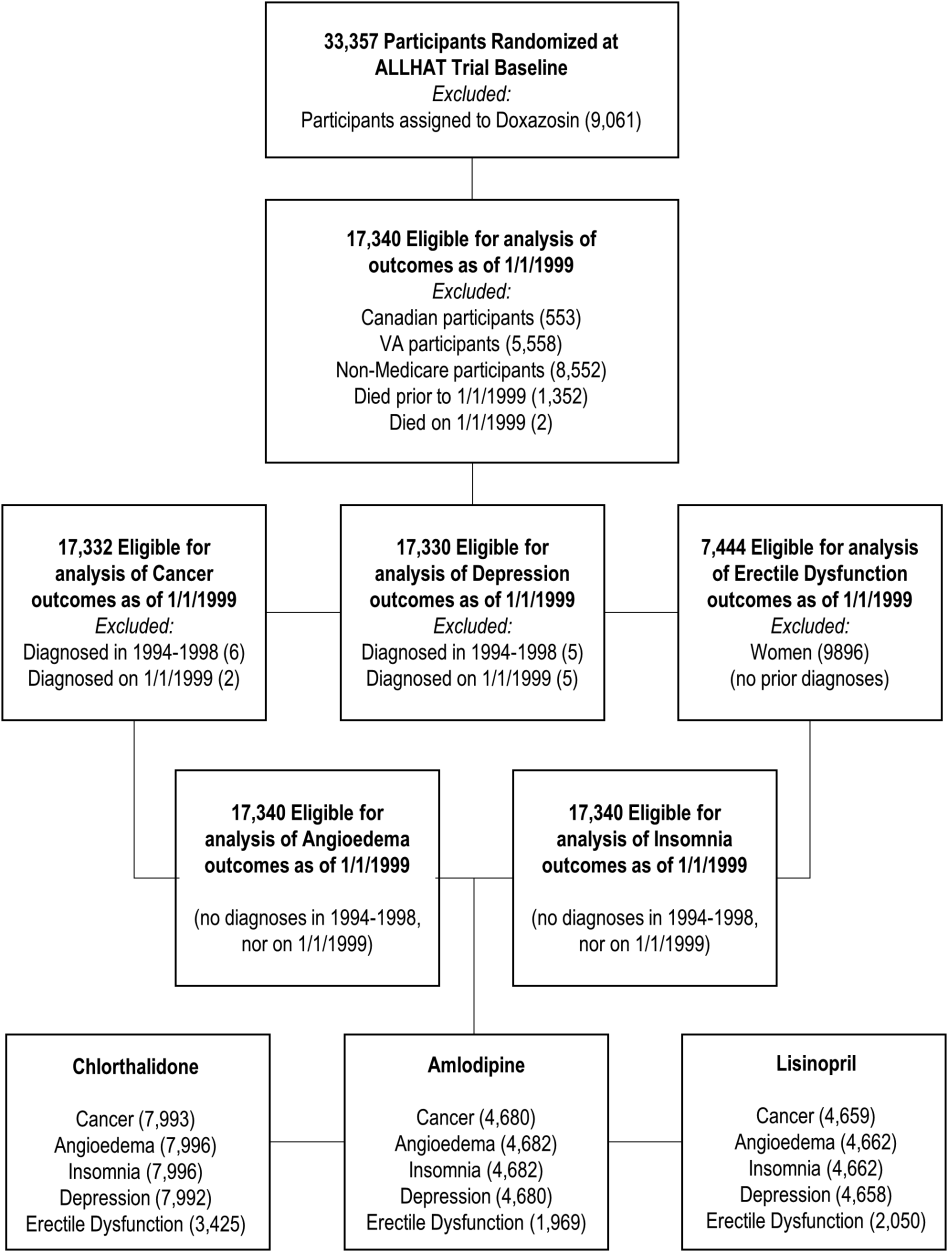
Flowchart for inclusion and exclusion based on Medicare inpatient data.

The detailed methods of the ALLHAT have been reported previously (1, 2). In brief, the ALLHAT was a multicenter, randomized, double-blind, active-controlled trial conducted on 42,418 participants aged ≥55 years with hypertension and at least one other coronary heart disease (CHD) risk factor in 623 North American centers. Those patients who were eligible and agreed to participate were randomly assigned to four treatment groups: an ACE inhibitor (lisinopril) (*n* = 9,054), a calcium channel blocker (CCB, amlodipine) (*n* = 9,048), an *α*-blocker (doxazosin) (*n* = 9,061), or a thiazide-type diuretic (chlorthalidone) (*n* = 15,255). This study did not include patients on doxazosin because this group was terminated earlier. Data of ALLHAT participants aged ≥65 years (because the nationwide Medicare health insurance program covers those aged 65 years or older) were linked with their Medicare inpatient, outpatient, and physician carrier claims data available from 1 January 1999 to 31 December 2017. Of the 33,357 participants (*n* = 9,054 for lisinopril, *n* = 9,048 for amlodipine, and *n* = 15,255 for chlorthalidone), the study excluded the following subjects: Canadian participants (553), VA participants (5,558), non-Medicare participants (8,552), and those who died on or prior to 1 January 1999 (1,354), leaving 17,340 participants for this study. Because we examined five different study outcomes and considered the baseline as 1 January 1999, we excluded a varying number of subjects based on whether Medicare inpatient claims data from 1 January 1994 to 31 December 1998 had each respective outcome. These individuals were considered to have the condition at baseline and, therefore, were excluded, leaving between 17,330 and 17,340 total subjects and 7,444 men in the final analysis (see Figure 1 for the flowchart, Table 1, and Supplementary Tables S1A–D).

**Table 1:** Baseline characteristics of the cancer outcome cohort: full sample and by the randomized group.

Participants, *N* (%) (unless otherwise indicated)					
	Full sample	Chlorthalidone	Amlodipine	Lisinopril	*P*-value^a,b^
Eligible for further follow-up as of 1 January 1999	17,332	7,993	4,680	4,659	
Hospitalized cancer diagnosis (1999–2017)	2,857 (16.5)	1,288 (16.1)	760 (16.2)	809 (17.4)	0.164
Non-hospitalized cancer diagnosis (1999–2017)^c^	0	0	0	0	
Hospitalized or non-hospitalized cancer diagnosis (1999–2017)	2,857 (16.5)	1,288 (16.1)	760 (16.2)	809 (17.4)	0.164
Age, mean (SD) (years)	73.43 (6.34)	73.37 (6.30)	73.48 (6.39)	73.50 (6.36)	0.485
Age group (as of 1 January 1999) (years)					
Age <70	6,035 (34.8)	2,822 (35.3)	1,611 (34.4)	1,602 (34.4)	0.543
Age 70–79	8,489 (49.0)	3,914 (49.0)	2,290 (48.9)	2,285 (49.0)	
Age 80+	2,808 (16.2)	1,257 (15.7)	779 (16.6)	772 (16.6)	
Gender					
Male	7,439 (42.9)	3,422 (42.8)	1,968 (42.1)	2,049 (44.0)	0.164
Female	9,893 (57.1)	4,571 (57.2)	2,712 (57.9)	2,610 (56.0)	
Race/ethnicity					
Black	11,312 (65.3)	5,225 (65.4)	3,046 (65.1)	3,041 (65.3)	0.949
Non-Black	6,020 (34.7)	2,768 (34.6)	1,634 (34.9)	1,618 (34.7)	
Hispanic/Latino ethnicity					
Hispanic	3,631 (21.1)	1,668 (21.0)	959 (20.6)	1,004 (21.7)	0.436
Non-Hispanic	13,612 (78.9)	6,288 (79.0)	3,695 (79.4)	3,629 (78.3)	
Education, mean (SD) (years)	10.57 (4.21)	10.60 (4.20)	10.54 (4.13)	10.56 (4.33)	0.747
Education level					
High school or less	11,875 (74.1)	5,455 (73.7)	3,226 (74.5)	3,194 (74.3)	0.549
More than high school	4,151 (25.9)	1,947 (26.3)	1,102 (25.5)	1,102 (25.7)	
Treatment with antihypertensive drugs prior to trial baseline					
Treated	15,709 (90.6)	7,213 (90.2)	4,257 (91.0)	4,239 (91.0)	0.257
Untreated	1,623 (9.4)	780 (9.8)	423 (9.0)	420 (9.0)	
Aspirin use (as of 1 January 1999)	6,406 (37.3)	2,961 (37.4)	1,697 (36.6)	1,748 (37.9)	0.421
Women taking estrogen at trial baseline	1,444 (14.9)	695 (15.5)	385 (14.4)	364 (14.2)	0.261
HDL cholesterol (as of 1 January 1999), mean (SD) (mg/dl)	48.16 (14.84)	48.09 (14.84)	48.58 (15.09)	47.85 (14.59)	0.059
HDL <35 mg/dl (as of 1 January 1999)	2,784 (16.1)	1,290 (16.1)	713 (15.2)	781 (16.8)	0.128
Cigarette smoking at trial baseline					
Never smoker	2,842 (16.4)	1,327 (16.6)	789 (16.9)	726 (15.6)	0.273
Current smoker	6,840 (39.5)	3,166 (39.6)	1,800 (38.5)	1,874 (40.2)	
Former smoker	7,649 (44.1)	3,500 (43.8)	2,091 (44.7)	2,058 (44.2)	
Diabetes classification (as of 1 January 1999)					
Diabetic	7,091 (44.1)	3,288 (44.3)	1,917 (44.3)	1,886 (43.7)	0.779
Non-diabetic	8,976 (55.9)	4,134 (55.7)	2,410 (55.7)	2,432 (56.3)	
History of CHD (as of 1 January 1999)	4,845 (28.0)	2,275 (28.5)	1,260 (26.9)	1,310 (28.1)	0.169
ASCVD at trial baseline	9,479 (54.7)	4,418 (55.3)	2,490 (53.2)	2,571 (55.2)	0.057
History of MI or stroke (as of 1 January 1999)	4,702 (27.1)	2,166 (27.1)	1,275 (27.2)	1,261 (27.1)	0.978
History of CABG (as of 1 January 1999)	2,676 (15.4)	1,244 (15.6)	678 (14.5)	754 (16.2)	0.070
Other ASCVDs at trial baseline	4,565 (26.3)	2,104 (26.3)	1,215 (26.0)	1,246 (26.7)	0.691
Major ST segment depression (as of 1 January 1999)	1,389 (8.0)	667 (8.4)	337 (7.2)	385 (8.3)	0.055
Hard LVH by the Minnesota code (as of 1 January 1999)	675 (4.4)	314 (4.4)	182 (4.4)	179 (4.3)	0.983
LLT participants	4,217 (24.3)	1,978 (24.7)	1,150 (24.6)	1,089 (23.4)	0.200
BMI, mean (SD) (kg/m^2^), at trial baseline	29.38 (5.98)	29.33 (6.02)	29.42 (5.97)	29.44 (5.92)	0.567
Obesity (BMI ≥ 30 kg/m^2^) at trial baseline	6,875 (39.7)	3,153 (39.4)	1,858 (39.7)	1,864 (40.0)	0.822
Latest BP reading prior to 1 January 1999 (mmHg)					
Systolic BP, mean (SD)	140.09 (16.59)	139.22 (16.24)	140.12 (15.71)	141.54 (17.88)	**<0.001**
Diastolic BP, mean (SD)	78.90 (10.04)	79.01 (9.91)	78.33 (9.87)	79.30 (10.42)	**<0.001**
Blood pressure change from baseline to the latest BP reading prior to 1 January 1999 (mmHg)					
Systolic BP, mean (SD)	−7.22 (18.40)	−8.10 (17.96)	−6.92 (18.25)	−6.03 (19.19)	**<0.001**
Diastolic BP, mean (SD)	−4.43 (10.57)	−4.50 (10.48)	−4.82 (10.49)	−3.90 (10.79)	**<0.001**

ASCVD, atherosclerotic cardiovascular disease; CABG, coronary artery bypass graft; LVH, left ventricular hypertrophy; LLT, lipid-lowering trial; BP, blood pressure; *N*, number of participants; SD, standard deviation.

^a^ *P*-values represent the significance level of the chi-squared test of independence between randomized groups for binary and categorical variables or the one-way ANOVA between randomized groups for continuous variables.

^b^ Statistically significant *p*-values (<0.05) are shown in bold.

^c^ Cancer diagnoses excluded non-melanoma skin cancer and were considered from inpatient data only.

### Study variables

#### Main exposures

The main exposures were three antihypertensive drugs (lisinopril, amlodipine, and chlorthalidone) that were prescribed to participants through initial trial randomization and continued until 1 January 1999 as the baseline for this passive follow-up because complete Medicare data (inpatient, outpatient, and physician carrier data files) were available from this time to 31 December 2017. After the ALLHAT ended in March 2002, participants were not followed for their post-study antihypertensive treatment.

#### Main outcomes

The main outcomes were the occurrence of cancer (any cancer other than non-melanoma skin cancer), angioedema, insomnia, depression, and erectile dysfunction (in men). These outcomes were defined if there were ICD-9 or ICD-10 diagnosis codes (Supplementary Tables S2A–C) in Medicare claims data (inpatient, outpatient, and physician carrier claims) that occurred on one or the first of more occasions after the baseline (1 January 1999) to the date of last follow-up (31 December 2017). For sensitivity analyses, the findings were presented when the outcomes were defined from any diagnosis codes and primary diagnosis codes only (i.e., the first diagnosis code out of 12 diagnosis codes for carrier data and 25 diagnosis codes for inpatient and outpatient data) that occurred on at least two separate occasions 30 days apart in Medicare claims data from 1999 to 2017 (Supplementary Tables S3A–D and S4).

#### Covariates

ALLHAT baseline (at the time of randomization) demographic and clinical data including age, gender, race/ethnicity, education, prior receipt of antihypertensive drug therapy, estrogen use (for women), smoking, history of atherosclerotic cardiovascular disease, other atherosclerotic cardiovascular disease, and obesity (body mass index, BMI ≥30 kg/m^2^), were incorporated into analyses. Wherever possible, covariate data were gathered from extension trial follow-up visits that were most proximal to but did not extend beyond 1 January 1999. Extension trial data up to 1 January 1999 were available for aspirin use, high-density lipoprotein (HDL) cholesterol level <35 mg/dl, diabetes, history of coronary heart disease, coronary artery bypass graft, major ST segment depression, left ventricular hypertrophy by the Minnesota code, and systolic and diastolic blood pressure. In the absence of targeted follow-up during the extension phase on the status of myocardial infarction (MI) or stroke, data on the history of MI or stroke were supplemented with relevant diagnosis codes garnered from Medicare inpatient claims data from 1 January 1994 to 31 December 1998.

### Statistical analysis

Baseline characteristics among the study comparison groups were compared using chi-squared statistics for categorical variables and one-way analysis of variance (ANOVA) for continuous variables. The 6-year and 18-year cumulative incidence rates of cancer (any cancer other than non-melanoma skin cancer), angioedema, insomnia, depression, and erectile dysfunction (in men) were calculated from the baseline on 1 January 1999 to 1 January 2005 for the 6-year rate and to the date of last follow-up (31 December 2017) for the 18-year rate using the Kaplan–Meier method. Subjects who died or were lost to follow-up were censored. The 6-year incidence rates and hazard ratios of outcomes from Medicare claims data were presented to be compared with the findings of original ALLHAT reports that used the 6-year rates and criteria (1).

The population at risk were those who were free of the respective outcomes (cancer, angioedema, insomnia, depression, or erectile dysfunction) at baseline in 1999, as determined from the data available in Medicare inpatient, outpatient, and carrier data sets. In addition, Cox regression models were used to perform time-to-event analyses to determine the risk of developing the above outcomes by the three study drugs while adjusting for all measured confounding factors listed in the tables. The proportionality assumption for multivariable models was assessed by the Schoenfeld residuals test and by visually inspecting whether the log–log Kaplan–Meier curves were parallel and did not intersect. There was no adjustment for multiple comparisons. A *p* < 0.05 was considered statistically significant. Analyses were conducted using SAS version 9.4 and R version 4.0.2 (R Foundation for Statistical Computing).

## Results

Comparisons of baseline characteristics among study participants taking three antihypertensive drugs (chlorthalidone, amlodipine, and lisinopril) on 1 January 1999 were presented for cancer (Table 1) and other outcomes (Supplementary Table S1A–D). Of those subjects who were free of outcomes (cancer, angioedema, insomnia, depression, and erectile dysfunction), the baseline characteristics such as age, gender, race/ethnicity, history of vascular diseases, diabetes, and obesity were similar without statistically significant differences among the three drug groups.

Table 2 presents the cumulative incidence of outcomes (cancer, angioedema, insomnia, depression, and erectile dysfunction) that occurred over the next 6 and 18 years of follow-up from 1 January 1999 to 31 December 2017 and were identified from Medicare inpatient hospitalization data only. For example, the 6-year cumulative incidence rate of cancer other than non-melanoma skin cancer was 9.5% for chlorthalidone, 9.8% for amlodipine, and 10.5% for lisinopril, which was identical to what was reported (9.7%, 10.0%, and 9.9%, respectively, for the above three drug groups) in an ALLHAT main report (1). The 18-year cumulative incidence rate of cancer other than non-melanoma skin cancer from Medicare inpatient hospitalization data was 23.9% for chlorthalidone, 23.4% for amlodipine, and 25.3% for lisinopril (Table 2). After adjusting for socio-demographics, comorbidities, and other potential confounding factors in the time-to-event Cox models (Table 3), the 6-year risk of cancer was statistically significantly higher for lisinopril vs. chlorthalidone (adjusted hazard ratio: 1.19, 95% CI: 1.03–1.36) but was not statistically significantly different among two other drug groups for chlorthalidone vs. amlodipine (1.03, 0.90–1.19) and for lisinopril vs. amlodipine (1.15, 0.98–1.34). The 18-year risk of cancer was not statistically significantly different among the three drug groups [0.98 (95% CI: 0.89–1.09) for chlorthalidone vs. amlodipine, 1.06 (0.95–1.19) for lisinopril vs. amlodipine, and 1.05 (0.95–1.16) for lisinopril vs. chlorthalidone, all *p*s > 0.05)]. Including cases with outcomes that were identified from Medicare inpatient, outpatient, and physician carrier claims data, the cumulative incidence rate of cancer was much higher. For example, the 6-year cumulative incidence rate of cancer other than non-melanoma skin cancer from Medicare inpatient, outpatient, and physician carrier claims data was 38.5% for chlorthalidone, 38.5% for amlodipine, and 39.3% for lisinopril, whereas the 18-year cumulative incidence rate of cancer other than non-melanoma skin cancer from Medicare inpatient, outpatient, and physician carrier claims data was 61.1% for chlorthalidone, 60.4% for amlodipine, and 62.7% for lisinopril (Supplementary Table S3A). The adjusted hazard ratios of cancer were not statistically significantly different among these three drug groups (Supplementary Table S4).

**Table 2:** Cumulative incidence (%) of five outcomes from any diagnosis in Medicare inpatient data on ≥1 time by the three study drugs and other factors (1999–2017).

Demographic	Cancer			Angioedema			Insomnia			Depression			Erectile dysfunction (in men)		
	Events/total (*n*/*N*)	6-year % (SE)	18-year %^a^ (SE)	Events /total (*n*/*N*)	6-year % (SE)	18-year %^a^ (SE)	Events/total (*n*/*N*)	6-year % (SE)	18-year %^a^ (SE)	Events/total (*n*/*N*)	6-year % (SE)	18-year %^a^ (SE)	Events/total (*n*/*N*)	6-year % (SE)	18-year %^a^ (SE)
All patients	2,857/17,332	9.8 (0.2)	24.2 (0.4)	296/17,340	1 (0.1)	2.8 (0.2)	3,359/17,340	12.3 (0.3)	29.9 (0.5)	5,456/17,330	20.8 (0.3)	45.4 (0.5)	1,079/7,444	14.3 (0.4)	19.2 (0.6)
Randomized group															
Chlorthalidone	1,288/7,993	9.5 (0.3)	23.9 (0.6)	126/7,996	0.9 (0.1)	2.7 (0.3)	1,547/7,996	12.3 (0.4)	30.0 (0.7)	2,531/7,992	20.9 (0.5)	45.4 (0.8)	518/3,425	14.5 (0.6)	20.6 (0.9)
Amlodipine	760/4,680	9.8 (0.5)	23.4 (0.8)	68/4,682	0.9 (0.2)	2.3 (0.3)	934/4,682	12.9 (0.5)	30.6 (1.0)	1,487/4,680	20.7 (0.6)	45.8 (1.0)	273/1,969	14.1 (0.8)	17.7 (1.1)
Lisinopril	809/4,659	10.5 (0.5)	25.3 (0.9)	102/4,662	1.3 (0.2)	3.6 (0.4)	878/4,662	11.7 (0.5)	29.2 (0.9)	1,438/4,658	20.6 (0.6)	45.1 (1.0)	288/2,050	14.2 (0.8)	18.2 (1.1)
Age group (as of 1 January 1999) (years)															
Age <70	988/6,035	7.8 (0.4)	21.6 (0.6)	116/6,037	0.9 (0.1)	2.8 (0.3)	1,369/6,037	12.0 (0.4)	30.8 (0.7)	1,992/6,035	18.8 (0.5)	42.8 (0.8)	542/2,749	18.1 (0.8)	23.8 (0.9)
Age 70–79	1,469/8,489	10.4 (0.3)	25.2 (0.6)	147/8,494	1.1 (0.1)	2.8 (0.3)	1,622/8,494	12.4 (0.4)	29.5 (0.7)	2,674/8,488	20.6 (0.5)	46.4 (0.8)	475/3,669	12.8 (0.6)	17.0 (0.8)
Age 80+	400/2,808	13.3 (0.7)	26.4 (1.6)	33/2,809	1.0 (0.2)	2.3 (0.5)	368/2,809	12.6 (0.7)	25.4 (1.6)	790/2,807	26.7 (1.0)	48.7 (1.8)	62/1,026	8.2 (1.0)	8.2 (1.0)
Gender															
Male	1,462/7,439	12.1 (0.4)	29.9 (0.7)	95/7,444	0.9 (0.1)	2.1 (0.2)	1,354/7,444	12.0 (0.4)	30.2 (0.8)	1,900/7,443	16.3 (0.5)	40.4 (0.8)	—	—	—
Female	1,395/9,893	8.2 (0.3)	20.2 (0.5)	201/9,896	1.1 (0.1)	3.3 (0.2)	2,005/9,896	12.5 (0.4)	29.8 (0.6)	3,556/9,887	24.0 (0.5)	49.0 (0.7)	—	—	—
Race/ethnicity															
Black	1,063/6,020	11.0 (0.4)	25.9 (0.8)	173/6,024	1.5 (0.2)	4.9 (0.4)	927/6,024	9.3 (0.4)	24.5 (0.8)	1,679/6,019	17.9 (0.5)	41.8 (0.9)	335/2,061	16.8 (0.9)	22.4 (1.2)
Non-Black	1,794/11,312	9.2 (0.3)	23.2 (0.5)	123/11,316	0.7 (0.1)	1.7 (0.2)	2,432/11,316	13.9 (0.3)	32.7 (0.6)	3,777/11,311	22.3 (0.4)	47.3 (0.6)	744/5,383	13.4 (0.5)	18.0 (0.7)
Hispanic/Latino ethnicity															
Hispanic	359/3,631	7.1 (0.4)	13.0 (0.7)	36/3,631	0.6 (0.1)	1.5 (0.3)	591/3,631	11.6 (0.6)	21.8 (0.9)	887/3,630	18.5 (0.7)	31.3 (0.9)	176/1,448	12.4 (0.9)	14.8 (1.1)
Non-Hispanic	2,478/13,612	10.6 (0.3)	27.4 (0.5)	260/13,620	1.1 (0.1)	3.2 (0.2)	2,749/13,620	12.5 (0.3)	32.6 (0.6)	4,541/13,611	21.5 (0.4)	49.8 (0.6)	896/5,948	14.8 (0.5)	20.4 (0.7)
Education level															
<High school	1,943/11,875	10.3 (0.3)	24.1 (0.5)	200/11,880	1.0 (0.1)	2.8 (0.2)	2,202/11,880	11.9 (0.3)	29.0 (0.6)	3,681/11,873	21.2 (0.4)	45.1 (0.6)	622/4,631	13.6 (0.5)	18.2 (0.7)
≥High school	724/4,151	8.7 (0.5)	24.7 (0.9)	74/4,154	0.8 (0.1)	2.8 (0.3)	940/4,154	13.2 (0.6)	33.9 (1.0)	1,412/4,153	19.8 (0.6)	47.7 (1.0)	375/2,286	15.2 (0.8)	20.7 (1.0)
Treatment with antihypertensive drugs prior to trial baseline															
Treated	2,608/15,709	10.0 (0.3)	24.3 (0.5)	280/15,717	1.0 (0.1)	3.0 (0.2)	3,089/15,717	12.6 (0.3)	30.5 (0.5)	4,976/15,707	21.0 (0.3)	45.7 (0.5)	972/6,629	14.6 (0.5)	19.4 (0.6)
Untreated	249/1,623	8.3 (0.7)	22.4 (1.4)	16/1,623	0.8 (0.2)	1.4 (0.4)	270/1,623	9.6 (0.8)	24.9 (1.4)	480/1,623	18.6 (1.0)	42.8 (1.7)	107/815	12.2 (1.2)	17.1 (1.6)
Aspirin use (as of 1 January 1999)															
Yes	1,080/6,406	10.0 (0.4)	25.3 (0.7)	101/6,410	1.1 (0.1)	2.5 (0.3)	1,302/6,410	12.6 (0.4)	32.7 (0.9)	2,049/6,406	19.9 (0.5)	48.1 (0.9)	504/3,356	14.6 (0.7)	19.9 (0.9)
No	1,753/10,760	9.8 (0.3)	23.5 (0.5)	193/10,764	1.0 (0.1)	2.9 (0.2)	2,036/10,764	12.1 (0.3)	28.5 (0.6)	3,368/10,758	21.4 (0.4)	44.1 (0.6)	568/4,013	14.2 (0.6)	18.7 (0.8)
Women taking estrogen at trial baseline															
Yes	202/1,444	6.0 (0.6)	18.5 (1.2)	35/1,444	1.0 (0.3)	3.5 (0.6)	419/1,444	17.4 (1.0)	38.4 (1.6)	652/1,443	29.7 (1.2)	55.7 (1.6)	—	—	—
No	1,174/8,271	8.6 (0.3)	20.6 (0.6)	161/8,274	1.1 (0.1)	3.2 (0.3)	1,549/8,274	11.6 (0.4)	28.0 (0.7)	2,847/8,266	23.0 (0.5)	47.8 (0.7)	—	—	—
HDL cholesterol <35 mg/dl (as of 1 January 1999)															
Yes	545/2,784	12.0 (0.7)	30.1 (1.2)	35/2,784	0.8 (0.2)	2.0 (0.4)	548/2,784	12.9 (0.7)	33.3 (1.4)	837/2,784	19.8 (0.8)	46.0 (1.4)	286/1,909	15.3 (0.9)	18.8 (1.1)
No	2,312/14,548	9.4 (0.3)	23.1 (0.5)	261/14,556	1.0 (0.1)	2.9 (0.2)	2,811/14,556	12.2 (0.3)	29.4 (0.5)	4,619/14,546	21.0 (0.4)	45.4 (0.6)	793/5,535	14.0 (0.5)	19.3 (0.7)
Cigarette smoking at trial baseline															
Never smoker	1,031/7,649	7.7 (0.3)	19.4 (0.6)	138/7,649	1.0 (0.1)	2.9 (0.3)	1,498/7,649	11.9 (0.4)	28.5 (0.7)	2,510/7,642	20.9 (0.5)	44.9 (0.7)	284/1,895	14.4 (0.9)	19.2 (1.1)
Current smoker	627/2,842	14.3 (0.7)	33.2 (1.2)	46/2,845	1.2 (0.2)	2.7 (0.5)	468/2,845	11.3 (0.7)	28.8 (1.4)	822/2,843	21.4 (0.8)	44.2 (1.4)	179/1,389	13.3 (1.0)	17.9 (1.3)
Former smoker	1,199/6,840	10.4 (0.4)	26.2 (0.7)	112/6,845	0.9 (0.1)	2.7 (0.3)	1,393/6,845	13.1 (0.4)	32.3 (0.8)	2,123/6,844	20.5 (0.5)	46.6 (0.9)	615/4,159	14.6 (0.6)	19.5 (0.8)
Diabetes classification (as of 1 January 1999)															
Diabetic	1,110/7,091	9.9 (0.4)	24.6 (0.7)	122/7,091	1.0 (0.1)	3.1 (0.3)	1,292/7,091	12.2 (0.4)	30.0 (0.8)	2,242/7,086	22.1 (0.5)	48.2 (0.9)	386/2,889	13.6 (0.7)	18.4 (1.0)
Non-diabetic	1,528/8,976	9.6 (0.3)	23.8 (0.6)	152/8,981	1.0 (0.1)	2.6 (0.2)	1,829/8,981	12.4 (0.4)	30.0 (0.7)	2,833/8,976	19.8 (0.4)	43.9 (0.7)	626/4,031	14.9 (0.6)	19.9 (0.8)
History of CHD (as of 1 January 1999)															
Yes	779/4,845	10.2 (0.5)	24.3 (0.8)	68/4,848	1.0 (0.2)	2.3 (0.3)	980/4,848	13.3 (0.5)	34.0 (1.1)	1,542/4,844	22.5 (0.6)	47.7 (1.0)	346/2,593	13.4 (0.7)	18.6 (1.1)
No	2,078/12,487	9.7 (0.3)	24.0 (0.5)	228/12,492	1.0 (0.1)	3.0 (0.2)	2,379/12,492	11.9 (0.3)	28.6 (0.6)	3,914/12,486	20.2 (0.4)	44.6 (0.6)	733/4,851	14.8 (0.5)	19.5 (0.7)
ASCVD at trial baseline															
Yes	1,529/9,479	10.0 (0.3)	24.4 (0.6)	174/9,482	1.1 (0.1)	3.1 (0.3)	1,875/9,482	13.1 (0.4)	31.5 (0.7)	3,019/9,478	21.9 (0.5)	46.8 (0.7)	602/4,351	14.1 (0.6)	18.8 (0.8)
No	1,328/7,853	9.6 (0.4)	23.9 (0.6)	122/7,858	0.9 (0.1)	2.4 (0.2)	1,484/7,858	11.4 (0.4)	28.2 (0.7)	2,437/7,852	19.5 (0.5)	43.9 (0.8)	477/3,093	14.7 (0.7)	19.7 (0.9)
History of MI or stroke (as of 1 January 1999)															
Yes	761/4,702	11.2 (0.5)	26.4 (1.0)	78/4,705	1.1 (0.2)	2.9 (0.4)	867/4,705	13.1 (0.5)	33.0 (1.1)	1,527/4,700	24.6 (0.7)	50.5 (1.1)	279/2,388	11.9 (0.7)	17.4 (1.2)
No	2,096/12,630	9.4 (0.3)	23.4 (0.5)	218/12,635	1.0 (0.1)	2.7 (0.2)	2,492/12,635	12.0 (0.3)	29.1 (0.6)	3,929/12,630	19.5 (0.4)	43.8 (0.6)	800/5,056	15.4 (0.5)	20.0 (0.7)
History of CABG (as of 1 January 1999)															
Yes	441/2,676	9.9 (0.6)	26.4 (1.2)	38/2,677	1.2 (0.2)	2.3 (0.5)	570/2,677	14.0 (0.7)	37.0 (1.6)	859/2,676	22.3 (0.9)	49.9 (1.5)	249/1,721	14.3 (0.9)	20.0 (1.3)
No	2,416/14,656	9.8 (0.3)	23.8 (0.5)	258/14,663	1.0 (0.1)	2.9 (0.2)	2,789/14,663	12.0 (0.3)	28.9 (0.5)	4,597/14,654	20.5 (0.4)	44.8 (0.6)	830/5,723	14.3 (0.5)	19.0 (0.7)
Other ASCVD at trial baseline															
Yes	723/4,565	9.7 (0.5)	23.8 (0.9)	81/4,567	1.0 (0.2)	3.1 (0.4)	916/4,567	13.5 (0.5)	31.6 (1.0)	1,469/4,565	22.0 (0.7)	47.0 (1.0)	252/1,934	13.3 (0.8)	17.3 (1.1)
No	2,134/12,767	9.9 (0.3)	24.3 (0.5)	215/12,773	1.0 (0.1)	2.7 (0.2)	2,443/12,773	11.9 (0.3)	29.4 (0.6)	3,987/12,765	20.4 (0.4)	44.9 (0.6)	827/5,510	14.7 (0.5)	19.7 (0.7)
Major ST segment depression (as of 1 January 1999)															
Yes	231/1,389	10.3 (0.9)	26.2 (1.7)	29/1,389	1.3 (0.3)	3.9 (0.8)	262/1,389	12.6 (1.0)	30.3 (1.8)	401/1,389	19.4 (1.1)	43.8 (1.9)	98/554	18.2 (1.8)	24.4 (2.4)
No	2,615/15,890	9.8 (0.2)	24.0 (0.4)	267/15,898	1.0 (0.1)	2.7 (0.2)	3,088/15,898	12.3 (0.3)	29.9 (0.5)	5,041/15,888	20.9 (0.3)	45.6 (0.5)	979/6,872	14.0 (0.5)	18.8 (0.6)
LVH by the Minnesota code (as of 1 January 1999)															
Hard LVH	106/675	12.2 (1.4)	26.3 (2.6)	8/675	0.8 (0.4)	3.7 (1.5)	90/675	10.7 (1.4)	26.1 (3.0)	200/674	22.4 (1.8)	51.2 (3.3)	37/266	15.8 (2.5)	17.2 (2.8)
No/soft LVH	2,467/14,767	9.7 (0.3)	24.4 (0.5)	261/14,774	1.0 (0.1)	2.8 (0.2)	2,977/14,774	12.5 (0.3)	31.0 (0.5)	4,712/14,767	20.7 (0.4)	45.8 (0.6)	937/6,471	14.1 (0.5)	19.3 (0.6)
LLT participant															
Yes	645/4,217	9.1 (0.5)	21.5 (0.8)	69/4,219	0.9 (0.2)	2.7 (0.3)	800/4,219	11.9 (0.5)	28.6 (1.0)	1,299/4,218	19.6 (0.6)	42.9 (1.0)	247/1,757	14.2 (0.9)	18.0 (1.1)
No	2,212/13,115	10.1 (0.3)	25.1 (0.5)	227/13,121	1.0 (0.1)	2.8 (0.2)	2,559/13,121	12.4 (0.3)	30.4 (0.6)	4,157/13,112	21.2 (0.4)	46.3 (0.6)	832/5,687	14.4 (0.5)	19.5 (0.7)
Obesity (BMI ≥ 30 kg/m^2^) at trial baseline															
Yes	1,149/6,875	9.4 (0.4)	24.1 (0.7)	158/6,881	1.2 (0.1)	3.8 (0.3)	1,505/6,881	13.2 (0.4)	33.1 (0.8)	2,342/6,876	21.5 (0.5)	48.3 (0.8)	387/2,570	14.8 (0.7)	19.4 (1.0)
No	1,708/10,457	10.2 (0.3)	24.2 (0.6)	138/10,459	0.8 (0.1)	2.1 (0.2)	1,854/10,459	11.7 (0.3)	27.7 (0.6)	3,114/10,454	20.3 (0.4)	43.4 (0.7)	692/4,874	14.1 (0.5)	19.0 (0.7)

ASCVD, atherosclerotic cardiovascular disease; CABG, coronary artery bypass graft; LVH, left ventricular hypertrophy; LLT, lipid-lowering trial; *n*, number of patients experiencing the event; *N*, number of patients eligible to be studied for the event; SE, standard error.

Cancer diagnoses excluded non-melanoma skin cancer and included diagnoses from inpatient data only.

^a^ 18-year time to event includes events occurring up to, but not including, year 19 (e.g., throughout 2017).

**Table 3:** Adjusted^a^ HRs of having five outcomes from any diagnosis in Medicare inpatient data on ≥1 time by the three study drugs and other factors (1999–2017).

Demographic	Cancer				Angioedema				Insomnia				Depression				Erectile dysfunction (in men)			
	6-year		18-year		6-year		18-year		6-year		18-year		6-year		18-year		6-year		18-year	
	HR (95% CI)	*P*-value	HR (95% CI)	*P*-value	HR (95% CI)	*P*-value	HR (95% CI)	*P*-value	HR (95% CI)	*P*-value	HR (95% CI)	*P*-value	HR (95% CI)	*P*-value	HR (95% CI)	*P*-value	HR (95% CI)	*P*-value	HR (95% CI)	*P*-value
Randomized group																				
Chlorthalidone vs. amlodipine	1.03 (0.90–1.19)	0.642	0.98 (0.89–1.09)	0.757	0.82 (0.54–1.45)	0.632	0.82 (0.58–1.15)	0.247	1.18 (1.05–1.34)	**0**.**007**	1.07 (0.98–1.17)	0.132	1.08 (0.98–1.19)	0.106	1.03 (0.95–1.10)	0.500	1.03 (0.86–1.23)	0.755	0.93 (0.78–1.10)	0.374
Lisinopril vs. amlodipine	1.15 (0.98–1.34)	0.082	1.06 (0.95–1.19)	0.289	1.63 (1.17–3.20)	**0**.**010**	1.63 (1.14–2.33)	**0**.**007**	0.89 (0.77–1.03)	0.106	0.90 (0.81–1.00)	**0**.**042**	0.97 (0.87–1.08)	0.612	0.95 (0.87–1.03)	0.189	1.02 (0.83–1.24)	0.884	1.07 (0.88–1.29)	0.498
Lisinopril vs. chlorthalidone^b^	1.19 (1.03–1.36)	**0**.**015**	1.05 (0.95–1.16)	0.377	1.33 (1.13–2.60)	**0**.**011**	1.33 (1.00–1.79)	0.053	1.05 (0.93–1.20)	0.428	0.96 (0.88–1.06)	0.433	1.05 (0.95–1.16)	0.307	0.97 (0.90–1.04)	0.424	1.04 (0.88–1.25)	0.627	0.99 (0.84–1.16)	0.893
Age group (as of 1 January 1999) (years)																				
Age <70	1.00 (ref)		1.00 (ref)		1.00 (ref)		1.00 (ref)		1.00 (ref)		1.00 (ref)		1.00 (ref)		1.00 (ref)		1.00 (ref)		1.00 (ref)	
Age 70–79	1.09 (0.95–1.25)	0.238	1.40 (1.28–1.54)	**<0**.**001**	1.03 (0.50–1.13)	0.167	1.03 (0.78–1.35)	0.853	0.74 (0.66–0.83)	**<0**.**001**	0.97 (0.89–1.05)	0.470	0.82 (0.75–0.90)	**<0**.**001**	1.11 (1.04–1.18)	**0**.**003**	0.60 (0.51–0.70)	**<0**.**001**	0.71 (0.61–0.81)	**<0**.**001**
Age 80+	0.70 (0.58–0.84)	**<0**.**001**	1.75 (1.52–2.01)	**<0**.**001**	1.10 (0.22–0.71)	**0**.**002**	1.10 (0.70–1.74)	0.669	0.38 (0.32–0.46)	**<0**.**001**	0.90 (0.78–1.03)	0.117	0.65 (0.57–0.73)	**<0**.**001**	1.47 (1.34–1.62)	**<0**.**001**	0.21 (0.16–0.29)	**<0**.**001**	0.38 (0.28–0.52)	**<0**.**001**
Gender																				
Female^c^ vs. male	0.93 (0.82–1.05)	0.238	0.71 (0.64–0.77)	**<0**.**001**	1.32 (0.90–2.06)	0.148	1.32 (0.97–1.80)	0.073	1.25 (1.11–1.41)	**<0**.**001**	1.14 (1.05–1.25)	**0**.**003**	1.52 (1.39–1.67)	**<0**.**001**	1.57 (1.46–1.69)	**<0**.**001**	—	—	—	—
Race/ethnicity																				
Black vs. non-Black	1.13 (0.99–1.29)	0.070	1.07 (0.97–1.18)	0.149	2.64 (1.25–2.85)	**0**.**002**	2.64 (1.98–3.54)	**<0**.**001**	0.66 (0.58–0.75)	**<0**.**001**	0.64 (0.58–0.70)	**<0**.**001**	0.73 (0.67–0.81)	**<0**.**001**	0.68 (0.63–0.73)	**<0**.**001**	1.04 (0.86–1.24)	0.710	1.30 (1.10–1.53)	**0**.**002**
Hispanic/Latino ethnicity																				
Hispanic vs. non-Hispanic	0.85 (0.71–1.01)	0.068	0.52 (0.45–0.60)	**<0**.**001**	0.68 (0.55–1.71)	0.903	0.68 (0.44–1.05)	0.086	1.05 (0.91–1.21)	0.497	0.69 (0.62–0.77)	**<0**.**001**	0.93 (0.83–1.04)	0.197	0.62 (0.57–0.68)	**<0**.**001**	0.95 (0.76–1.17)	0.604	0.90 (0.74–1.09)	0.285
Education level																				
Less than high school vs. more	0.96 (0.83–1.10)	0.533	1.12 (1.02–1.24)	**0**.**019**	0.83 (0.50–1.18)	0.223	0.83 (0.62–1.12)	0.221	0.83 (0.74–0.93)	**0**.**002**	0.97 (0.89–1.05)	0.444	0.90 (0.82–0.99)	**0**.**035**	1.02 (0.95–1.10)	0.524	0.82 (0.70–0.96)	**0**.**013**	0.94 (0.81–1.08)	0.370
Treatment with antihypertensive drugs prior to trial baseline																				
Treated vs. untreated	1.20 (0.96–1.50)	0.102	1.22 (1.05–1.42)	**0**.**012**	1.44 (0.47–1.79)	0.806	1.44 (0.83–2.48)	0.193	1.18 (0.97–1.44)	0.102	1.19 (1.03–1.37)	**0**.**015**	1.08 (0.93–1.25)	0.307	1.09 (0.97–1.21)	0.134	1.18 (0.91–1.53)	0.204	1.21 (0.96–1.53)	0.105
Aspirin use (as of 1 January 1999)																				
Yes vs. no	1.12 (0.99–1.27)	0.071	0.98 (0.89–1.07)	0.631	1.05 (0.85–1.87)	0.249	1.05 (0.78–1.40)	0.762	1.07 (0.96–1.20)	0.233	0.95 (0.87–1.03)	0.226	0.90 (0.83–0.99)	**0**.**022**	0.94 (0.88–1.01)	0.090	1.20 (1.02–1.40)	**0**.**026**	1.04 (0.90–1.21)	0.577
HDL cholesterol <35 mg/dl (as of 1 January 1999)																				
Yes vs. no	1.09 (0.94–1.27)	0.234	1.25 (1.12–1.40)	**<0**.**001**	1.00 (0.40–1.22)	0.207	1.00 (0.66–1.52)	0.984	0.75 (0.65–0.87)	**<0**.**001**	0.96 (0.86–1.07)	0.459	0.86 (0.77–0.96)	**0**.**008**	1.02 (0.94–1.11)	0.636	1.04 (0.88–1.23)	0.653	1.04 (0.89–1.22)	0.623
Cigarette smoking at trial baseline																				
Never smoker	1.00		1.00		1.00		1.00		1.00		1.00		1.00		1.00		1.00		1.00	
Current smoker	1.23 (1.04–1.46)	**0**.**013**	2.05 (1.82–2.31)	**<0**.**001**	1.25 (0.46–1.38)	0.409	1.25 (0.84–1.87)	0.276	0.59 (0.50–0.70)	**<0**.**001**	1.05 (0.93–1.19)	0.445	0.83 (0.74–0.94)	**0**.**002**	1.26 (1.15–1.39)	**<0**.**001**	0.48 (0.38–0.61)	**<0**.**001**	0.76 (0.61–0.95)	**0**.**014**
Former smoker	1.14 (0.99–1.32)	0.066	1.24 (1.12–1.37)	**<0**.**001**	1.15 (0.62–1.49)	0.853	1.15 (0.86–1.55)	0.344	0.98 (0.87–1.11)	0.773	1.08 (0.99–1.18)	0.074	0.95 (0.87–1.04)	0.303	1.12 (1.04–1.20)	**0**.**002**	0.81 (0.68–0.97)	**0**.**018**	0.94 (0.80–1.11)	0.473
Diabetes classification (as of 1 January 1999)																				
Diabetic vs. non-iabetic	0.73 (0.64–0.83)	**<0**.**001**	1.11 (1.02–1.22)	**0**.**020**	1.03 (0.45–1.00)	0.051	1.03 (0.78–1.36)	0.839	0.71 (0.63–0.80)	**<0**.**001**	1.06 (0.97–1.15)	0.208	0.91 (0.83–0.99)	**0**.**024**	1.22 (1.15–1.31)	**<0**.**001**	0.60 (0.51–0.70)	**<0**.**001**	0.80 (0.69–0.93)	**0**.**003**
History of CHD (as of 1 January 1999)																				
Yes vs. No	1.09 (0.92–1.28)	0.332	0.97 (0.85–1.09)	0.596	0.81 (0.63–1.81)	0.813	0.81 (0.55–1.19)	0.286	1.04 (0.89–1.20)	0.621	1.08 (0.97–1.21)	0.159	0.99 (0.89–1.11)	0.890	0.98 (0.89–1.07)	0.588	0.93 (0.74–1.16)	0.515	0.94 (0.77–1.16)	0.583
ASCVD at trial baseline																				
Yes vs. no	0.90 (0.75–1.09)	0.279	1.00 (0.87–1.14)	0.961	1.52 (0.60–1.95)	0.790	1.52 (1.01–2.30)	**0**.**045**	0.98 (0.83–1.16)	0.797	1.05 (0.93–1.20)	0.409	1.00 (0.88–1.13)	0.943	1.02 (0.92–1.12)	0.749	0.85 (0.67–1.07)	0.167	1.05 (0.84–1.31)	0.662
History of MI or stroke (as of 1 January 1999)																				
Yes vs. no	0.82 (0.70–0.96)	**0**.**012**	1.09 (0.97–1.23)	0.150	0.99 (0.41–1.11)	0.121	0.99 (0.68–1.43)	0.939	0.88 (0.76–1.01)	0.074	1.03 (0.93–1.15)	0.539	1.09 (0.98–1.21)	0.132	1.26 (1.16–1.37)	**<0**.**001**	0.72 (0.59–0.87)	**0**.**001**	0.81 (0.67–0.97)	**0**.**025**
History of CABG (as of 1 January 1999)																				
Yes vs. no	0.82 (0.68–1.00)	**0**.**046**	0.98 (0.85–1.13)	0.801	1.13 (0.63–2.06)	0.658	1.13 (0.71–1.79)	0.612	0.83 (0.70–0.99)	**0**.**035**	1.05 (0.93–1.19)	0.458	0.95 (0.84–1.09)	0.473	1.07 (0.97–1.18)	0.169	1.11 (0.88–1.40)	0.374	1.13 (0.91–1.39)	0.261
Other ASCVD at trial baseline																				
Yes vs. no	0.90 (0.76–1.07)	0.226	1.04 (0.92–1.17)	0.555	0.90 (0.48–1.40)	0.471	0.90 (0.61–1.31)	0.576	1.05 (0.90–1.21)	0.564	1.04 (0.93–1.17)	0.444	1.00 (0.89–1.12)	0.975	1.06 (0.98–1.16)	0.163	0.83 (0.68–1.03)	0.088	0.85 (0.70–1.03)	0.094
Major ST segment depression (as of 1 January 1999)																				
Yes vs. no	0.89 (0.71–1.12)	0.327	1.12 (0.95–1.32)	0.179	0.92 (0.43–1.71)	0.659	0.92 (0.55–1.55)	0.762	0.90 (0.74–1.11)	0.342	1.09 (0.93–1.26)	0.291	0.89 (0.76–1.05)	0.167	0.97 (0.85–1.09)	0.584	1.00 (0.75–1.32)	0.992	1.18 (0.91–1.54)	0.216
LVH by the Minnesota code (as of 1 January 1999)																				
Hard LVH vs. no/soft LVH	0.90 (0.69–1.18)	0.451	1.14 (0.92–1.41)	0.242	0.67 (0.22–1.66)	0.326	0.67 (0.31–1.44)	0.301	0.78 (0.58–1.05)	0.097	0.88 (0.70–1.10)	0.273	0.92 (0.76–1.13)	0.432	1.19 (1.01–1.39)	**0**.**034**	1.01 (0.69–1.48)	0.957	1.01 (0.70–1.45)	0.974
LLT participant																				
Yes vs. no	1.03 (0.90–1.17)	0.708	0.94 (0.85–1.04)	0.219	0.86 (0.53–1.30)	0.426	0.86 (0.63–1.16)	0.316	1.04 (0.92–1.18)	0.490	0.99 (0.91–1.09)	0.898	1.03 (0.94–1.13)	0.565	0.95 (0.88–1.02)	0.136	0.98 (0.82–1.17)	0.846	0.89 (0.76–1.05)	0.166
Obesity (BMI ≥ 30 kg/m^2^) at trial baseline																				
Yes vs. no	1.04 (0.92–1.17)	0.562	1.06 (0.97–1.15)	0.214	1.40 (0.91–1.96)	0.139	1.40 (1.07–1.83)	**0**.**015**	1.22 (1.09–1.36)	**<0**.**001**	1.24 (1.14–1.34)	**<0**.**001**	1.06 (0.98–1.16)	0.153	1.11 (1.04–1.18)	**0**.**001**	1.00 (0.85–1.17)	0.971	0.94 (0.81–1.08)	0.380
Blood pressure change from the trial baseline to the latest BP reading prior to 1/1/1999, per 10 mmHg																				
Systolic BP	0.99 (0.95–1.02)	0.462	1.00 (0.97–1.03)	0.936	1.01 (0.92–1.16)	0.593	1.01 (0.93–1.10)	0.790	1.00 (0.97–1.04)	0.874	1.01 (0.99–1.04)	0.390	1.00 (0.97–1.02)	0.950	1.00 (0.98–1.02)	0.778	0.99 (0.94–1.04)	0.601	1.02 (0.97–1.07)	0.373
Diastolic BP	1.02 (0.95–1.08)	0.602	1.01 (0.96–1.06)	0.663	1.04 (0.83–1.26)	0.817	1.04 (0.90–1.21)	0.574	1.01 (0.96–1.08)	0.644	1.00 (0.96–1.05)	0.983	1.01 (0.96–1.05)	0.762	1.00 (0.97–1.04)	0.975	1.05 (0.96–1.15)	0.260	1.00 (0.92–1.08)	0.970

ASCVD, atherosclerotic cardiovascular disease; CABG, coronary artery bypass graft; LVH, left ventricular hypertrophy; LLT, lipid-lowering trial; HR, hazard ratio; CI, confidence interval.

Cancer diagnoses excluded non-melanoma skin cancer and included patients from inpatient data only. 18-year time to event includes events occurring up to, but not including, year 19 (e.g., throughout 2017).

^a^ Adjusted for each covariate shown in each column.

^b^ Contrast estimates were garnered from the same model using a different reference group for the randomized group (chlorthalidone or amlodipine).

^c^ Estrogen use was evaluated in women only and therefore omitted from the multivariable models.

Values in bold are statistically significant.

There were some significant differences in the risk of angioedema and insomnia among these antihypertensive drug groups. For example, the adjusted hazard ratio of angioedema was statistically significantly elevated in those receiving lisinopril than in those receiving amlodipine (1.63, 1.17–3.20, for 6-year risk and 1.63, 1.14–2.33, for 18-year risk) and in those receiving chlorthalidone (1.33, 1.13–2.60, for 6-year risk and 1.33, 1.00–1.79, for 18-year risk). The adjusted hazard ratio of insomnia was statistically significantly higher in patients receiving chlorthalidone (1.18, 1.05–1.34, for 6-year risk) than in those receiving amlodipine and significantly decreased in those receiving lisinopril than in those receiving amlodipine (0.90, 0.81–1.00, for 18-year risk). There were no significant differences in the 6-year and 18-year risk of depression and erectile dysfunction (in men) among the three antihypertensive drug groups.

**Figure 2:**
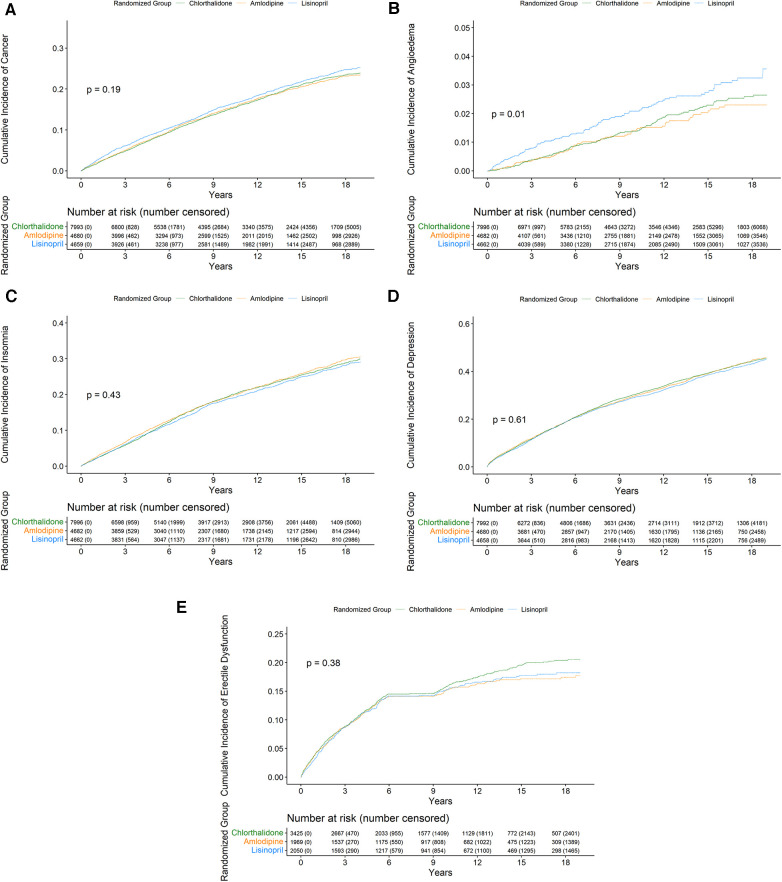
Cumulative incidence of cancer (**A**), angioedema (**B**), insomnia (**C**), depression (**D**), and erectile dysfunction (**E**) from 1999 to 2017 by the three study groups.

Figure 2 presents the Kaplan–Meier cumulative incidence rate curves of cancer, angioedema, insomnia, depression, and erectile dysfunction (Figures 2A–E) by the three study drugs from 1999 to 2017 using Medicare inpatient hospitalization data. The log-rank test was statistically significant only (*p* = 0.01) for the cumulative incidence rate curves of angioedema by the three drug groups but was not statistically significant in the other four cumulative incidence rate curves of insomnia, depression, cancer, and erectile dysfunction by the three study drug groups. In the Kaplan–Meier cumulative incidence rate curve of erectile dysfunction in men (Figure 2E), the plateau was observed between 6 and 9 years. In that time, there were 987 deaths and only two erectile dysfunction diagnoses from inpatient hospitalization data.

We also performed several sensitivity analyses by defining the study outcomes differently using Medicare inpatient, outpatient, and physician carrier claims data that occurred at least once (Supplementary Table S3A), using diagnosis codes that occurred at least two times 30 days apart from any diagnosis codes from Medicare inpatient claims only (Supplementary Table S3B), using any diagnosis codes from Medicare inpatient, outpatient, and physician carrier claims data (Supplementary Table S3C), using primary diagnosis only from Medicare inpatient claims alone (Supplementary Table S3D), or using primary diagnosis only from Medicare inpatient, outpatient, and physician carrier claims data (Supplementary Table S3E). The cumulative incidence rates of cancer, angioedema, insomnia, depression, and erectile dysfunction were higher when the diagnosis codes for outcomes were identified from Medicare inpatient, outpatient, and physician carrier claims and lower when the diagnosis codes for outcomes that required at least two claims 30 days apart or when the codes were restricted to primary diagnosis only in Medicare inpatient claims. However, regardless of the criteria for the five outcomes, findings from the three study drug groups were similar to those presented in Tables 2 and 3. Overall, there were no statistically significant differences in the adjusted hazard ratio of cancer, depression, and erectile dysfunction. The adjusted hazard ratio of angioedema was significantly higher in patients receiving lisinopril vs. amlodipine and marginally higher in those receiving lisinopril vs. chlorthalidone, whereas the adjusted hazard ratio of insomnia was significantly lower in patients receiving lisinopril vs. amlodipine (Supplementary Table S4).

Tables 2 and 3 also present the cumulative incidence rates and hazard ratios of cancer, angioedema, insomnia, depression, and erectile dysfunction by other sociodemographic and comorbidity factors. For example, the cumulative incidence rates of cancer and depression increased with advanced age, whereas the cumulative incidence rate of angioedema did not differ significantly with age and the cumulative incidence rates of insomnia and erectile dysfunction (in men) decreased with age (Table 2). The 6-year cumulative incidence rate of cancer was 7.8% in patients aged <70 years, 10.4% in patients aged 70–79 years, and 13.3% in patients aged ≥80 years, whereas the 18-year cumulative incidence rate of cancer was 21.6% in patients aged <70 years, 25.2% in patients aged 70–79 years, and 26.4% in patients aged ≥80 years. The adjusted 18-year risk of cancer was significantly higher in patients aged 70–79 years (hazard ratio: 1.40, 95% CI: 1.28–1.54) and ≥80 years (1.75, 1.52–2.01) (Table 3). There was no significant difference in the 18-year risk of angioedema with age, but the 18-year risk of depression was significantly higher in patients aged 70–79 years (hazard ratio: 1.11, 95% CI 1.04–1.18) and ≥80 years (1.47, 1.34–1.62), whereas the 18-year risk of insomnia and erectile dysfunction in men was lower in older patients. Women had a significantly lower risk of cancer but a significantly higher risk of insomnia and depression than men. Black patients were significantly more likely to have angioedema (2.64, 1.98–3.54) and erectile dysfunction (1.30, 1.10–1.53) and significantly less likely to have insomnia (0.64, 0.58–0.70) and depression (0.68, 0.63–0.73) than non-Black patients, but they did not have significantly different risk of cancer. Hispanics were significantly less likely to develop cancer (0.52, 0.45–0.60), insomnia (0.69, 0.62–0.77), and depression (0.62, 0.57–0.68), but they did not have a significantly different risk of angioedema and erectile dysfunction compared to non-Hispanics. Smoking (current or former), diabetes, myocardial infarction or stroke, left ventricular hypertrophy by the Minnesota code, and obesity were associated with a significantly higher risk of depression. Less than high school education, prior antihypertensive treatment, low HDL cholesterol, smoking (current or former), and diabetes were associated with a significantly higher risk of cancer. Diabetes, smoking (current), and a history of myocardial infarction or stroke were associated with a significantly lower risk of erectile dysfunction in men.

## Discussion

This study examined the 6-year and 18-year risk of cancer, angioedema, insomnia, depression, and erectile dysfunction in association with antihypertensive drugs by the three study groups and other factors among a large number of ALLHAT participants using the passive follow-up data of ALLHAT and Medicare-linked data from 1999 to 2017. We found that the risk of angioedema was significantly higher in patients receiving lisinopril than in those receiving amlodipine or chlorthalidone; the risk of insomnia was significantly lower in patients receiving lisinopril than in those receiving amlodipine; and the risk of cancer, depression, and erectile dysfunction (in men) was not statistically significantly different among the three drug groups. The risk of cancer and depression increased with advanced age, while the risk of insomnia and erectile dysfunction (in men) decreased with age; however, the risk of angioedema did not vary significantly with age. The risk of some of these outcomes was also associated with gender, race/ethnicity, education, smoking, and comorbidities.

Hypertension is a prevalent medical condition, accounting for 47% of adults in the United States in 2017–2018 and 33% of adults aged 30–79 years globally in 2019 (18–20). Many patients with hypertension need pharmaceutical treatment to have their blood pressure under control. Thankfully, there are multiple classes of antihypertensive drugs available, which are essential to treat hypertension and prevent more serious complications related to hypertension. However, all medications have a certain degree of side effects, and antihypertensive drugs are no exceptions (21–43). The important consideration in choosing various classes of antihypertensive drugs includes a well-accepted tolerance and a well-maintained quality of life (21–24). Some common side effects of antihypertensive drugs include dizziness, GI bleeding, hypotension, angioedema, insomnia, depression, and erectile dysfunction, depending on different antihypertensive drugs and duration of therapies (1, 2, 9–17, 25–43). Clinical trials often routinely report the side effects (adverse events or toxicities) of intervention drugs detected during the in-trial periods. Indeed, ALLHAT (1, 2) and other clinical trials such as the Systolic Blood Pressure Intervention Trial (SPRINT) (11, 12) reported some common serious side effects associated with antihypertensive drugs, including GI bleeding, angioedema, hypotension, syncope, and acute kidney injury or acute renal failure. However, due to short follow-ups, clinical trials are typically not well suited for measuring rare side effects and/or late (long-term) side effects. The literature that examined the potential links between antihypertensive drugs and the increased risks of side effects suggested that the use of antihypertensive drugs may also be associated with an increased risk of cancer, insomnia, depression, and erectile dysfunction.

An ALLHAT in-trial report did not find a significant association between amlodipine or lisinopril vs. chlorthalidone regarding the risk of GI bleeding (1, 2). A more recent ALLHAT study, which specifically focused on the risk of hospitalized GI bleeding using Medicare inpatient data concluded that hypertensive patients on amlodipine did not have an increased risk of GI bleeding compared to those on chlorthalidone or lisinopril (9). We later used the ALLHAT–Medicare-linked data to examine the risk of both hospitalized and non-hospitalized GI bleeding in association with the use of three study antihypertensive drugs (lisinopril, amlodipine, and chlorthalidone) (10). We found that the cumulative incidence rate of hospitalized GI bleeding until 31 March 2002 (the end of the ALLHAT in-trial) was 5.4%, 5.8%, and 5.4% for amlodipine, lisinopril, and chlorthalidone groups, and the cumulative incidence rate of non-hospitalized GI bleeding was 12.0%, 12.2%, and 12.0% for amlodipine, lisinopril, and chlorthalidone, respectively, which were not statistically significant among the three groups after adjusting for confounders in Cox regression models (10).

The link between the use of antihypertensive drugs and an increased risk of cancer was proposed but is still inconsistent (37, 38). Bangalore et al. summarized 70 clinical trials and concluded that an increased risk of cancer with the combination of ACE inhibitors and angiotensin-receptor blockers (ARBs) cannot be ruled out (37). Later, Copland et al. summarized 33 clinical trials and found no consistent evidence that antihypertensive medication use had any effect on cancer risk (38). The ALLHAT study also reported the 6-year rate of cancer (other than non-melanoma skin cancer) at 9.7% for chlorthalidone, 10.0% for amlodipine, and 9.9% for lisinopril. Our study using ALLHAT–Medicare-linked data had similar findings on the 6-year cumulative incidence rate of cancer (other than non-melanoma skin cancer) at 9.5%, 9.8%, and 10.5%, respectively, for the above drugs from Medicare inpatient hospitalization data. In addition, our study examined that by adding cases from outpatient and physician claims data, the 6-year rate of cancer (other than non-melanoma skin cancer) was much higher than that of using inpatient hospitalization data alone at 38.5% for chlorthalidone, 38.5% for amlodipine, and 39.3% for lisinopril, although the adjusted hazard ratio of cancer was not statistically significantly different between each pair of the three drugs (Supplementary Table S4).

Several studies suggested an association between antihypertensive drugs and depression (15, 16, 34–36) and sexual dysfunction (13, 14, 39–43). This study also found that there were no statistically significant differences in the 6-year and 18-year risk of depression and erectile dysfunction between the three drugs. However, our study did find that the risk of angioedema was statistically significantly increased in those receiving lisinopril than in those receiving amlodipine or chlorthalidone and the risk of insomnia was statistically significantly lower in those receiving lisinopril than in those receiving amlodipine. Several previous studies reported that ACE inhibitors, such as lisinopril, were related to an increased risk of angioedema mainly due to inhibition of the angiotensin-converting enzyme and subsequent blockade of bradykinin degradation (25–29). The weighted incidence rate of angioedema polled from over 40 trials was 0.3%, which was remarkably identical to 0.3% for lisinopril in our study (Table 2). Our study also found that those receiving lisinopril were significantly more likely to develop angioedema than those receiving calcium channel blockers (amlodipine) or a thiazide-type diuretic (chlorthalidone). Although some studies showed that there was a significant association between hypertension and insomnia (30–33) and that insomnia was considered a side-effect of both lisinopril and amlodipine (30–33, 44, 45), little information was available to compare the risk of insomnia between these antihypertensive drugs. Our study that found a lower risk in those receiving lisinopril than those receiving amlodipine may stimulate more research on this comparison. If it is confirmed, it will be of clinical importance and implications to patients and providers. Furthermore, previous studies demonstrated that the incidence and prevalence of erectile dysfunction increase with age (46, 47). However, our study showed that the risk of insomnia and erectile dysfunction decreased with age. This finding may require some caution for its interpretation because patients may not volunteer this information or significantly under-report their private health conditions to the providers.

Our study has several limitations. First, we only studied the trial participants who were still alive in 1999 and enrolled in Medicare insurance program, due to which the trial randomization was no longer intact and any analyses done off-randomization may be subject to unmeasured or unknown confounders. Although the distribution of all baseline characteristics among the three study drug groups by 1999 was not significantly different, unmeasured or unknown confounders cannot be ruled out. Also, because there was no information on actual blood pressure measurements after the trial ended in 2002, it was unknown whether the study outcomes were affected by changes in blood pressure. Second, subjects who were free of outcomes at baseline were determined from Medicare inpatient data only in January 1994–December 1998, which might have missed some cases. The underestimates were particularly possible on the outcomes such as insomnia, depression, and erectile dysfunction, which mostly relied on self-reporting during the medical encounters or more objective measures. Therefore, caution should be needed in determining the incidence rates of these outcomes, especially insomnia and erectile dysfunction, for which the risk expectedly decreased with age, although it may not be different among the three study groups. Third, although the 6-year cumulative incidence rate of outcomes such as cancer from Medicare inpatient data was identical to that of an original ALLHAT report (1), the incidence of outcomes from a complete Medicare data set (inpatient, outpatient, and physician carrier data) in this study could not be compared with another source because of the lack of such analysis from original ALLHAT reports. On the other hand, this study demonstrated that the incidence of these outcomes could be underestimated or missed if only Medicare inpatient data were used to ascertain the outcomes, even though those outcomes might not vary significantly by the three study drugs. Fourth, this study only compared the risk of five outcomes among the three study drug groups, but it is unknown about the gap in the risk of these outcomes between antihypertensive drug users and nonusers or what was the attributable risk of these outcomes due to antihypertensive drug use. Fifth, the study excluded trial participants from Canada and Veteran Affairs (VA) because they were ineligible for Medicare or had incomplete Medicare claims; the findings may just be generalizable to Medicare beneficiaries from the United States.

In conclusion, the 6-year and 18-year risk of angioedema was significantly higher in patients receiving lisinopril than in those receiving amlodipine or chlorthalidone; the risk of insomnia was significantly lower in patients receiving lisinopril than in those receiving amlodipine; and the risk of cancer, depression, and erectile dysfunction (in men) was not statistically significantly different among the three drug groups. The risk of cancer and depression increased with advanced age, while the risk of insomnia and erectile dysfunction (in men) decreased with age; however, the risk of angioedema did not vary significantly with age. The findings on the unique and long-term incidence of cancer, angioedema, insomnia, depression, and erectile dysfunction associated with various antihypertensive drugs would be helpful to care providers and patients with hypertension in their choice and management of antihypertensive medications.

## Data Availability

The data sets presented in this article are not readily available because the ALLHAT data and Medicare claims data are not public-use data sets. However, researchers may request the ALLHAT data with the approval from the ALLHAT Coordinating Center in Houston and the Medicare claims data with the approval from the Center for Medicare and Medicaid Services (CMS). The authors plan to share the statistical models and statistical programs that they used to analyze these data upon request. Requests to access the data sets should be directed to the ALLHAT Coordinating Center in Houston, School of Public Health, The University of Texas Health Science Center at Houston, 1200 Pressler Street, Houston, TX 77030, USA.
