# Supplementary material for: The 18-year risk of cancer, angioedema, insomnia, depression, and erectile dysfunction in association with antihypertensive drugs: post-trial analyses from ALLHAT–Medicare linked data
Source: Front Cardiovasc Med. 2023 Nov 17;10:1272385. doi: 10.3389/fcvm.2023.1272385 (PMC10691487; doi:10.3389/fcvm.2023.1272385)
Supplement: Supplementary file 1 [file Datasheet1.pdf]

Supplemental Materials:

Table S1a: Baseline Characteristics for Angioedema Outcome Cohort; Full Sample and by Randomized Group					
	Participants, N (%) (Unless otherwise indicated)				
	Full Sample	Chlorthalidone	Amlodipine	Lisinopril	P value*†
**Eligible for further follow-up as of 1/1/1999**	17,340	7,996	4,682	4,662	
**Hospitalized Angioedema diagnosis (1999-2017)**	68 (0.4)	31 (0.4)	18 (0.4)	19 (0.4)	0.980
**Non-hospitalized Angioedema diagnosis (1999-2017)**	282 (1.6)	118 (1.5)	66 (1.4)	98 (2.1)	**0.011**
**Hospitalized or Non-hospitalized Angioedema diagnosis (1999-2017)**	296 (1.7)	126 (1.6)	68 (1.5)	102 (2.2)	**0.011**
**Age, mean (SD), years**	73.43 (6.34)	73.37 (6.30)	73.48 (6.39)	73.50 (6.36)	0.495
**Age group (as of 1/1/1999)**					
Age <70	6037 (34.8)	2822 (35.3)	1612 (34.4)	1603 (34.4)	0.561
Age 70-79	8494 (49.0)	3916 (49.0)	2291 (48.9)	2287 (49.1)	
Age 80+	2809 (16.2)	1258 (15.7)	779 (16.6)	772 (16.6)	
**Gender**					
Male	7444 (42.9)	3425 (42.8)	1969 (42.1)	2050 (44.0)	0.168
Female	9896 (57.1)	4571 (57.2)	2713 (57.9)	2612 (56.0)	
**Race/Ethnicity**					
Black	11316 (65.3)	5227 (65.4)	3048 (65.1)	3041 (65.2)	0.952
Non-Black	6024 (34.7)	2769 (34.6)	1634 (34.9)	1621 (34.8)	
**Hispanic/Latino Ethnicity**					
Hispanic	3631 (21.0)	1668 (21.0)	959 (20.6)	1004 (21.7)	0.440
Non-Hispanic	13620 (79.0)	6291 (79.0)	3697 (79.4)	3632 (78.3)	
**Education, mean (SD), years**	10.57 (4.22)	10.60 (4.20)	10.54 (4.13)	10.56 (4.33)	0.742
**Education level**					
High school or less	11880 (74.1)	5457 (73.7)	3227 (74.5)	3196 (74.3)	0.554
More than high school	4154 (25.9)	1948 (26.3)	1103 (25.5)	1103 (25.7)	
**Treatment with antihypertensive drugs prior to trial baseline**					
Treated	15717 (90.6)	7216 (90.2)	4259 (91.0)	4242 (91.0)	0.255
Untreated	1623 (9.4)	780 (9.8)	423 (9.0)	420 (9.0)	
**Aspirin use (as of 1/1/1999)**	6410 (37.3)	2963 (37.4)	1698 (36.6)	1749 (37.9)	0.424
**Women taking estrogen at trial baseline**	1444 (14.9)	695 (15.5)	385 (14.4)	364 (14.2)	0.255
**HDL cholesterol (as of 1/1/1999), mean (SD), mg/dl**	48.15 (14.84)	48.08 (14.84)	48.58 (15.09)	47.85 (14.59)	0.058
**HDL <35 mg/dl (as of 1/1/1999)**	2784 (16.1)	1290 (16.1)	713 (15.2)	781 (16.8)	0.129
**Cigarette smoking at trial baseline**					
Never smoker	2845 (16.4)	1327 (16.6)	790 (16.9)	728 (15.6)	0.286
Current Smoker	6845 (39.5)	3169 (39.6)	1801 (38.5)	1875 (40.2)	
Former Smoker	7649 (44.1)	3500 (43.8)	2091 (44.7)	2058 (44.2)	
**Diabetes classification (as of 1/1/1999)**					

Diabetes	7091 (44.1)	3288 (44.3)	1917 (44.3)	1886 (43.7)	0.774
Non-diabetes	8981 (55.9)	4135 (55.7)	2412 (55.7)	2434 (56.3)	
**History of Coronary Heart Disease (CHD) (as of 1/1/1999)**	4848 (28.0)	2276 (28.5)	1261 (26.9)	1311 (28.1)	0.172
**Atherosclerotic Cardiovascular Disease (ASCVD) at trial baseline**	9482 (54.7)	4419 (55.3)	2491 (53.2)	2572 (55.2)	0.059
**History of myocardial infarction (MI) or stroke (as of 1/1/1999)**	4705 (27.1)	2167 (27.1)	1276 (27.3)	1262 (27.1)	0.976
**History of coronary artery bypass graft (CABG) (as of 1/1/1999)**	2677 (15.4)	1244 (15.6)	678 (14.5)	755 (16.2)	0.067
**Other ASCVD at trial baseline**	4567 (26.3)	2104 (26.3)	1216 (26.0)	1247 (26.7)	0.694
**Major ST segment depression (as of 1/1/1999)**	1389 (8.0)	667 (8.4)	337 (7.2)	385 (8.3)	0.055
**Hard Left ventricular hypertrophy (LVH) by Minnesota code (as of 1/1/1999)**	675 (4.4)	314 (4.4)	182 (4.4)	179 (4.3)	0.983
**Lipid Lowering Trial (LLT) Participants**	4219 (24.3)	1980 (24.8)	1150 (24.6)	1089 (23.4)	0.189
**BMI, mean (SD), kg/m^2 at trial baseline**	29.39 (5.98)	29.33 (6.02)	29.42 (5.97)	29.44 (5.92)	0.567
**Obesity (BMI ≥ 30 kg/m^2) at trial baseline**	6881 (39.7)	3155 (39.5)	1860 (39.7)	1866 (40.0)	0.818
**Latest blood pressure (BP) reading prior to 1/1/1999, mmHg**					
Systolic BP, mean (SD)	140.08 (16.59)	139.22 (16.24)	140.12 (15.71)	141.53 (17.88)	**<0.001**
Diastolic BP, mean (SD)	78.90 (10.04)	79.01 (9.91)	78.32 (9.86)	79.30 (10.41)	**<0.001**
**Blood pressure change from baseline to the latest BP reading prior to 1/1/1999, mmHg**					
Systolic BP, mean (SD)	-7.22 (18.39)	-8.10 (17.96)	-6.91 (18.25)	-6.03 (19.18)	**<0.001**
Diastolic BP, mean (SD)	-4.43 (10.57)	-4.51 (10.48)	-4.82 (10.48)	-3.90 (10.79)	**<0.001**

N= number of participants; SD= standard deviation
* P values represent significance level of the chi-square test of independence between randomized groups for binary and categorical variables, or the one-way analysis of variance (ANOVA) between randomized groups for continuous variables.
† Statistically significant p values (<0.05) are shown in bold text.

Table S1b: Baseline Characteristics for Insomnia Outcome Cohort; Full Sample and by Randomized Group

Participants, N (%) (Unless otherwise indicated)					
	Full Sample	Chlorthalidone	Amlodipine	Lisinopril	P value*†
**Eligible for further follow-up as of 1/1/1999**	17,340	7,996	4,682	4,662	
**Hospitalized Insomnia diagnosis (1999-2017)**	290 (1.7)	134 (1.7)	74 (1.6)	82 (1.8)	0.797
**Non-hospitalized Insomnia diagnosis (1999-2017)**	3283 (18.9)	1515 (18.9)	910 (19.4)	858 (18.4)	0.444
**Hospitalized or Non-hospitalized Insomnia diagnosis (1999-2017)**	3359 (19.4)	1547 (19.3)	934 (19.9)	878 (18.8)	0.393
**Age, mean (SD), years**	73.43 (6.34)	73.37 (6.30)	73.48 (6.39)	73.50 (6.36)	0.495
**Age group (as of 1/1/1999)**					
Age <70	6037 (34.8)	2822 (35.3)	1612 (34.4)	1603 (34.4)	0.561
Age 70-79	8494 (49.0)	3916 (49.0)	2291 (48.9)	2287 (49.1)	
Age 80+	2809 (16.2)	1258 (15.7)	779 (16.6)	772 (16.6)	
**Gender**					
Male	7444 (42.9)	3425 (42.8)	1969 (42.1)	2050 (44.0)	0.168
Female	9896 (57.1)	4571 (57.2)	2713 (57.9)	2612 (56.0)	
**Race/Ethnicity**					
Black	11316 (65.3)	5227 (65.4)	3048 (65.1)	3041 (65.2)	0.952
Non-Black	6024 (34.7)	2769 (34.6)	1634 (34.9)	1621 (34.8)	
**Hispanic/Latino Ethnicity**					
Hispanic	3631 (21.0)	1668 (21.0)	959 (20.6)	1004 (21.7)	0.440
Non-Hispanic	13620 (79.0)	6291 (79.0)	3697 (79.4)	3632 (78.3)	
**Education, mean (SD), years**	10.57 (4.22)	10.60 (4.20)	10.54 (4.13)	10.56 (4.33)	0.742
**Education level**					
High school or less	11880 (74.1)	5457 (73.7)	3227 (74.5)	3196 (74.3)	0.554
More than high school	4154 (25.9)	1948 (26.3)	1103 (25.5)	1103 (25.7)	
**Treatment with antihypertensive drugs prior to trial baseline**					
Treated	15717 (90.6)	7216 (90.2)	4259 (91.0)	4242 (91.0)	0.255
Untreated	1623 (9.4)	780 (9.8)	423 (9.0)	420 (9.0)	
**Aspirin use (as of 1/1/1999)**	6410 (37.3)	2963 (37.4)	1698 (36.6)	1749 (37.9)	0.424
**Women taking estrogen at trial baseline**	1444 (14.9)	695 (15.5)	385 (14.4)	364 (14.2)	0.255
**HDL cholesterol (as of 1/1/1999), mean (SD), mg/dl**	48.15 (14.84)	48.08 (14.84)	48.58 (15.09)	47.85 (14.59)	0.058
**HDL <35 mg/dl (as of 1/1/1999)**	2784 (16.1)	1290 (16.1)	713 (15.2)	781 (16.8)	0.129
**Cigarette smoking at trial baseline**					
Never smoker	2845 (16.4)	1327 (16.6)	790 (16.9)	728 (15.6)	0.286
Current Smoker	6845 (39.5)	3169 (39.6)	1801 (38.5)	1875 (40.2)	
Former Smoker	7649 (44.1)	3500 (43.8)	2091 (44.7)	2058 (44.2)	
**Diabetes classification (as of 1/1/1999)**					
Diabetes	7091 (44.1)	3288 (44.3)	1917 (44.3)	1886 (43.7)	0.774
Non-diabetes	8981 (55.9)	4135 (55.7)	2412 (55.7)	2434 (56.3)	
**History of Coronary Heart Disease (CHD) (as of 1/1/1999)**	4848 (28.0)	2276 (28.5)	1261 (26.9)	1311 (28.1)	0.172

**Atherosclerotic Cardiovascular Disease (ASCVD) at trial baseline**	9482 (54.7)	4419 (55.3)	2491 (53.2)	2572 (55.2)	0.059
**History of myocardial infarction (MI) or stroke (as of 1/1/1999)**	4705 (27.1)	2167 (27.1)	1276 (27.3)	1262 (27.1)	0.976
**History of coronary artery bypass graft (CABG) (as of 1/1/1999)**	2677 (15.4)	1244 (15.6)	678 (14.5)	755 (16.2)	0.067
**Other ASCVD at trial baseline**	4567 (26.3)	2104 (26.3)	1216 (26.0)	1247 (26.7)	0.694
**Major ST segment depression (as of 1/1/1999)**	1389 (8.0)	667 (8.4)	337 (7.2)	385 (8.3)	0.055
**Hard Left ventricular hypertrophy (LVH) by Minnesota code (as of 1/1/1999)**	675 (4.4)	314 (4.4)	182 (4.4)	179 (4.3)	0.983
**Lipid Lowering Trial (LLT) Participants**	4219 (24.3)	1980 (24.8)	1150 (24.6)	1089 (23.4)	0.189
**BMI, mean (SD), kg/m^2 at trial baseline**	29.39 (5.98)	29.33 (6.02)	29.42 (5.97)	29.44 (5.92)	0.567
**Obesity (BMI ≥ 30 kg/m^2) at trial baseline**	6881 (39.7)	3155 (39.5)	1860 (39.7)	1866 (40.0)	0.818
**Latest blood pressure (BP) reading prior to 1/1/1999, mmHg**					
Systolic BP, mean (SD)	140.08 (16.59)	139.22 (16.24)	140.12 (15.71)	141.53 (17.88)	**<0.001**
Diastolic BP, mean (SD)	78.90 (10.04)	79.01 (9.91)	78.32 (9.86)	79.30 (10.41)	**<0.001**
**Blood pressure change from baseline to the latest BP reading prior to 1/1/1999, mmHg**					
Systolic BP, mean (SD)	-7.22 (18.39)	-8.10 (17.96)	-6.91 (18.25)	-6.03 (19.18)	**<0.001**
Diastolic BP, mean (SD)	-4.43 (10.57)	-4.51 (10.48)	-4.82 (10.48)	-3.90 (10.79)	**<0.001**

N= number of participants; SD= standard deviation
*** P values represent significance level of the chi-square test of independence between randomized groups for binary and categorical variables, or the one-way analysis of variance (ANOVA) between randomized groups for continuous variables.**
† Statistically significant p values (<0.05) are shown in bold text.

Table S1c: Baseline Characteristics for Depression Outcome Cohort; Full Sample and by Randomized Group

Participants, N (%) (Unless otherwise indicated)					
	**Full Sample**	**Chlorthalidone**	**Amlodipine**	**Lisinopril**	**P value*†**
**Eligible for further follow-up as of 1/1/1999**	17,330	7,992	4,680	4,658	
**Hospitalized Depression diagnosis (1999-2017)**	2017 (11.6)	943 (11.8)	544 (11.6)	530 (11.4)	0.775
**Non-hospitalized Depression diagnosis (1999-2017)**	5132 (29.6)	2372 (29.7)	1401 (29.9)	1359 (29.2)	0.712
**Hospitalized or Non-hospitalized Depression diagnosis (1999-2017)**	5456 (31.5)	2531 (31.7)	1487 (31.8)	1438 (30.9)	0.572
**Age, mean (SD), years**	73.43 (6.34)	73.37 (6.30)	73.47 (6.39)	73.49 (6.36)	0.505
**Age group (as of 1/1/1999)**					
Age <70	6035 (34.8)	2821 (35.3)	1612 (34.4)	1602 (34.4)	0.578
Age 70-79	8488 (49.0)	3913 (49.0)	2290 (48.9)	2285 (49.1)	
Age 80+	2807 (16.2)	1258 (15.7)	778 (16.6)	771 (16.6)	
**Gender**					
Male	7443 (42.9)	3425 (42.9)	1968 (42.1)	2050 (44.0)	0.157
Female	9887 (57.1)	4567 (57.1)	2712 (57.9)	2608 (56.0)	
**Race/Ethnicity**					
Black	11311 (65.3)	5225 (65.4)	3047 (65.1)	3039 (65.2)	0.952
Non-Black	6019 (34.7)	2767 (34.6)	1633 (34.9)	1619 (34.8)	
**Hispanic/Latino Ethnicity**					
Hispanic	3630 (21.1)	1667 (21.0)	959 (20.6)	1004 (21.7)	0.431
Non-Hispanic	13611 (78.9)	6288 (79.0)	3695 (79.4)	3628 (78.3)	
**Education, mean (SD), years**	10.57 (4.22)	10.60 (4.20)	10.54 (4.13)	10.56 (4.33)	0.740
**Education level**					
High school or less	11873 (74.1)	5455 (73.7)	3225 (74.5)	3193 (74.3)	0.569
More than high school	4153 (25.9)	1947 (26.3)	1103 (25.5)	1103 (25.7)	
**Treatment with antihypertensive drugs prior to trial baseline**					
Treated	15707 (90.6)	7212 (90.2)	4257 (91.0)	4238 (91.0)	0.257
Untreated	1623 (9.4)	780 (9.8)	423 (9.0)	420 (9.0)	
**Aspirin use (as of 1/1/1999)**	6406 (37.3)	2961 (37.4)	1696 (36.6)	1749 (37.9)	0.394
**Women taking estrogen at trial baseline**	1443 (14.9)	695 (15.5)	385 (14.4)	363 (14.2)	0.244
**HDL cholesterol (as of 1/1/1999), mean (SD), mg/dl**	48.15 (14.84)	48.08 (14.84)	48.58 (15.09)	47.84 (14.59)	0.054
**HDL <35 mg/dl (as of 1/1/1999)**	2784 (16.1)	1290 (16.1)	713 (15.2)	781 (16.8)	0.127
**Cigarette smoking at trial baseline**					
Never smoker	2843 (16.4)	1326 (16.6)	790 (16.9)	727 (15.6)	0.271
Current Smoker	6844 (39.5)	3169 (39.7)	1800 (38.5)	1875 (40.3)	
Former Smoker	7642 (44.1)	3497 (43.8)	2090 (44.7)	2055 (44.1)	
**Diabetes classification (as of 1/1/1999)**					
Diabetes	7086 (44.1)	3286 (44.3)	1917 (44.3)	1883 (43.6)	0.752
Non-diabetes	8976 (55.9)	4133 (55.7)	2410 (55.7)	2433 (56.4)	
**History of Coronary Heart Disease (CHD) (as of 1/1/1999)**	4844 (28.0)	2274 (28.5)	1259 (26.9)	1311 (28.1)	0.161

**Atherosclerotic Cardiovascular Disease (ASCVD) at trial baseline**	9478 (54.7)	4417 (55.3)	2489 (53.2)	2572 (55.2)	0.053
**History of myocardial infarction (MI) or stroke (as of 1/1/1999)**	4700 (27.1)	2165 (27.1)	1274 (27.2)	1261 (27.1)	0.983
**History of coronary artery bypass graft (CABG) (as of 1/1/1999)**	2676 (15.4)	1244 (15.6)	677 (14.5)	755 (16.2)	0.061
**Other ASCVD at trial baseline**	4565 (26.3)	2103 (26.3)	1215 (26.0)	1247 (26.8)	0.672
**Major ST segment depression (as of 1/1/1999)**	1389 (8.0)	667 (8.4)	337 (7.2)	385 (8.3)	0.054
**Hard Left ventricular hypertrophy (LVH) by Minnesota code (as of 1/1/1999)**	674 (4.4)	314 (4.4)	182 (4.4)	178 (4.3)	0.969
**Lipid Lowering Trial (LLT) Participants**	4218 (24.3)	1980 (24.8)	1149 (24.6)	1089 (23.4)	0.195
**BMI, mean (SD), kg/m^2 at trial baseline**	29.39 (5.98)	29.33 (6.02)	29.42 (5.98)	29.44 (5.92)	0.577
**Obesity (BMI ≥ 30 kg/m^2) at trial baseline**	6876 (39.7)	3154 (39.5)	1858 (39.7)	1864 (40.0)	0.828
**Latest blood pressure (BP) reading prior to 1/1/1999, mmHg**					
Systolic BP, mean (SD)	140.08 (16.59)	139.21 (16.25)	140.13 (15.71)	141.53 (17.88)	**<0.001**
Diastolic BP, mean (SD)	78.90 (10.04)	79.01 (9.90)	78.33 (9.86)	79.30 (10.41)	**<0.001**
**Blood pressure change from baseline to the latest BP reading prior to 1/1/1999, mmHg**					
Systolic BP, mean (SD)	-7.22 (18.40)	-8.10 (17.96)	-6.90 (18.25)	-6.03 (19.19)	**<0.001**
Diastolic BP, mean (SD)	-4.43 (10.57)	-4.51 (10.48)	-4.82 (10.48)	-3.90 (10.79)	**<0.001**

N= number of participants; SD= standard deviation
* P values represent significance level of the chi-square test of independence between randomized groups for binary and categorical variables, or the one-way analysis of variance (ANOVA) between randomized groups for continuous variables.
† Statistically significant p values (<0.05) are shown in bold text.

Table S1d: Baseline Characteristics for Erectile Dysfunction Outcome Cohort; Full Sample and by Randomized Group

	Participants, N (%) (Unless otherwise indicated)				
	Full Sample	Chlorthalidone	Amlodipine	Lisinopril	P value*†
**Eligible for further follow-up as of 1/1/1999**	7,444	3,425	1,969	2,050	
**Hospitalized Erectile Dysfunction diagnosis (1999-2017)**	58 (0.8)	25 (0.7)	10 (0.5)	23 (1.1)	0.078
**Non-hospitalized Erectile Dysfunction diagnosis (1999-2017)**	1055 (14.2)	502 (14.7)	272 (13.8)	281 (13.7)	0.540
**Hospitalized or Non-hospitalized Erectile Dysfunction diagnosis (1999-2017)**	1079 (14.5)	518 (15.1)	273 (13.9)	288 (14.0)	0.358
**Age, mean (SD), years**	72.93 (6.08)	72.79 (6.03)	72.99 (6.08)	73.11 (6.15)	0.143
**Age group (as of 1/1/1999)**					
Age <70	2749 (36.9)	1310 (38.2)	701 (35.6)	738 (36.0)	0.210
Age 70-79	3669 (49.3)	1667 (48.7)	986 (50.1)	1016 (49.6)	
Age 80+	1026 (13.8)	448 (13.1)	282 (14.3)	296 (14.4)	
**Gender ‡**					
Male	7444 (100.0)	3425 (100.0)	1969 (100.0)	2050 (100.0)	
Female	0	0	0	0	
**Race/Ethnicity**					
Black	5383 (72.3)	2471 (72.1)	1416 (71.9)	1496 (73.0)	0.721
Non-Black	2061 (27.7)	954 (27.9)	553 (28.1)	554 (27.0)	
**Hispanic/Latino Ethnicity**					
Hispanic	1448 (19.6)	653 (19.2)	373 (19.1)	422 (20.7)	0.312
Non-Hispanic	5948 (80.4)	2749 (80.8)	1584 (80.9)	1615 (79.3)	
**Education, mean (SD), years**	11.17 (4.42)	11.21 (4.44)	11.17 (4.37)	11.13 (4.43)	0.833
**Education level**					
High school or less	4631 (67.0)	2134 (66.8)	1231 (67.2)	1266 (66.9)	0.973
More than high school	2286 (33.0)	1059 (33.2)	602 (32.8)	625 (33.1)	
**Treatment with antihypertensive drugs prior to trial baseline**					
Treated	6629 (89.1)	3043 (88.8)	1761 (89.4)	1825 (89.0)	0.799
Untreated	815 (10.9)	382 (11.2)	208 (10.6)	225 (11.0)	
**Aspirin use (as of 1/1/1999)**	3356 (45.5)	1567 (46.2)	852 (43.7)	937 (46.2)	0.153
**Women taking estrogen at trial baseline ‡**	0	0	0	0	
**HDL cholesterol (as of 1/1/1999), mean (SD), mg/dl**	42.67 (12.34)	42.52 (12.24)	42.87 (12.31)	42.73 (12.53)	0.612
**HDL <35 mg/dl (as of 1/1/1999)**	1909 (25.6)	892 (26.0)	478 (24.3)	539 (26.3)	0.263
**Cigarette smoking at trial baseline**					
Never smoker	1389 (18.7)	634 (18.5)	382 (19.4)	373 (18.2)	0.681
Current Smoker	4159 (55.9)	1937 (56.6)	1084 (55.1)	1138 (55.5)	
Former Smoker	1895 (25.5)	854 (24.9)	503 (25.5)	538 (26.3)	
**Diabetes classification (as of 1/1/1999)**					
Diabetes	2889 (41.7)	1348 (42.1)	764 (42.0)	777 (40.9)	0.702
Non-diabetes	4031 (58.3)	1856 (57.9)	1054 (58.0)	1121 (59.1)	

**History of Coronary Heart Disease (CHD) (as of 1/1/1999)**	2593 (34.8)	1192 (34.8)	666 (33.8)	735 (35.9)	0.402
**Atherosclerotic Cardiovascular Disease (ASCVD) at trial baseline**	4351 (58.4)	2006 (58.6)	1126 (57.2)	1219 (59.5)	0.336
**History of myocardial infarction (MI) or stroke (as of 1/1/1999)**	2388 (32.1)	1087 (31.7)	625 (31.7)	676 (33.0)	0.594
**History of coronary artery bypass graft (CABG) (as of 1/1/1999)**	1721 (23.1)	795 (23.2)	439 (22.3)	487 (23.8)	0.539
**Other ASCVD at trial baseline**	1934 (26.0)	882 (25.8)	497 (25.2)	555 (27.1)	0.382
**Major ST segment depression (as of 1/1/1999)**	554 (7.5)	277 (8.1)	128 (6.5)	149 (7.3)	0.096
**Hard Left ventricular hypertrophy (LVH) by Minnesota code (as of 1/1/1999)**	266 (3.9)	126 (4.0)	68 (3.9)	72 (3.9)	0.941
**Lipid Lowering Trial (LLT) Participants**	1757 (23.6)	836 (24.4)	454 (23.1)	467 (22.8)	0.312
**BMI, mean (SD), kg/m^2 at trial baseline**	28.76 (5.01)	28.76 (5.00)	28.72 (4.87)	28.81 (5.16)	0.848
**Obesity (BMI ≥ 30 kg/m^2) at trial baseline**	2570 (34.5)	1168 (34.1)	679 (34.5)	723 (35.3)	0.679
**Latest blood pressure (BP) reading prior to 1/1/1999, mmHg**					
Systolic BP, mean (SD)	138.91 (16.28)	138.20 (15.96)	139.26 (15.39)	139.77 (17.55)	**0.001**
Diastolic BP, mean (SD)	79.33 (10.02)	79.46 (9.90)	79.07 (9.82)	79.37 (10.39)	0.389
**Blood pressure change from baseline to the latest BP reading prior to 1/1/1999, mmHg**					
Systolic BP, mean (SD)	-7.89 (17.88)	-8.52 (17.30)	-7.45 (17.87)	-7.25 (18.80)	**0.017**
Diastolic BP, mean (SD)	-4.83 (10.43)	-4.83 (10.33)	-4.96 (10.47)	-4.72 (10.55)	0.772

N= number of participants; SD= standard deviation
* P values represent significance level of the chi-square test of independence between randomized groups for binary and categorical variables, or the one-way analysis of variance (ANOVA) between randomized groups for continuous variables.
† Statistically significant p values (<0.05) are shown in bold text.
‡ Only males were eligible for follow-up in consideration of erectile dysfunction diagnoses.

Table S2a. ICD-9 and ICD-10 Codes for Angioedema, Insomnia, and Depression *

ICD-9 Codes	Description	ICD-10 Codes	Description
**995.1**	**Angioedema**	T78.3XXA	Angioneurotic edema, initial encounter
	**Insomnia:**		
**780.50**	Sleep disturbance, unspecified	G47.9	Sleep disorder, unspecified
**780.51**	Insomnia with sleep apnea, unspecified	G47.30	Sleep apnea, unspecified
**780.52**	Insomnia, unspecified	G47.00	Insomnia, unspecified
**780.53**	Hypersomnia with sleep apnea, unspecified	G47.30	Sleep apnea, unspecified
**780.54**	Hypersomnia, unspecified	G47.10	Hypersomnia, unspecified
	**Depression:**		
**296.2x**	Major depressive disorder single episode		
**296.20**	Major depressive affective disorder, single episode - unspecified	F32.9	Major depressive disorder, single episode, unspecified
**296.21**	Major depressive affective disorder single episode - mild degree	F32.0	Major depressive disorder, single episode, mild
**296.22**	Major depressive affective disorder single episode - moderate degree	F32.1	Major depressive disorder, single episode, moderate
**296.23**	Major depressive affective disorder single episode - severe degree without psychotic behavior	F32.2	Major depressive disorder, single episode, severe without psychotic features
**296.24**	Major depressive affective disorder single episode - severe degree specified as with psychotic behavior	F32.3	Major depressive disorder, single episode, severe with psychotic features
**296.25**	Major depressive affective disorder single episode in partial or unspecified remission	F32.4	Major depressive disorder, single episode, in partial remission
**296.26**	Major depressive affective disorder single episode in full remission	F32.5	Major depressive disorder, single episode, in full remission
**296.3**	Major depressive disorder recurrent episode		N/A
**296.30**	Major depressive affective disorder recurrent episode unspecified degree	F33.9	Major depressive disorder, recurrent, unspecified
**296.31**	Major depressive affective disorder recurrent episode mild degree	F33.0	Major depressive disorder, recurrent, mild
**296.32**	Major depressive affective disorder recurrent episode moderate degree	F33.1	Major depressive disorder, recurrent, moderate
**296.33**	Major depressive affective disorder recurrent episode severe degree without psychotic behavior	F33.2	Major depressive disorder, recurrent severe without psychotic features
**296.34**	Major depressive affective disorder recurrent episode severe degree specified as with psychotic behavior	F33.3	Major depressive disorder, recurrent, severe with psychotic symptoms
**296.35**	Major depressive affective disorder recurrent episode in partial or unspecified remission	F33.41	Major depressive disorder, recurrent, in partial remission
**296.36**	Major depressive affective disorder recurrent episode in full remission	F33.42	Major depressive disorder, recurrent, in full remission

**309.0**	Adjustment disorder with depressed mood	F43.21	Adjustment disorder with depressed mood
**309.1**	Prolonged depressive reaction	F43.21	Adjustment disorder with depressed mood
**309.24**	Adjustment disorder with anxiety	F43.22	Adjustment disorder with anxiety
**309.28**	Adjustment disorder with mixed anxiety and depressed mood	F43.23	Adjustment disorder with mixed anxiety and depressed mood
**309.29**	Other adjustment reactions with predominant disturbance of other emotions	F43.29	Adjustment disorder with other symptoms
**309.3**	Adjustment disorder with disturbance of conduct	F43.24	Adjustment disorder with disturbance of conduct
**309.4**	Adjustment disorder with mixed disturbance of emotions and conduct	F43.25	Adjustment disorder with mixed disturbance of emotions and conduct
**309.81**	Posttraumatic stress disorder	F43.10 or F43.12	Post-traumatic stress disorder, unspecified; or Post-traumatic stress disorder, chronic
**309.82**	Adjustment reaction with physical symptoms	F43.8	Other reactions to severe stress
**309.83**	Adjustment reaction with withdrawal	F43.8	Other reactions to severe stress
**309.89**	Other specified adjustment reactions	F43.8	Other reactions to severe stress
**309.9**	Unspecified adjustment reaction	F43.20	Adjustment disorder, unspecified
**311**	Depressive disorder, not elsewhere classified	F32.9	Major depressive disorder, single episode, unspecified

*Convert source online: https://www.icd10data.com/Convert, accessed 3/7/2019. Highlighted codes indicate ‘uncertain decision for the specified condition’.

Table S2b. ICD-9 and ICD-10 Codes for Cancer (all types of cancers combined except for non-Melanoma skin cancer)

ICD-9 Codes	Description	ICD-10 Codes	Description
**140-149**	Malignant Neoplasm Of Lip, Oral Cavity, And Pharynx	C00-C14	Malignant neoplasms of lip, oral cavity and pharynx
**150-159**	Malignant Neoplasm Of Digestive Organs And Peritoneum	C15-C26	Malignant neoplasms of digestive organs
**160-169**	Malignant Neoplasm Of Respiratory And Intrathoracic Organs	C30-C39	Malignant neoplasms of respiratory and intrathoracic organs
**170-176**	Malignant Neoplasm Of Bone, Connective Tissue, Skin, And Breast	C40-C41	Malignant neoplasms of bone and articular cartilage
		C43-C44	Melanoma and other malignant neoplasms of skin
		C45-C49	Malignant neoplasms of mesothelial and soft tissue
		C50-C50	Malignant neoplasms of breast
**179-189**	Malignant Neoplasm Of Genitourinary Organs	C51-C58	Malignant neoplasms of female genital organs
**190-199**	Malignant Neoplasm Of Other And Unspecified Sites	C60-C63	Malignant neoplasms of male genital organs
**200-209**	Malignant Neoplasm Of Lymphatic And Hematopoietic Tissue	C64-C68	Malignant neoplasms of urinary tract
**210-229**	Benign Neoplasms	C69-C72	Malignant neoplasms of eye, brain and other parts of central nervous system
		C73-C75	Malignant neoplasms of thyroid and other endocrine glands
		C76-C80	Malignant neoplasms of ill-defined, other secondary and unspecified sites
		C7A-C7A	Malignant neuroendocrine tumors
		C7B-C7B	Secondary neuroendocrine tumors
		C81-C96	Malignant neoplasms of lymphoid, hematopoietic and related tissue
**230-234**	Carcinoma In Situ	D00-D09	In situ neoplasms
		D10-D36	Benign neoplasms, except benign neuroendocrine tumors
**235-238**	Neoplasms Of Uncertain Behavior	D37-D48	Neoplasms of uncertain behavior, polycythemia vera and myelodysplastic syndromes
		D3A-D3A	Benign neuroendocrine tumors
**239-239**	Neoplasms Of Unspecified Nature	D49-D49	Neoplasms of unspecified behavior

*Convert source online: https://www.icd10data.com/Convert, accessed 10/22/2021.
ICD-9/10-CM: International Classification of Diseases, 9th/10th Revision, Clinical Modification.

Table S2c. ICD-9 and ICD-10 Codes Diagnosis and Procedure Codes for erectile dysfunction

ICD-9 Diagnosis Codes	Description	ICD-10 Diagnosis Codes	Description
**607.84**	Impotence of organic origin, Erectile dysfunction	N52.x	Male Erectile dysfunction
		F52	Sexual dysfunction not due to a substance or known physiological condition
		F52.2	Sexual arousal disorders
**302.72**	Psychosexual dysfunction with inhibited sexual excitement	F52.21	Male erectile disorder
Procedure codes:	**Description**		**Description**
ICD-9 procedure codes:		**ICD-10 procedure codes:**	
**64,x**	Operations on penis	0VUS0JZ	Supplement penis with synthetic substitute, open approach
		0VUS4JZ	Supplement penis with synthetic substitute, percutaneous endoscopic approach
CPT*: (**Common Procedure Code**)			
**54231**	Assessment of erectile dysfunction including injection of drugs into the penis		
**54235**	Under Introduction Procedures on the Penis		
**54400**	Insertion of penile prosthesis; non-inflatable (semi-rigid)		
**54401**	Insertion of penile prosthesis; inflatable (self-contained)		
**54402**	Removal or replacement of noninflatable (semi-rigid) or inflatable (selfcontained) penile prosthesis		
**54405**	Insertion of multi-component, inflatable penile prosthesis, including placement of pump, cylinders, and reservoir		
**54406**	Repair Procedures on the Penis		
**54407**	Removal, repair or replacement of inflatable (multi-component) penile prosthesis		
**54408**	Repair of components of a multi-component inflatable penile prosthesis		
**54410**	Removal and replacement of all component(s) of a multi-component, inflatable penile prosthesis at the same operative session		
**54411**	Removal and replacement of all components of a multi-component inflatable penile prosthesis through an infected field at the same operative session, including irrigation and debridement of infected tissue		
**54415**	Repair Procedures on the Penis		
**54416**	Removal and replacement of non-inflatable (semi-rigid) or inflatable (self-contained) penile prosthesis at the same operative session		
**54417**	Removal and replacement of a non-inflatable (semi-rigid) or inflatable (self-contained) penile prosthesis through an infected field at the same operative session, including irrigation and debridement of infected tissue		
HCPCS*: (Healthcare Common Procedure Coding System) codes:			
**C1813**	Prosthesis, penile, inflatable		
**C2622**	Prosthesis, penile, non-inflatable		
**L7900**	Male vacuum erection system		
**L8699**	Prosthetic implant, not otherwise specified		

*ICD-9/10-CM: International Classification of Diseases, 9th/10th Revision, Clinical Modification. CPT: Common Procedure Terminology.
HCPCS: Healthcare Common Procedure Coding System.

Table S3a: Cumulative Incidence (%) of 5 outcomes from any diagnosis in Medicare Inpatient, Outpatient and Physician Carries claims on ≥1 time by the 3 study drugs and other factors (1999 to 2017)

Demographic	Cancer			Angioedema			Insomnia			Depression			Erectile Dysfunction		
	Events/ Total (n/N)	6-Year % (SE)	18-Year % (SE)	Events/ Total (n/N)	6-Year % (SE)	18-Year % (SE)	Events/ Total (n/N)	6-Year % (SE)	18-Year % (SE)	Events/ Total n/N	6-Year % (SE)	18-Year % (SE)	Events/ Total (n/N)	6-Year % (SE)	18-Year % (SE)
All Patients	8512/17329	38.7 (0.4)	61.4 (0.5)	296/17340	1 (0.1)	2.8 (0.2)	3337/17340	12.3 (0.3)	29.7 (0.5)	5419/17330	20.6 (0.3)	45.2 (0.5)	1008/7444	13.2 (0.4)	18 (0.6)
**Randomized group**															
Chlorthalidone	3887/7992	38.5 (0.6)	61.1 (0.7)	126/7996	0.9 (0.1)	2.7 (0.3)	1539/7996	12.3 (0.4)	29.8 (0.7)	2515/7992	20.8 (0.5)	45.2 (0.8)	486/3425	13.5 (0.6)	19.4 (0.9)
Amlodipine	2281/4678	38.5 (0.7)	60.4 (0.9)	68/4682	0.9 (0.2)	2.3 (0.3)	926/4682	12.8 (0.5)	30.3 (1.0)	1478/4680	20.6 (0.6)	45.6 (1.0)	252/1969	12.9 (0.8)	16.6 (1.1)
Lisinopril	2344/4659	39.3 (0.8)	62.7 (0.9)	102/4662	1.3 (0.2)	3.6 (0.4)	872/4662	11.6 (0.5)	29.0 (0.9)	1426/4658	20.4 (0.6)	44.8 (1.0)	270/2050	13.0 (0.8)	17.1 (1.0)
**Age group (as of 1/1/1999)**															
Age <70	3043/6035	34.6 (0.6)	59.7 (0.7)	116/6037	0.9 (0.1)	2.8 (0.3)	1362/6037	12.0 (0.4)	30.7 (0.7)	1979/6035	18.7 (0.5)	42.5 (0.8)	510/2749	16.8 (0.7)	22.5 (0.9)
Age 70-79	4271/8487	40.3 (0.6)	61.7 (0.7)	147/8494	1.1 (0.1)	2.8 (0.3)	1607/8494	12.3 (0.4)	29.2 (0.7)	2653/8488	20.4 (0.5)	46.1 (0.8)	440/3669	11.7 (0.6)	15.8 (0.8)
Age 80+	1198/2807	43.5 (1.1)	60.9 (1.5)	33/2809	1.0 (0.2)	2.3 (0.5)	368/2809	12.6 (0.7)	25.4 (1.6)	787/2807	26.7 (1.0)	48.4 (1.8)	58/1026	7.3 (1.0)	7.9 (1.1)
**Gender**															
Male	4205/7437	48.0 (0.6)	70.1 (0.7)	95/7444	0.9 (0.1)	2.1 (0.2)	1345/7444	11.9 (0.4)	30.0 (0.8)	1885/7443	16.2 (0.5)	40.2 (0.8)	---	---	---
Female	4307/9892	31.8 (0.5)	55.1 (0.6)	201/9896	1.1 (0.1)	3.3 (0.2)	1992/9896	12.5 (0.4)	29.7 (0.6)	3534/9887	23.9 (0.5)	48.7 (0.7)	---	---	---
**Race/Ethnicity**															
Black	2606/6019	32.1 (0.6)	55.8 (0.8)	173/6024	1.5 (0.2)	4.9 (0.4)	918/6024	9.2 (0.4)	24.3 (0.8)	1670/6019	17.8 (0.5)	41.7 (0.9)	301/2061	14.8 (0.9)	20.3 (1.2)
Non-Black	5906/11310	42.2 (0.5)	64.3 (0.6)	123/11316	0.7 (0.1)	1.7 (0.2)	2419/11316	13.8 (0.3)	32.6 (0.6)	3749/11311	22.1 (0.4)	47.0 (0.6)	707/5383	12.6 (0.5)	17.2 (0.7)
**Hispanic/Latino Ethnicity**															
Hispanic	1579/3631	38.4 (0.8)	50.2 (1.0)	36/3631	0.6 (0.1)	1.5 (0.3)	588/3631	11.6 (0.6)	21.6 (0.8)	884/3630	18.4 (0.7)	31.2 (0.9)	175/1448	12.2 (0.9)	14.7 (1.1)
Non-Hispanic	6890/13609	38.8 (0.4)	64.7 (0.5)	260/13620	1.1 (0.1)	3.2 (0.2)	2731/13620	12.4 (0.3)	32.4 (0.6)	4507/13611	21.3 (0.4)	49.5 (0.6)	826/5948	13.4 (0.5)	18.9 (0.7)
**Education level**															
High school or less	5615/11875	37.4 (0.5)	59.8 (0.6)	200/11880	1.0 (0.1)	2.8 (0.2)	2187/11880	11.8 (0.3)	28.8 (0.6)	3658/11873	21.1 (0.4)	44.9 (0.6)	584/4631	12.5 (0.5)	17.3 (0.7)
More than high school	2332/4150	43.1 (0.8)	67.7 (0.9)	74/4154	0.8 (0.1)	2.8 (0.3)	934/4154	13.2 (0.6)	33.7 (1.0)	1398/4153	19.6 (0.6)	47.3 (1.0)	346/2286	14.0 (0.8)	19.0 (1.0)
**Treatment with antihypertensive drugs prior to trial baseline**															
Treated	7735/15707	38.9 (0.4)	61.6 (0.5)	280/15717	1.0 (0.1)	3.0 (0.2)	3067/15717	12.5 (0.3)	30.3 (0.5)	4944/15707	20.9 (0.3)	45.5 (0.5)	907/6629	13.4 (0.5)	18.2 (0.6)
Untreated	777/1622	36.7 (1.3)	58.7 (1.5)	16/1623	0.8 (0.2)	1.4 (0.4)	270/1623	9.6 (0.8)	24.9 (1.4)	475/1623	18.4 (1.0)	42.4 (1.7)	101/815	11.3 (1.2)	16.1 (1.6)
**Aspirin use (as of 1/1/1999)**															
Yes	3301/6404	40.8 (0.6)	65.2 (0.8)	101/6410	1.1 (0.1)	2.5 (0.3)	1292/6410	12.6 (0.4)	32.4 (0.9)	2033/6406	19.8 (0.5)	47.8 (0.9)	474/3356	13.6 (0.6)	18.7 (0.9)
No	5149/10759	37.5 (0.5)	59.4 (0.6)	193/10764	1.0 (0.1)	2.9 (0.2)	2024/10764	12.1 (0.3)	28.4 (0.6)	3347/10758	21.2 (0.4)	43.9 (0.6)	527/4013	12.9 (0.6)	17.5 (0.8)
**Women taking estrogen at trial baseline**															
Yes	745/1444	35.3 (1.3)	61.1 (1.5)	35/1444	1.0 (0.3)	3.5 (0.6)	414/1444	17.3 (1.0)	37.9 (1.6)	650/1443	29.6 (1.2)	55.5 (1.6)	---	---	---
No	3510/8270	31.4 (0.5)	54.2 (0.7)	161/8274	1.1 (0.1)	3.2 (0.3)	1541/8274	11.5 (0.4)	27.9 (0.7)	2827/8266	22.8 (0.5)	47.5 (0.7)	---	---	---
**HDL cholesterol <35 mg/dl (as of 1/1/1999)**															
Yes	1524/2784	46.0 (1.0)	68.7 (1.2)	35/2784	0.8 (0.2)	2.0 (0.4)	545/2784	12.9 (0.7)	33.2 (1.4)	836/2784	19.8 (0.8)	46.0 (1.4)	272/1909	14.4 (0.9)	17.9 (1.1)
No	6988/14545	37.3 (0.4)	60.0 (0.5)	261/14556	1.0 (0.1)	2.9 (0.2)	2792/14556	12.1 (0.3)	29.2 (0.5)	4583/14546	20.8 (0.4)	45.1 (0.6)	736/5535	12.8 (0.5)	18.0 (0.7)
**Cigarette smoking at trial baseline**															
Never smoker	3499/7649	34.8 (0.6)	56.6 (0.7)	138/7649	1.0 (0.1)	2.9 (0.3)	1490/7649	11.9 (0.4)	28.3 (0.7)	2497/7642	20.8 (0.5)	44.7 (0.7)	265/1895	13.2 (0.8)	18.0 (1.1)
Current smoker	1423/2841	40.5 (1.0)	64.7 (1.2)	46/2845	1.2 (0.2)	2.7 (0.5)	462/2845	11.3 (0.7)	28.4 (1.3)	817/2843	21.2 (0.8)	44.1 (1.4)	164/1389	11.9 (1.0)	16.6 (1.3)
Former Smoker	3590/6838	42.4 (0.6)	65.6 (0.8)	112/6845	0.9 (0.1)	2.7 (0.3)	1385/6845	13.1 (0.4)	32.1 (0.8)	2104/6844	20.2 (0.5)	46.3 (0.9)	578/4159	13.6 (0.6)	18.4 (0.8)
**Diabetes classification (as of 1/1/1999)**															
Diabetes	3279/7091	37.2 (0.6)	60.4 (0.8)	122/7091	1.0 (0.1)	3.1 (0.3)	1288/7091	12.2 (0.4)	29.9 (0.8)	2230/7086	21.9 (0.5)	48.1 (0.9)	360/2889	12.4 (0.7)	17.1 (1.0)
Non-diabetes	4612/8974	39.9 (0.5)	61.9 (0.6)	152/8981	1.0 (0.1)	2.6 (0.2)	1811/8981	12.3 (0.4)	29.7 (0.7)	2811/8976	19.6 (0.4)	43.6 (0.7)	583/4031	13.7 (0.6)	18.6 (0.8)
**History of Coronary Heart Disease (CHD) (as of 1/1/1999)**															
Yes	2446/4845	41.4 (0.8)	64.5 (0.9)	68/4848	1.0 (0.2)	2.3 (0.3)	970/4848	13.2 (0.5)	33.7 (1.1)	1532/4844	22.4 (0.6)	47.5 (1.0)	322/2593	12.4 (0.7)	17.3 (1.0)
No	6066/12484	37.7 (0.5)	60.2 (0.6)	228/12492	1.0 (0.1)	3.0 (0.2)	2367/12492	11.9 (0.3)	28.4 (0.6)	3887/12486	20.0 (0.4)	44.4 (0.6)	686/4851	13.6 (0.5)	18.4 (0.7)

**Atherosclerotic Cardiovascular Disease (ASCVD) at trial baseline**															
Yes	4700/9477	39.9 (0.5)	62.9 (0.6)	174/9482	1.1 (0.1)	3.1 (0.3)	1862/9482	13.0 (0.4)	31.2 (0.7)	2997/9478	21.8 (0.5)	46.4 (0.7)	564/4351	13.0 (0.6)	17.7 (0.8)
No	3812/7852	37.3 (0.6)	59.6 (0.7)	122/7858	0.9 (0.1)	2.4 (0.2)	1475/7858	11.4 (0.4)	28.1 (0.7)	2422/7852	19.3 (0.5)	43.7 (0.8)	444/3093	13.5 (0.7)	18.4 (0.9)
**History of myocardial infarction (MI) or stroke (as of 1/1/1999)**															
Yes	2247/4701	40.8 (0.8)	64.7 (1.0)	78/4705	1.1 (0.2)	2.9 (0.4)	859/4705	13.0 (0.5)	32.8 (1.1)	1519/4700	24.4 (0.7)	50.4 (1.1)	266/2388	11.2 (0.7)	16.8 (1.2)
No	6265/12628	38.0 (0.4)	60.3 (0.5)	218/12635	1.0 (0.1)	2.7 (0.2)	2478/12635	12.0 (0.3)	28.9 (0.5)	3900/12630	19.3 (0.4)	43.5 (0.6)	742/5056	14.1 (0.5)	18.6 (0.7)
**History of coronary artery bypass graft (CABG) (as of 1/1/1999)**															
Yes	1415/2676	43.1 (1.0)	69.3 (1.3)	38/2677	1.2 (0.2)	2.3 (0.5)	570/2677	14.0 (0.7)	37.0 (1.6)	850/2676	22.1 (0.9)	49.4 (1.5)	229/1721	13.1 (0.9)	18.5 (1.3)
No	7097/14653	37.9 (0.4)	60.1 (0.5)	258/14663	1.0 (0.1)	2.9 (0.2)	2767/14663	11.9 (0.3)	28.7 (0.5)	4569/14654	20.4 (0.4)	44.5 (0.6)	779/5723	13.2 (0.5)	17.9 (0.6)
**Other ASCVD at trial baseline**															
Yes	2274/4564	40.4 (0.8)	62.3 (0.9)	81/4567	1.0 (0.2)	3.1 (0.4)	908/4567	13.5 (0.5)	31.3 (1.0)	1460/4565	21.9 (0.7)	46.6 (1.0)	236/1934	12.3 (0.8)	16.2 (1.1)
No	6238/12765	38.1 (0.5)	61.0 (0.6)	215/12773	1.0 (0.1)	2.7 (0.2)	2429/12773	11.8 (0.3)	29.2 (0.6)	3959/12765	20.2 (0.4)	44.7 (0.6)	772/5510	13.5 (0.5)	18.5 (0.7)
**Major ST segment depression (as of 1/1/1999)**															
Yes	656/1389	38.5 (1.4)	61.7 (1.7)	29/1389	1.3 (0.3)	3.9 (0.8)	259/1389	12.6 (1.0)	29.7 (1.8)	400/1389	19.4 (1.1)	43.8 (1.9)	95/554	17.1 (1.8)	23.9 (2.4)
No	7834/15887	38.8 (0.4)	61.3 (0.5)	267/15898	1.0 (0.1)	2.7 (0.2)	3069/15898	12.2 (0.3)	29.7 (0.5)	5005/15888	20.8 (0.3)	45.3 (0.5)	911/6872	12.9 (0.4)	17.6 (0.6)
**Left ventricular hypertrophy (LVH) by Minnesota code (as of 1/1/1999)**															
Hard LVH	262/675	33.1 (2.0)	55.5 (2.8)	8/675	0.8 (0.4)	3.7 (1.5)	90/675	10.7 (1.4)	26.1 (3.0)	199/674	22.4 (1.8)	51.0 (3.3)	35/266	15.1 (2.4)	16.0 (2.6)
No/Soft LVH	7400/14765	39.0 (0.4)	62.4 (0.5)	261/14774	1.0 (0.1)	2.8 (0.2)	2955/14774	12.4 (0.3)	30.8 (0.5)	4680/14767	20.6 (0.4)	45.5 (0.6)	878/6471	13.0 (0.5)	18.2 (0.6)
**Lipid Lowering Trial (LLT) participant**															
Yes	2059/4217	38.6 (0.8)	59.3 (0.9)	69/4219	0.9 (0.2)	2.7 (0.3)	795/4219	11.9 (0.5)	28.4 (1.0)	1284/4218	19.3 (0.6)	42.5 (1.0)	232/1757	13.1 (0.9)	17.1 (1.1)
No	6453/13112	38.7 (0.4)	62.1 (0.5)	227/13121	1.0 (0.1)	2.8 (0.2)	2542/13121	12.4 (0.3)	30.2 (0.6)	4135/13112	21.1 (0.4)	46.1 (0.6)	776/5687	13.2 (0.5)	18.3 (0.7)
**Obesity (BMI ≥ 30 kg/m²) at trial baseline**															
Yes	3389/6874	36.7 (0.6)	61.5 (0.7)	158/6881	1.2 (0.1)	3.8 (0.3)	1498/6881	13.2 (0.4)	33.0 (0.8)	2327/6876	21.3 (0.5)	48.1 (0.8)	361/2570	13.5 (0.7)	18.3 (1.0)
No	5123/10455	40.0 (0.5)	61.2 (0.6)	138/10459	0.8 (0.1)	2.1 (0.2)	1839/10459	11.6 (0.3)	27.4 (0.6)	3092/10454	20.2 (0.4)	43.1 (0.7)	647/4874	13.1 (0.5)	17.8 (0.7)

n= number of patients experiencing event; N= number of patients eligible to be studied for event; SE= Standard Error

Cancer diagnoses excluded non-melanoma skin cancers, and included patients from inpatient, outpatient, and carrier data.

†18-year time to event includes events occurring up to, but not including year 19 (e.g. throughout the year 2017)

Table S3b: Cumulative Incidence (%) of 5 outcomes from any diagnosis in Medicare Inpatient Data on ≥2 times with 30 days apart by the 3 study drugs and other factors (1999 to 2017)

Demographic	Cancer			Angioedema			Insomnia			Depression			Erectile Dysfunction		
	**Events/ Total (n/N)**	**6-Year % (SE)**	**18-Year % (SE)**	**Events/ Total (n/N)**	**6-Year % (SE)**	**18-Year % (SE)**	**Events/ Total (n/N)**	**6-Year % (SE)**	**18-Year % (SE)**	**Events/ Total n/N**	**6-Year % (SE)**	**18-Year % (SE)**	**Events/ Total (n/N)**	**6-Year % (SE)**	**18-Year % (SE)**
All Patients	1208/17332	4.7 (0.2)	9.9 (0.3)	65/17340	0.2 (0)	0.6 (0.1)	1775/17340	7.1 (0.2)	15.2 (0.4)	3536/17330	14.4 (0.3)	29.5 (0.5)	605/7444	8 (0.3)	10.4 (0.4)
**Randomized group**															
Chlorthalidone	529/7993	4.5 (0.2)	9.4 (0.4)	21/7996	0.2 (0.0)	0.4 (0.1)	829/7996	7.2 (0.3)	15.4 (0.5)	1619/7992	14.3 (0.4)	29.1 (0.7)	278/3425	7.8 (0.5)	10.6 (0.7)
Amlodipine	315/4680	4.6 (0.3)	9.6 (0.6)	19/4682	0.2 (0.1)	0.6 (0.1)	483/4682	7.4 (0.4)	15.1 (0.7)	967/4680	14.5 (0.5)	29.7 (0.9)	159/1969	8.3 (0.7)	9.8 (0.8)
Lisinopril	364/4659	5.2 (0.3)	11.0 (0.6)	25/4662	0.3 (0.1)	0.9 (0.2)	463/4662	6.7 (0.4)	14.9 (0.7)	950/4658	14.5 (0.5)	29.8 (0.9)	168/2050	8.0 (0.6)	10.7 (0.9)
**Age group (as of 1/1/1999)**															
Age <70	455/6035	4.3 (0.3)	9.7 (0.4)	24/6037	0.1 (0.0)	0.6 (0.1)	796/6037	8.0 (0.4)	17.3 (0.6)	1323/6035	13.6 (0.5)	28.4 (0.7)	327/2749	10.9 (0.6)	14.0 (0.7)
Age 70-79	617/8489	4.9 (0.2)	10.1 (0.4)	33/8494	0.3 (0.1)	0.5 (0.1)	813/8494	6.8 (0.3)	14.1 (0.5)	1733/8488	14.4 (0.4)	29.9 (0.7)	253/3669	6.9 (0.4)	8.7 (0.6)
Age 80+	136/2808	5.0 (0.5)	8.5 (1.0)	8/2809	0.3 (0.1)	0.5 (0.2)	166/2809	6.1 (0.5)	11.1 (1.1)	480/2807	16.8 (0.8)	29.5 (1.5)	25/1026	3.2 (0.6)	3.2 (0.6)
**Gender**															
Male	643/7439	6.0 (0.3)	12.6 (0.5)	17/7444	0.2 (0.1)	0.3 (0.1)	716/7444	7.1 (0.3)	14.8 (0.6)	1181/7443	11.0 (0.4)	24.9 (0.7)	---	---	---
Female	565/9893	3.8 (0.2)	8.0 (0.3)	48/9896	0.2 (0.1)	0.7 (0.1)	1059/9896	7.1 (0.3)	15.5 (0.5)	2355/9887	16.9 (0.4)	32.7 (0.6)	---	---	---
**Race/Ethnicity**															
Black	494/6020	5.9 (0.3)	11.8 (0.5)	45/6024	0.4 (0.1)	1.2 (0.2)	471/6024	5.2 (0.3)	12.1 (0.6)	1013/6019	11.4 (0.4)	25.5 (0.8)	201/2061	10.0 (0.7)	12.9 (0.9)
Non-Black	714/11312	4.1 (0.2)	8.8 (0.3)	20/11316	0.1 (0.0)	0.3 (0.1)	1304/11316	8.1 (0.3)	16.9 (0.5)	2523/11311	16.0 (0.4)	31.6 (0.6)	404/5383	7.2 (0.4)	9.5 (0.5)
**Hispanic/Latino Ethnicity**															
Hispanic	145/3631	2.9 (0.3)	5.1 (0.4)	10/3631	0.1 (0.1)	0.4 (0.1)	294/3631	6.1 (0.4)	10.6 (0.6)	547/3630	11.9 (0.6)	19.2 (0.8)	94/1448	6.4 (0.7)	7.6 (0.8)
Non-Hispanic	1056/13612	5.2 (0.2)	11.3 (0.4)	55/13620	0.2 (0.0)	0.6 (0.1)	1475/13620	7.4 (0.2)	16.7 (0.5)	2971/13611	15.1 (0.3)	32.6 (0.6)	505/5948	8.3 (0.4)	11.1 (0.5)
**Education level**															
High school or less	824/11875	4.9 (0.2)	10.0 (0.4)	48/11880	0.2 (0.0)	0.6 (0.1)	1126/11880	6.6 (0.2)	14.3 (0.4)	2379/11873	14.5 (0.3)	29.3 (0.6)	349/4631	7.6 (0.4)	9.8 (0.5)
More than high school	298/4151	4.4 (0.3)	9.6 (0.6)	14/4154	0.2 (0.1)	0.5 (0.1)	531/4154	8.4 (0.5)	18.3 (0.8)	935/4153	14.6 (0.6)	31.2 (0.9)	212/2286	8.6 (0.6)	11.3 (0.8)
**Treatment with antihypertensive drugs prior to trial baseline**															
Treated	1105/15709	4.8 (0.2)	10.0 (0.3)	62/15717	0.2 (0.0)	0.6 (0.1)	1629/15717	7.3 (0.2)	15.4 (0.4)	3230/15707	14.5 (0.3)	29.8 (0.5)	544/6629	8.1 (0.4)	10.5 (0.5)
Untreated	103/1623	3.9 (0.5)	8.8 (0.9)	3/1623	0.1 (0.1)	0.3 (0.2)	146/1623	5.5 (0.6)	13.3 (1.1)	306/1623	13.2 (0.9)	26.7 (1.4)	61/815	6.8 (0.9)	9.8 (1.3)
**Aspirin use (as of 1/1/1999)**															
Yes	469/6406	4.9 (0.3)	10.5 (0.5)	22/6410	0.2 (0.1)	0.5 (0.1)	667/6410	7.3 (0.3)	16.1 (0.7)	1338/6406	14.0 (0.5)	31.8 (0.8)	285/3356	8.2 (0.5)	10.9 (0.7)
No	729/10760	4.6 (0.2)	9.5 (0.4)	43/10764	0.2 (0.0)	0.6 (0.1)	1098/10764	7.0 (0.3)	14.8 (0.5)	2171/10758	14.7 (0.4)	28.3 (0.6)	315/4013	7.9 (0.5)	10.1 (0.6)
**Women taking estrogen at trial baseline**															
Yes	91/1444	3.5 (0.5)	8.2 (0.9)	8/1444	0.1 (0.1)	0.9 (0.3)	249/1444	10.7 (0.8)	22.9 (1.4)	482/1443	23.5 (1.2)	41.2 (1.6)	---	---	---
No	466/8271	3.8 (0.2)	8.0 (0.4)	39/8274	0.3 (0.1)	0.7 (0.1)	791/8274	6.5 (0.3)	13.9 (0.5)	1832/8266	15.7 (0.4)	31.0 (0.7)	---	---	---
**HDL cholesterol <35 mg/dl (as of 1/1/1999)**															
Yes	226/2784	5.3 (0.5)	12.1 (0.8)	6/2784	0.1 (0.1)	0.3 (0.1)	295/2784	7.5 (0.5)	16.6 (1.0)	539/2784	13.6 (0.7)	28.7 (1.2)	163/1909	8.9 (0.7)	10.6 (0.9)
No	982/14548	4.6 (0.2)	9.5 (0.3)	59/14556	0.2 (0.0)	0.6 (0.1)	1480/14556	7.0 (0.2)	15.0 (0.4)	2997/14546	14.6 (0.3)	29.6 (0.5)	442/5535	7.7 (0.4)	10.4 (0.5)
**Cigarette smoking at trial baseline**															
Never smoker	419/7649	3.6 (0.2)	7.6 (0.4)	31/7649	0.2 (0.1)	0.6 (0.1)	807/7649	6.9 (0.3)	15.0 (0.5)	1638/7642	14.9 (0.4)	29.2 (0.7)	153/1895	7.6 (0.6)	10.1 (0.8)
Current smoker	281/2842	7.3 (0.5)	14.8 (0.9)	12/2845	0.3 (0.1)	0.7 (0.2)	244/2845	6.2 (0.5)	14.3 (1.0)	533/2843	14.4 (0.7)	29.1 (1.3)	107/1389	7.9 (0.8)	10.2 (1.0)
Former Smoker	508/6840	5.0 (0.3)	10.7 (0.5)	22/6845	0.2 (0.1)	0.5 (0.1)	724/6845	7.7 (0.3)	15.8 (0.6)	1364/6844	13.9 (0.4)	30.1 (0.8)	344/4159	8.2 (0.5)	10.6 (0.6)
**Diabetes classification (as of 1/1/1999)**															
Diabetes	473/7091	4.7 (0.3)	10.1 (0.5)	25/7091	0.2 (0.1)	0.5 (0.1)	672/7091	6.9 (0.3)	15.0 (0.6)	1453/7086	15.1 (0.5)	31.4 (0.8)	216/2889	7.4 (0.5)	10.1 (0.7)
Non-diabetes	648/8976	4.7 (0.2)	9.7 (0.4)	38/8981	0.2 (0.1)	0.6 (0.1)	977/8981	7.3 (0.3)	15.3 (0.5)	1838/8976	13.8 (0.4)	28.4 (0.6)	348/4031	8.3 (0.5)	10.7 (0.6)
**History of Coronary Heart Disease (CHD) (as of 1/1/1999)**															
Yes	325/4845	4.7 (0.3)	10.0 (0.6)	16/4848	0.3 (0.1)	0.4 (0.1)	487/4848	7.2 (0.4)	16.0 (0.8)	970/4844	15.1 (0.6)	30.6 (1.0)	184/2593	7.1 (0.5)	9.4 (0.7)
No	883/12487	4.7 (0.2)	9.8 (0.3)	49/12492	0.2 (0.0)	0.6 (0.1)	1288/12492	7.1 (0.2)	15.0 (0.4)	2566/12486	14.2 (0.3)	29.1 (0.5)	421/4851	8.4 (0.4)	10.9 (0.5)
**Atherosclerotic Cardiovascular Disease (ASCVD) at trial baseline**															
Yes	650/9479	4.8 (0.2)	10.0 (0.4)	38/9482	0.2 (0.1)	0.6 (0.1)	991/9482	7.4 (0.3)	15.9 (0.5)	1969/9478	15.2 (0.4)	30.5 (0.7)	315/4351	7.3 (0.4)	9.4 (0.5)
No	558/7853	4.6 (0.2)	9.7 (0.4)	27/7858	0.2 (0.1)	0.5 (0.1)	784/7858	6.7 (0.3)	14.5 (0.5)	1567/7852	13.4 (0.4)	28.3 (0.7)	290/3093	8.9 (0.5)	11.8 (0.7)
**History of myocardial infarction (MI) or stroke (as of 1/1/1999)**															

Yes	316/4702	5.2 (0.4)	10.5 (0.6)	14/4705	0.2 (0.1)	0.5 (0.1)	441/4705	7.1 (0.4)	15.8 (0.8)	1003/4700	16.9 (0.6)	33.6 (1.0)	146/2388	6.4 (0.6)	8.3 (0.7)
No	892/12630	4.6 (0.2)	9.7 (0.3)	51/12635	0.2 (0.0)	0.6 (0.1)	1334/12635	7.1 (0.2)	15.1 (0.4)	2533/12630	13.5 (0.3)	28.2 (0.5)	459/5056	8.7 (0.4)	11.3 (0.5)
**History of coronary artery bypass graft (CABG) (as of 1/1/1999)**															
Yes	191/2676	4.5 (0.4)	11.5 (0.9)	9/2677	0.3 (0.1)	0.4 (0.1)	290/2677	7.9 (0.6)	17.1 (1.1)	537/2676	14.7 (0.7)	31.3 (1.3)	126/1721	7.1 (0.7)	9.7 (0.9)
No	1017/14656	4.8 (0.2)	9.6 (0.3)	56/14663	0.2 (0.0)	0.6 (0.1)	1485/14663	7.0 (0.2)	14.9 (0.4)	2999/14654	14.4 (0.3)	29.2 (0.5)	479/5723	8.2 (0.4)	10.7 (0.5)
**Other ASCVD at trial baseline**															
Yes	306/4565	4.6 (0.3)	9.7 (0.6)	18/4567	0.2 (0.1)	0.6 (0.2)	480/4567	7.4 (0.4)	16.1 (0.8)	938/4565	14.8 (0.6)	30.0 (0.9)	134/1934	6.8 (0.6)	9.2 (0.8)
No	902/12767	4.7 (0.2)	9.9 (0.3)	47/12773	0.2 (0.0)	0.6 (0.1)	1295/12773	7.0 (0.2)	14.9 (0.4)	2598/12765	14.3 (0.3)	29.3 (0.5)	471/5510	8.4 (0.4)	10.8 (0.5)
**Major ST segment depression (as of 1/1/1999)**															
Yes	111/1389	5.6 (0.7)	12.0 (1.2)	6/1389	0.2 (0.1)	0.8 (0.3)	148/1389	7.5 (0.8)	16.6 (1.4)	273/1389	14.7 (1.0)	29.1 (1.7)	46/554	8.4 (1.3)	10.8 (1.6)
No	1093/15890	4.6 (0.2)	9.7 (0.3)	59/15898	0.2 (0.0)	0.6 (0.1)	1624/15898	7.1 (0.2)	15.1 (0.4)	3251/15888	14.4 (0.3)	29.5 (0.5)	559/6872	8.0 (0.3)	10.4 (0.4)
**Left ventricular hypertrophy (LVH) by Minnesota code (as of 1/1/1999)**															
Hard LVH	46/675	5.9 (1.0)	10.2 (1.6)	1/675	0.2 (0.2)	0.2 (0.2)	47/675	5.7 (1.0)	13.1 (2.2)	129/674	15.3 (1.5)	33.6 (3.1)	22/266	9.9 (2.0)	9.9 (2.0)
No/Soft LVH	1029/14767	4.6 (0.2)	9.9 (0.3)	60/14774	0.2 (0.0)	0.6 (0.1)	1573/14774	7.3 (0.2)	15.8 (0.4)	3067/14767	14.4 (0.3)	29.9 (0.5)	520/6471	7.8 (0.4)	10.4 (0.5)
**Lipid Lowering Trial (LLT) participant**															
Yes	273/4217	4.4 (0.3)	8.9 (0.5)	15/4219	0.2 (0.1)	0.5 (0.1)	413/4219	6.8 (0.4)	13.9 (0.7)	835/4218	13.5 (0.6)	27.4 (0.9)	135/1757	7.8 (0.7)	9.4 (0.8)
No	935/13115	4.8 (0.2)	10.2 (0.3)	50/13121	0.2 (0.0)	0.6 (0.1)	1362/13121	7.2 (0.2)	15.7 (0.4)	2701/13112	14.7 (0.3)	30.2 (0.6)	470/5687	8.0 (0.4)	10.8 (0.5)
**Obesity (BMI ≥ 30 kg/m^2) at trial baseline**															
Yes	484/6875	4.4 (0.3)	9.9 (0.5)	32/6881	0.2 (0.1)	0.7 (0.1)	808/6881	7.8 (0.3)	17.1 (0.6)	1560/6876	15.4 (0.5)	32.4 (0.8)	220/2570	8.4 (0.6)	10.5 (0.7)
No	724/10457	5.0 (0.2)	9.8 (0.4)	33/10459	0.2 (0.0)	0.5 (0.1)	967/10459	6.6 (0.3)	13.9 (0.5)	1976/10454	13.7 (0.4)	27.4 (0.6)	385/4874	7.7 (0.4)	10.4 (0.5)

n= number of patients experiencing event; N= number of patients eligible to be studied for event; SE= Standard Error
Cancer diagnoses excluded non-melanoma skin cancers, and included patients from inpatient data only.
†18-year time to event includes events occurring up to, but not including year 19 (e.g. throughout the year 2017)

Table S3c: Cumulative Incidence (%) of 5 outcomes from any diagnosis in Medicare Inpatient, Outpatient and Physician Carries claims on ≥2 times with 30 days apart by the 3 study drugs and other factors (1999 to 2017)

	Cancer			Angioedema			Insomnia			Depression			Erectile Dysfunction		
**Demographic**	**Events/ Total (n/N)**	**6-Year % (SE)**	**18-Year % (SE)**	**Events/ Total (n/N)**	**6-Year % (SE)**	**18-Year % (SE)**	**Events/ Total (n/N)**	**6-Year % (SE)**	**18-Year % (SE)**	**Events/ Total n/N**	**6-Year % (SE)**	**18-Year % (SE)**	**Events/ Total (n/N)**	**6-Year % (SE)**	**18-Year % (SE)**
All Patients	5958/17329	29 (0.4)	42.8 (0.5)	65/17340	0.2 (0)	0.6 (0.1)	1762/17340	7.1 (0.2)	15.1 (0.4)	3506/17330	14.3 (0.3)	29.2 (0.5)	534/7444	7 (0.3)	9.2 (0.4)
**Randomized group**															
Chlorthalidone	2703/7992	28.7 (0.5)	42.5 (0.7)	21/7996	0.2 (0.0)	0.4 (0.1)	822/7996	7.2 (0.3)	15.3 (0.5)	1605/7992	14.2 (0.4)	28.9 (0.7)	249/3425	6.9 (0.5)	9.5 (0.6)
Amlodipine	1586/4678	28.9 (0.7)	41.5 (0.9)	19/4682	0.2 (0.1)	0.6 (0.1)	482/4682	7.4 (0.4)	15.1 (0.7)	960/4680	14.3 (0.5)	29.6 (0.9)	138/1969	7.1 (0.6)	8.6 (0.7)
Lisinopril	1669/4659	29.6 (0.7)	44.7 (0.9)	25/4662	0.3 (0.1)	0.9 (0.2)	458/4662	6.6 (0.4)	14.7 (0.7)	941/4658	14.3 (0.5)	29.5 (0.9)	147/2050	6.9 (0.6)	9.3 (0.8)
**Age group (as of 1/1/1999)**															
Age <70	2181/6035	26.7 (0.6)	42.6 (0.7)	24/6037	0.1 (0.0)	0.6 (0.1)	787/6037	7.9 (0.4)	17.1 (0.6)	1311/6035	13.4 (0.5)	28.1 (0.7)	286/2749	9.5 (0.6)	12.2 (0.7)
Age 70-79	3005/8487	30.5 (0.5)	43.1 (0.7)	33/8494	0.3 (0.1)	0.5 (0.1)	809/8494	6.8 (0.3)	14.0 (0.5)	1720/8488	14.2 (0.4)	29.7 (0.7)	226/3669	6.0 (0.4)	7.9 (0.5)
Age 80+	772/2807	29.3 (1.0)	37.2 (1.3)	8/2809	0.3 (0.1)	0.5 (0.2)	166/2809	6.1 (0.5)	11.1 (1.1)	475/2807	16.6 (0.8)	29.1 (1.5)	22/1026	2.6 (0.6)	3.0 (0.7)
**Gender**															
Male	3132/7437	38.0 (0.6)	52.4 (0.7)	17/7444	0.2 (0.1)	0.3 (0.1)	712/7444	7.1 (0.3)	14.7 (0.6)	1163/7443	10.8 (0.4)	24.6 (0.7)	---	---	---
Female	2826/9892	22.4 (0.4)	36.0 (0.6)	48/9896	0.2 (0.1)	0.7 (0.1)	1050/9896	7.1 (0.3)	15.3 (0.5)	2343/9887	16.8 (0.4)	32.5 (0.6)	---	---	---
**Race/Ethnicity**															
Black	1750/6019	23.2 (0.6)	37.3 (0.8)	45/6024	0.4 (0.1)	1.2 (0.2)	467/6024	5.2 (0.3)	11.9 (0.6)	1007/6019	11.3 (0.4)	25.4 (0.8)	170/2061	8.3 (0.7)	11.0 (0.9)
Non-Black	4208/11310	32.1 (0.5)	45.7 (0.6)	20/11316	0.1 (0.0)	0.3 (0.1)	1295/11316	8.1 (0.3)	16.7 (0.5)	2499/11311	15.9 (0.4)	31.3 (0.6)	364/5383	6.5 (0.4)	8.6 (0.5)
**Hispanic/Latino Ethnicity**															
Hispanic	996/3631	25.3 (0.8)	31.5 (0.9)	10/3631	0.1 (0.1)	0.4 (0.1)	294/3631	6.1 (0.4)	10.6 (0.6)	544/3630	11.8 (0.6)	19.1 (0.8)	90/1448	6.2 (0.7)	7.2 (0.7)
Non-Hispanic	4927/13609	30.0 (0.4)	46.1 (0.5)	55/13620	0.2 (0.0)	0.6 (0.1)	1462/13620	7.4 (0.2)	16.6 (0.4)	2944/13611	15.0 (0.3)	32.4 (0.6)	438/5948	7.1 (0.4)	9.7 (0.5)
**Education level**															
High school or less	3829/11875	27.3 (0.4)	40.7 (0.6)	48/11880	0.2 (0.0)	0.6 (0.1)	1117/11880	6.5 (0.2)	14.1 (0.4)	2355/11873	14.4 (0.3)	29.1 (0.6)	304/4631	6.5 (0.4)	8.7 (0.5)
More than high school	1750/4150	35.1 (0.8)	50.3 (0.9)	14/4154	0.2 (0.1)	0.5 (0.1)	527/4154	8.3 (0.5)	18.2 (0.8)	929/4153	14.4 (0.6)	31.0 (0.9)	189/2286	7.8 (0.6)	9.9 (0.7)
**Treatment with antihypertensive drugs prior to trial baseline**															
Treated	5425/15707	29.2 (0.4)	43.1 (0.5)	62/15717	0.2 (0.0)	0.6 (0.1)	1617/15717	7.3 (0.2)	15.3 (0.4)	3203/15707	14.4 (0.3)	29.5 (0.5)	484/6629	7.1 (0.3)	9.4 (0.4)
Untreated	533/1622	26.8 (1.2)	40.1 (1.5)	3/1623	0.1 (0.1)	0.3 (0.2)	145/1623	5.5 (0.6)	13.2 (1.1)	303/1623	13.1 (0.9)	26.5 (1.4)	50/815	5.7 (0.9)	7.7 (1.1)
**Aspirin use (as of 1/1/1999)**															
Yes	2342/6404	31.2 (0.6)	45.7 (0.8)	22/6410	0.2 (0.1)	0.5 (0.1)	661/6410	7.3 (0.3)	15.9 (0.7)	1329/6406	13.9 (0.5)	31.6 (0.8)	264/3356	7.5 (0.5)	10.1 (0.6)
No	3576/10759	27.8 (0.5)	41.3 (0.6)	43/10764	0.2 (0.0)	0.6 (0.1)	1091/10764	7.0 (0.3)	14.7 (0.4)	2150/10758	14.5 (0.4)	28.1 (0.6)	265/4013	6.6 (0.4)	8.4 (0.5)
**Women taking estrogen at trial baseline**															
Yes	512/1444	26.0 (1.2)	41.6 (1.5)	8/1444	0.1 (0.1)	0.9 (0.3)	244/1444	10.5 (0.8)	22.4 (1.4)	480/1443	23.4 (1.2)	41.0 (1.6)	---	---	---
No	2278/8270	21.9 (0.5)	35.0 (0.6)	39/8274	0.3 (0.1)	0.7 (0.1)	787/8274	6.5 (0.3)	13.8 (0.5)	1823/8266	15.6 (0.4)	30.9 (0.7)	---	---	---
**HDL cholesterol <35 mg/dl (as of 1/1/1999)**															
Yes	1105/2784	35.6 (1.0)	50.0 (1.2)	6/2784	0.1 (0.1)	0.3 (0.1)	292/2784	7.5 (0.5)	16.4 (1.0)	532/2784	13.6 (0.7)	28.4 (1.2)	145/1909	7.9 (0.7)	9.5 (0.8)
No	4853/14545	27.8 (0.4)	41.5 (0.5)	59/14556	0.2 (0.0)	0.6 (0.1)	1470/14556	7.0 (0.2)	14.8 (0.4)	2974/14546	14.4 (0.3)	29.4 (0.5)	389/5535	6.7 (0.4)	9.1 (0.5)
**Cigarette smoking at trial baseline**															
Never smoker	2369/7649	25.4 (0.5)	37.8 (0.7)	31/7649	0.2 (0.1)	0.6 (0.1)	802/7649	6.9 (0.3)	14.9 (0.5)	1626/7642	14.7 (0.4)	29.0 (0.7)	134/1895	6.6 (0.6)	8.8 (0.8)
Current smoker	992/2841	30.0 (0.9)	45.1 (1.2)	12/2845	0.3 (0.1)	0.7 (0.2)	240/2845	6.1 (0.5)	14.1 (1.0)	529/2843	14.2 (0.7)	29.0 (1.3)	91/1389	6.6 (0.7)	8.7 (0.9)
Former Smoker	2597/6838	32.7 (0.6)	47.7 (0.8)	22/6845	0.2 (0.1)	0.5 (0.1)	720/6845	7.7 (0.3)	15.7 (0.6)	1350/6844	13.8 (0.4)	29.8 (0.8)	308/4159	7.3 (0.4)	9.5 (0.6)
**Diabetes classification (as of 1/1/1999)**															
Diabetes	2224/7091	27.2 (0.6)	40.7 (0.8)	25/7091	0.2 (0.1)	0.5 (0.1)	670/7091	6.9 (0.3)	14.9 (0.6)	1439/7086	15.0 (0.5)	31.1 (0.8)	188/2889	6.3 (0.5)	8.8 (0.7)
Non-diabetes	3304/8974	30.5 (0.5)	44.2 (0.6)	38/8981	0.2 (0.1)	0.6 (0.1)	968/8981	7.3 (0.3)	15.2 (0.5)	1823/8976	13.7 (0.4)	28.3 (0.6)	307/4031	7.3 (0.4)	9.4 (0.5)
**History of Coronary Heart Disease (CHD) (as of 1/1/1999)**															
Yes	1719/4845	31.2 (0.7)	45.2 (0.9)	16/4848	0.3 (0.1)	0.4 (0.1)	484/4848	7.2 (0.4)	15.8 (0.8)	963/4844	15.0 (0.6)	30.4 (1.0)	164/2593	6.2 (0.5)	8.5 (0.7)
No	4239/12484	28.2 (0.4)	41.9 (0.5)	49/12492	0.2 (0.0)	0.6 (0.1)	1278/12492	7.1 (0.2)	14.8 (0.4)	2543/12486	14.0 (0.3)	28.9 (0.5)	370/4851	7.4 (0.4)	9.6 (0.5)
**Atherosclerotic Cardiovascular Disease (ASCVD) at trial baseline**															
Yes	3273/9477	29.8 (0.5)	43.6 (0.6)	38/9482	0.2 (0.1)	0.6 (0.1)	984/9482	7.4 (0.3)	15.7 (0.5)	1953/9478	15.1 (0.4)	30.3 (0.7)	282/4351	6.4 (0.4)	8.4 (0.5)
No	2685/7852	28.0 (0.5)	41.8 (0.7)	27/7858	0.2 (0.1)	0.5 (0.1)	778/7858	6.7 (0.3)	14.4 (0.5)	1553/7852	13.3 (0.4)	28.1 (0.7)	252/3093	7.7 (0.5)	10.2 (0.6)
**History of myocardial infarction (MI) or stroke (as of 1/1/1999)**															
Yes	1548/4701	30.1 (0.7)	44.5 (1.0)	14/4705	0.2 (0.1)	0.5 (0.1)	439/4705	7.1 (0.4)	15.6 (0.8)	995/4700	16.8 (0.6)	33.3 (1.0)	136/2388	5.8 (0.5)	8.0 (0.7)

No	4410/12628	28.6 (0.4)	42.3 (0.5)	51/12635	0.2 (0.0)	0.6 (0.1)	1323/12635	7.1 (0.2)	14.9 (0.4)	2511/12630	13.4 (0.3)	28.0 (0.5)	398/5056	7.5 (0.4)	9.7 (0.5)
**History of coronary artery bypass graft (CABG) (as of 1/1/1999)**															
Yes	1010/2676	32.9 (1.0)	48.9 (1.3)	9/2677	0.3 (0.1)	0.4 (0.1)	288/2677	7.9 (0.6)	16.9 (1.1)	530/2676	14.6 (0.7)	30.8 (1.3)	113/1721	6.3 (0.6)	8.9 (0.9)
No	4948/14653	28.3 (0.4)	41.8 (0.5)	56/14663	0.2 (0.0)	0.6 (0.1)	1474/14663	6.9 (0.2)	14.8 (0.4)	2976/14654	14.2 (0.3)	29.0 (0.5)	421/5723	7.2 (0.4)	9.3 (0.5)
**Other ASCVD at trial baseline**															
Yes	1598/4564	30.3 (0.7)	43.4 (0.9)	18/4567	0.2 (0.1)	0.6 (0.2)	478/4567	7.4 (0.4)	16.0 (0.8)	931/4565	14.7 (0.6)	29.8 (0.9)	123/1934	6.1 (0.6)	8.5 (0.8)
No	4360/12765	28.5 (0.4)	42.5 (0.5)	47/12773	0.2 (0.0)	0.6 (0.1)	1284/12773	7.0 (0.2)	14.8 (0.4)	2575/12765	14.1 (0.3)	29.0 (0.5)	411/5510	7.3 (0.4)	9.4 (0.5)
**Major ST segment depression (as of 1/1/1999)**															
Yes	444/1389	28.0 (1.3)	41.4 (1.7)	6/1389	0.2 (0.1)	0.8 (0.3)	145/1389	7.5 (0.8)	16.1 (1.4)	271/1389	14.5 (1.0)	28.9 (1.7)	39/554	7.1 (1.2)	8.7 (1.4)
No	5499/15887	29.1 (0.4)	42.9 (0.5)	59/15898	0.2 (0.0)	0.6 (0.1)	1614/15898	7.1 (0.2)	15.0 (0.4)	3223/15888	14.3 (0.3)	29.2 (0.5)	495/6872	7.0 (0.3)	9.3 (0.4)
**Left ventricular hypertrophy (LVH) by Minnesota code (as of 1/1/1999)**															
Hard LVH	175/675	23.9 (1.8)	36.1 (2.6)	1/675	0.2 (0.2)	0.2 (0.2)	47/675	5.7 (1.0)	13.1 (2.2)	127/674	14.9 (1.5)	33.2 (3.1)	19/266	8.7 (1.9)	8.7 (1.9)
No/Soft LVH	5209/14765	29.4 (0.4)	43.8 (0.5)	60/14774	0.2 (0.0)	0.6 (0.1)	1561/14774	7.2 (0.2)	15.6 (0.4)	3043/14767	14.3 (0.3)	29.6 (0.5)	460/6471	6.8 (0.3)	9.2 (0.4)
**Lipid Lowering Trial (LLT) participant**															
Yes	1458/4217	29.0 (0.7)	42.0 (0.9)	15/4219	0.2 (0.1)	0.5 (0.1)	407/4219	6.8 (0.4)	13.7 (0.7)	825/4218	13.3 (0.6)	27.0 (0.9)	116/1757	6.6 (0.6)	8.0 (0.7)
No	4500/13112	29.0 (0.4)	43.1 (0.5)	50/13121	0.2 (0.0)	0.6 (0.1)	1355/13121	7.2 (0.2)	15.6 (0.4)	2681/13112	14.6 (0.3)	30.0 (0.6)	418/5687	7.1 (0.4)	9.6 (0.5)
**Obesity (BMI ≥ 30 kg/m²) at trial baseline**															
Yes	2371/6874	27.9 (0.6)	42.8 (0.7)	32/6881	0.2 (0.1)	0.7 (0.1)	804/6881	7.8 (0.3)	17.0 (0.6)	1549/6876	15.2 (0.5)	32.3 (0.8)	199/2570	7.5 (0.6)	9.5 (0.7)
No	3587/10455	29.8 (0.5)	42.7 (0.6)	33/10459	0.2 (0.0)	0.5 (0.1)	958/10459	6.6 (0.3)	13.7 (0.5)	1957/10454	13.6 (0.4)	27.1 (0.6)	335/4874	6.7 (0.4)	9.0 (0.5)

n= number of patients experiencing event; N= number of patients eligible to be studied for event; SE= Standard Error

Cancer diagnoses excluded non-melanoma skin cancers, and included patients from inpatient, outpatient, and carrier data.

†18-year time to event includes events occurring up to, but not including year 19 (e.g. throughout the year 2017)

Table S3d: Cumulative Incidence (%) of 5 outcomes from primary diagnosis in Medicare Inpatient Data on ≥2 times with 30 days apart by the 3 study drugs and other factors (1999 to 2017)

Demographic	Cancer			Angioedema			Insomnia			Depression			Erectile Dysfunction		
	**Events/ Total (n/N)**	**6-Year % (SE)**	**18-Year % (SE)**	**Events/ Total (n/N)**	**6-Year % (SE)**	**18-Year % (SE)**	**Events/ Total (n/N)**	**6-Year % (SE)**	**18-Year % (SE)**	**Events/ Total n/N**	**6-Year % (SE)**	**18-Year % (SE)**	**Events/ Total (n/N)**	**6-Year % (SE)**	**18-Year % (SE)**
All Patients	370/17332	1.6 (0.1)	2.9 (0.2)	54/17340	0.2 (0)	0.5 (0.1)	706/17340	3.1 (0.1)	5.7 (0.2)	1729/17330	6.9 (0.2)	14.7 (0.4)	210/7444	2.7 (0.2)	3.6 (0.3)
**Randomized group**															
Chlorthalidone	154/7993	1.5 (0.1)	2.6 (0.2)	19/7996	0.1 (0.0)	0.4 (0.1)	320/7996	3.0 (0.2)	5.5 (0.3)	796/7992	6.9 (0.3)	14.5 (0.5)	94/3425	2.6 (0.3)	3.6 (0.4)
Amlodipine	108/4680	1.7 (0.2)	3.2 (0.3)	15/4682	0.2 (0.1)	0.5 (0.1)	205/4682	3.3 (0.3)	6.2 (0.5)	455/4680	6.7 (0.4)	14.5 (0.7)	56/1969	2.8 (0.4)	3.7 (0.5)
Lisinopril	108/4659	1.7 (0.2)	3.1 (0.3)	20/4662	0.2 (0.1)	0.7 (0.2)	181/4662	2.9 (0.3)	5.6 (0.4)	478/4658	7.2 (0.4)	15.3 (0.7)	60/2050	2.9 (0.4)	3.5 (0.5)
**Age group (as of 1/1/1999)**															
Age <70	149/6035	1.6 (0.2)	3.1 (0.3)	20/6037	0.1 (0.0)	0.5 (0.1)	329/6037	3.7 (0.3)	6.7 (0.4)	677/6035	6.7 (0.3)	14.7 (0.6)	118/2749	3.8 (0.4)	5.1 (0.5)
Age 70-79	184/8489	1.7 (0.2)	2.8 (0.2)	29/8494	0.2 (0.1)	0.5 (0.1)	341/8494	3.0 (0.2)	5.7 (0.3)	821/8488	6.7 (0.3)	14.6 (0.5)	82/3669	2.3 (0.3)	2.7 (0.3)
Age 80+	37/2808	1.4 (0.2)	2.1 (0.4)	5/2809	0.2 (0.1)	0.3 (0.2)	36/2809	1.4 (0.3)	2.1 (0.4)	231/2807	8.3 (0.6)	14.4 (1.2)	10/1026	1.3 (0.4)	1.3 (0.4)
**Gender**															
Male	175/7439	1.7 (0.2)	3.3 (0.3)	14/7444	0.2 (0.0)	0.3 (0.1)	355/7444	3.8 (0.2)	6.7 (0.4)	564/7443	5.5 (0.3)	11.7 (0.5)	---	---	---
Female	195/9893	1.5 (0.1)	2.6 (0.2)	40/9896	0.2 (0.0)	0.6 (0.1)	351/9896	2.5 (0.2)	5.0 (0.3)	1165/9887	8.0 (0.3)	16.8 (0.5)	---	---	---
**Race/Ethnicity**															
Black	153/6020	2.1 (0.2)	3.5 (0.3)	35/6024	0.3 (0.1)	0.9 (0.2)	166/6024	2.0 (0.2)	3.9 (0.3)	516/6019	5.6 (0.3)	13.3 (0.6)	58/2061	2.9 (0.4)	3.8 (0.5)
Non-Black	217/11312	1.4 (0.1)	2.5 (0.2)	19/11316	0.1 (0.0)	0.3 (0.1)	540/11316	3.6 (0.2)	6.7 (0.3)	1213/11311	7.6 (0.3)	15.5 (0.5)	152/5383	2.7 (0.2)	3.6 (0.3)
**Hispanic/Latino Ethnicity**															
Hispanic	41/3631	0.8 (0.2)	1.4 (0.2)	9/3631	0.1 (0.1)	0.4 (0.1)	121/3631	2.7 (0.3)	4.1 (0.4)	341/3630	7.6 (0.5)	11.8 (0.6)	45/1448	3.0 (0.5)	3.6 (0.5)
Non-Hispanic	327/13612	1.9 (0.1)	3.3 (0.2)	45/13620	0.2 (0.0)	0.5 (0.1)	582/13620	3.2 (0.2)	6.2 (0.3)	1380/13611	6.8 (0.2)	15.7 (0.4)	165/5948	2.7 (0.2)	3.7 (0.3)
**Education level**															
High school or less	261/11875	1.7 (0.1)	3.0 (0.2)	40/11880	0.2 (0.0)	0.5 (0.1)	409/11880	2.6 (0.2)	4.9 (0.3)	1138/11873	6.9 (0.2)	14.4 (0.4)	117/4631	2.5 (0.2)	3.4 (0.4)
More than high school	92/4151	1.6 (0.2)	2.9 (0.3)	12/4154	0.1 (0.1)	0.4 (0.1)	251/4154	4.4 (0.3)	8.1 (0.5)	470/4153	7.0 (0.4)	16.0 (0.7)	79/2286	3.2 (0.4)	4.1 (0.5)
**Treatment with antihypertensive drugs prior to trial baseline**															
Treated	342/15709	1.6 (0.1)	2.9 (0.2)	51/15717	0.2 (0.0)	0.5 (0.1)	652/15717	3.1 (0.1)	5.8 (0.2)	1584/15707	7.0 (0.2)	14.9 (0.4)	197/6629	2.9 (0.2)	3.8 (0.3)
Untreated	28/1623	1.4 (0.3)	2.3 (0.4)	3/1623	0.1 (0.1)	0.3 (0.2)	54/1623	2.3 (0.4)	4.6 (0.6)	145/1623	5.8 (0.6)	13.6 (1.1)	13/815	1.5 (0.4)	1.9 (0.5)
**Aspirin use (as of 1/1/1999)**															
Yes	144/6406	1.7 (0.2)	3.1 (0.3)	22/6410	0.2 (0.1)	0.5 (0.1)	273/6410	3.3 (0.2)	6.1 (0.4)	640/6406	6.7 (0.3)	15.5 (0.6)	109/3356	3.1 (0.3)	4.2 (0.4)
No	223/10760	1.6 (0.1)	2.8 (0.2)	32/10764	0.2 (0.0)	0.5 (0.1)	429/10764	2.9 (0.2)	5.5 (0.3)	1074/10758	7.1 (0.3)	14.4 (0.4)	99/4013	2.4 (0.3)	3.2 (0.3)
**Women taking estrogen at trial baseline**															
Yes	31/1444	1.6 (0.3)	2.6 (0.5)	8/1444	0.1 (0.1)	0.9 (0.3)	89/1444	3.7 (0.5)	7.9 (0.8)	229/1443	9.7 (0.8)	20.9 (1.3)	---	---	---
No	162/8271	1.5 (0.1)	2.6 (0.2)	32/8274	0.2 (0.1)	0.6 (0.1)	256/8274	2.3 (0.2)	4.4 (0.3)	918/8266	7.6 (0.3)	16.0 (0.5)	---	---	---
**HDL cholesterol <35 mg/dl (as of 1/1/1999)**															
Yes	53/2784	1.5 (0.3)	2.7 (0.4)	6/2784	0.1 (0.1)	0.3 (0.1)	136/2784	3.8 (0.4)	6.9 (0.6)	252/2784	6.4 (0.5)	13.7 (0.9)	52/1909	2.8 (0.4)	3.3 (0.5)
No	317/14548	1.7 (0.1)	2.9 (0.2)	48/14556	0.2 (0.0)	0.5 (0.1)	570/14556	2.9 (0.1)	5.5 (0.2)	1477/14546	7.0 (0.2)	14.9 (0.4)	158/5535	2.7 (0.2)	3.7 (0.3)
**Cigarette smoking at trial baseline**															
Never smoker	133/7649	1.3 (0.1)	2.3 (0.2)	25/7649	0.1 (0.0)	0.5 (0.1)	303/7649	2.8 (0.2)	5.3 (0.3)	831/7642	7.5 (0.3)	15.2 (0.5)	51/1895	2.5 (0.4)	3.3 (0.5)
Current smoker	86/2842	2.6 (0.3)	4.2 (0.5)	10/2845	0.3 (0.1)	0.5 (0.2)	91/2845	2.5 (0.3)	4.9 (0.6)	257/2843	6.4 (0.5)	14.6 (1.0)	31/1389	2.3 (0.4)	3.0 (0.6)
Former Smoker	151/6840	1.6 (0.2)	3.1 (0.3)	19/6845	0.2 (0.1)	0.4 (0.1)	312/6845	3.6 (0.2)	6.4 (0.4)	641/6844	6.5 (0.3)	14.2 (0.6)	128/4159	3.0 (0.3)	4.0 (0.4)
**Diabetes classification (as of 1/1/1999)**															
Diabetes	146/7091	1.6 (0.2)	3.0 (0.3)	23/7091	0.2 (0.1)	0.5 (0.1)	256/7091	2.9 (0.2)	5.2 (0.3)	725/7086	7.3 (0.3)	16.4 (0.6)	82/2889	2.8 (0.3)	3.6 (0.4)
Non-diabetes	195/8976	1.6 (0.1)	2.8 (0.2)	29/8981	0.2 (0.0)	0.5 (0.1)	410/8981	3.3 (0.2)	6.2 (0.3)	883/8976	6.6 (0.3)	13.8 (0.5)	110/4031	2.6 (0.3)	3.4 (0.3)
**History of Coronary Heart Disease (CHD) (as of 1/1/1999)**															
Yes	103/4845	1.7 (0.2)	2.9 (0.3)	15/4848	0.2 (0.1)	0.4 (0.1)	219/4848	3.6 (0.3)	7.0 (0.5)	469/4844	7.4 (0.4)	15.1 (0.7)	73/2593	2.8 (0.3)	3.9 (0.6)
No	267/12487	1.6 (0.1)	2.9 (0.2)	39/12492	0.2 (0.0)	0.5 (0.1)	487/12492	2.9 (0.2)	5.3 (0.2)	1260/12486	6.7 (0.2)	14.6 (0.4)	137/4851	2.7 (0.2)	3.5 (0.3)
**Atherosclerotic Cardiovascular Disease (ASCVD) at trial baseline**															
Yes	199/9479	1.6 (0.1)	2.9 (0.2)	31/9482	0.2 (0.0)	0.5 (0.1)	399/9482	3.2 (0.2)	6.2 (0.3)	962/9478	7.5 (0.3)	15.0 (0.5)	109/4351	2.5 (0.3)	3.3 (0.4)
No	171/7853	1.6 (0.2)	2.9 (0.2)	23/7858	0.2 (0.0)	0.5 (0.1)	307/7858	2.9 (0.2)	5.2 (0.3)	767/7852	6.3 (0.3)	14.4 (0.5)	101/3093	3.0 (0.3)	4.1 (0.4)
**History of myocardial infarction (MI) or stroke (as of 1/1/1999)**															

Yes	102/4702	1.9 (0.2)	3.2 (0.3)	11/4705	0.1 (0.1)	0.4 (0.1)	183/4705	3.2 (0.3)	6.3 (0.5)	503/4700	8.4 (0.4)	17.2 (0.8)	60/2388	2.7 (0.4)	3.3 (0.5)
No	268/12630	1.5 (0.1)	2.8 (0.2)	43/12635	0.2 (0.0)	0.5 (0.1)	523/12635	3.0 (0.2)	5.6 (0.3)	1226/12630	6.4 (0.2)	13.9 (0.4)	150/5056	2.8 (0.2)	3.7 (0.3)
**History of coronary artery bypass graft (CABG) (as of 1/1/1999)**															
Yes	45/2676	1.4 (0.2)	2.3 (0.4)	9/2677	0.3 (0.1)	0.4 (0.1)	129/2677	3.9 (0.4)	7.2 (0.7)	245/2676	6.8 (0.5)	14.7 (1.0)	57/1721	3.1 (0.4)	4.8 (0.8)
No	325/14656	1.7 (0.1)	3.0 (0.2)	45/14663	0.2 (0.0)	0.5 (0.1)	577/14663	2.9 (0.1)	5.5 (0.2)	1484/14654	6.9 (0.2)	14.8 (0.4)	153/5723	2.6 (0.2)	3.3 (0.3)
**Other ASCVD at trial baseline**															
Yes	91/4565	1.4 (0.2)	2.8 (0.3)	16/4567	0.2 (0.1)	0.6 (0.2)	185/4567	2.9 (0.3)	6.0 (0.5)	447/4565	7.4 (0.4)	14.2 (0.7)	40/1934	2.1 (0.3)	2.6 (0.4)
No	279/12767	1.7 (0.1)	2.9 (0.2)	38/12773	0.2 (0.0)	0.4 (0.1)	521/12773	3.1 (0.2)	5.6 (0.3)	1282/12765	6.8 (0.2)	14.9 (0.4)	170/5510	3.0 (0.2)	3.9 (0.3)
**Major ST segment depression (as of 1/1/1999)**															
Yes	30/1389	1.6 (0.4)	3.0 (0.6)	4/1389	0.1 (0.1)	0.6 (0.3)	68/1389	3.5 (0.5)	7.4 (1.0)	134/1389	6.9 (0.7)	14.9 (1.3)	19/554	3.6 (0.9)	4.1 (1.0)
No	339/15890	1.6 (0.1)	2.9 (0.2)	50/15898	0.2 (0.0)	0.5 (0.1)	637/15898	3.0 (0.1)	5.6 (0.2)	1589/15888	6.9 (0.2)	14.7 (0.4)	191/6872	2.7 (0.2)	3.6 (0.3)
**Left ventricular hypertrophy (LVH) by Minnesota code (as of 1/1/1999)**															
Hard LVH	11/675	1.6 (0.5)	2.2 (0.7)	1/675	0.2 (0.2)	0.2 (0.2)	22/675	3.3 (0.8)	4.8 (1.1)	63/674	6.9 (1.1)	18.7 (2.8)	8/266	3.3 (1.2)	4.9 (2.0)
No/Soft LVH	320/14767	1.6 (0.1)	2.9 (0.2)	50/14774	0.2 (0.0)	0.5 (0.1)	634/14774	3.2 (0.2)	6.0 (0.2)	1485/14767	6.9 (0.2)	14.7 (0.4)	182/6471	2.7 (0.2)	3.6 (0.3)
**Lipid Lowering Trial (LLT) participant**															
Yes	85/4217	1.5 (0.2)	2.7 (0.3)	10/4219	0.1 (0.1)	0.4 (0.1)	147/4219	2.8 (0.3)	4.6 (0.4)	403/4218	6.3 (0.4)	13.6 (0.7)	41/1757	2.2 (0.4)	2.9 (0.5)
No	285/13115	1.7 (0.1)	2.9 (0.2)	44/13121	0.2 (0.0)	0.5 (0.1)	559/13121	3.1 (0.2)	6.1 (0.3)	1326/13112	7.1 (0.2)	15.1 (0.4)	169/5687	2.9 (0.2)	3.8 (0.3)
**Obesity (BMI ≥ 30 kg/m²) at trial baseline**															
Yes	159/6875	1.7 (0.2)	3.1 (0.3)	26/6881	0.2 (0.1)	0.6 (0.1)	344/6881	3.7 (0.2)	6.7 (0.4)	785/6876	7.3 (0.3)	17.0 (0.6)	78/2570	3.1 (0.4)	3.6 (0.4)
No	211/10457	1.6 (0.1)	2.7 (0.2)	28/10459	0.2 (0.0)	0.4 (0.1)	362/10459	2.6 (0.2)	5.0 (0.3)	944/10454	6.7 (0.3)	13.2 (0.4)	132/4874	2.6 (0.2)	3.6 (0.3)

n= number of patients experiencing event; N= number of patients eligible to be studied for event; SE= Standard Error

Cancer diagnoses excluded non-melanoma skin cancers, and included patients from inpatient data only.

†18-year time to event includes events occurring up to, but not including year 19 (e.g. throughout the year 2017)

Table S3e: Cumulative Incidence (%) of 5 outcomes from primary diagnosis in Medicare Inpatient, Outpatient and Physician Carries claims on ≥2 times with 30 days apart by the 3 study drugs and other factors (1999 to 2017)

	Cancer			Angioedema			Insomnia			Depression			Erectile Dysfunction		
Demographic	Events/ Total (n/N)	6-Year % (SE)	18-Year % (SE)	Events/ Total (n/N)	6-Year % (SE)	18-Year % (SE)	Events/ Total (n/N)	6-Year % (SE)	18-Year % (SE)	Events/ Total n/N	6-Year % (SE)	18-Year % (SE)	Events/ Total (n/N)	6-Year % (SE)	18-Year % (SE)
All Patients	4999/17329	24.1 (0.3)	36.2 (0.4)	54/17340	0.2 (0)	0.5 (0.1)	706/17340	3.1 (0.1)	5.7 (0.2)	1729/17330	6.9 (0.2)	14.7 (0.4)	218/7444	2.8 (0.2)	3.7 (0.3)
**Randomized group**															
Chlorthalidone	2269/7992	24.0 (0.5)	36.0 (0.7)	19/7996	0.1 (0.0)	0.4 (0.1)	320/7996	3.0 (0.2)	5.5 (0.3)	796/7992	6.9 (0.3)	14.5 (0.5)	99/3425	2.7 (0.3)	3.8 (0.4)
Amlodipine	1336/4678	24.1 (0.7)	35.0 (0.8)	15/4682	0.2 (0.1)	0.5 (0.1)	205/4682	3.3 (0.3)	6.2 (0.5)	455/4680	6.7 (0.4)	14.5 (0.7)	57/1969	2.9 (0.4)	3.7 (0.5)
Lisinopril	1394/4659	24.4 (0.7)	37.7 (0.9)	20/4662	0.2 (0.1)	0.7 (0.2)	181/4662	2.9 (0.3)	5.6 (0.4)	478/4658	7.2 (0.4)	15.3 (0.7)	62/2050	2.9 (0.4)	3.7 (0.5)
**Age group (as of 1/1/1999)**															
Age <70	1833/6035	22.4 (0.6)	36.0 (0.7)	20/6037	0.1 (0.0)	0.5 (0.1)	329/6037	3.7 (0.3)	6.7 (0.4)	677/6035	6.7 (0.3)	14.7 (0.6)	122/2749	3.9 (0.4)	5.3 (0.5)
Age 70-79	2551/8487	25.5 (0.5)	37.0 (0.6)	29/8494	0.2 (0.1)	0.5 (0.1)	341/8494	3.0 (0.2)	5.7 (0.3)	821/8488	6.7 (0.3)	14.6 (0.5)	85/3669	2.3 (0.3)	2.8 (0.3)
Age 80+	615/2807	23.3 (0.9)	29.6 (1.2)	5/2809	0.2 (0.1)	0.3 (0.2)	36/2809	1.4 (0.3)	2.1 (0.4)	231/2807	8.3 (0.6)	14.4 (1.2)	11/1026	1.3 (0.4)	1.6 (0.5)
**Gender**															
Male	2704/7437	32.2 (0.6)	46.0 (0.7)	14/7444	0.2 (0.0)	0.3 (0.1)	355/7444	3.8 (0.2)	6.7 (0.4)	564/7443	5.5 (0.3)	11.7 (0.5)	---	---	---
Female	2295/9892	18.2 (0.4)	29.3 (0.6)	40/9896	0.2 (0.0)	0.6 (0.1)	351/9896	2.5 (0.2)	5.0 (0.3)	1165/9887	8.0 (0.3)	16.8 (0.5)	---	---	---
**Race/Ethnicity**															
Black	1445/6019	19.4 (0.5)	30.9 (0.7)	35/6024	0.3 (0.1)	0.9 (0.2)	166/6024	2.0 (0.2)	3.9 (0.3)	516/6019	5.6 (0.3)	13.3 (0.6)	61/2061	2.9 (0.4)	4.0 (0.5)
Non-Black	3554/11310	26.6 (0.4)	39.0 (0.6)	19/11316	0.1 (0.0)	0.3 (0.1)	540/11316	3.6 (0.2)	6.7 (0.3)	1213/11311	7.6 (0.3)	15.5 (0.5)	157/5383	2.8 (0.2)	3.7 (0.3)
**Hispanic/Latino Ethnicity**															
Hispanic	764/3631	19.0 (0.7)	24.4 (0.8)	9/3631	0.1 (0.1)	0.4 (0.1)	121/3631	2.7 (0.3)	4.1 (0.4)	341/3630	7.6 (0.5)	11.8 (0.6)	49/1448	3.2 (0.5)	3.9 (0.6)
Non-Hispanic	4204/13609	25.5 (0.4)	39.6 (0.5)	45/13620	0.2 (0.0)	0.5 (0.1)	582/13620	3.2 (0.2)	6.2 (0.3)	1380/13611	6.8 (0.2)	15.7 (0.4)	169/5948	2.7 (0.2)	3.8 (0.3)
**Education level**															
High school or less	3169/11875	22.3 (0.4)	34.0 (0.5)	40/11880	0.2 (0.0)	0.5 (0.1)	409/11880	2.6 (0.2)	4.9 (0.3)	1138/11873	6.9 (0.2)	14.4 (0.4)	122/4631	2.5 (0.2)	3.6 (0.4)
More than high school	1511/4150	30.1 (0.7)	43.7 (0.9)	12/4154	0.1 (0.1)	0.4 (0.1)	251/4154	4.4 (0.3)	8.1 (0.5)	470/4153	7.0 (0.4)	16.0 (0.7)	82/2286	3.4 (0.4)	4.2 (0.5)
**Treatment with antihypertensive drugs prior to trial baseline**															
Treated	4554/15707	24.3 (0.4)	36.4 (0.5)	51/15717	0.2 (0.0)	0.5 (0.1)	652/15717	3.1 (0.1)	5.8 (0.2)	1584/15707	7.0 (0.2)	14.9 (0.4)	203/6629	2.9 (0.2)	3.9 (0.3)
Untreated	445/1622	22.3 (1.1)	34.1 (1.4)	3/1623	0.1 (0.1)	0.3 (0.2)	54/1623	2.3 (0.4)	4.6 (0.6)	145/1623	5.8 (0.6)	13.6 (1.1)	15/815	1.8 (0.5)	2.2 (0.6)
**Aspirin use (as of 1/1/1999)**															
Yes	2000/6404	26.2 (0.6)	39.3 (0.8)	22/6410	0.2 (0.1)	0.5 (0.1)	273/6410	3.3 (0.2)	6.1 (0.4)	640/6406	6.7 (0.3)	15.5 (0.6)	112/3356	3.2 (0.3)	4.3 (0.4)
No	2966/10759	23.0 (0.4)	34.5 (0.6)	32/10764	0.2 (0.0)	0.5 (0.1)	429/10764	2.9 (0.2)	5.5 (0.3)	1074/10758	7.1 (0.3)	14.4 (0.4)	104/4013	2.5 (0.3)	3.3 (0.3)
**Women taking estrogen at trial baseline**															
Yes	423/1444	21.5 (1.1)	34.6 (1.4)	8/1444	0.1 (0.1)	0.9 (0.3)	89/1444	3.7 (0.5)	7.9 (0.8)	229/1443	9.7 (0.8)	20.9 (1.3)	---	---	---
No	1841/8270	17.7 (0.4)	28.4 (0.6)	32/8274	0.2 (0.1)	0.6 (0.1)	256/8274	2.3 (0.2)	4.4 (0.3)	918/8266	7.6 (0.3)	16.0 (0.5)	---	---	---
**HDL cholesterol <35 mg/dl (as of 1/1/1999)**															
Yes	951/2784	29.9 (0.9)	43.3 (1.2)	6/2784	0.1 (0.1)	0.3 (0.1)	136/2784	3.8 (0.4)	6.9 (0.6)	252/2784	6.4 (0.5)	13.7 (0.9)	53/1909	2.8 (0.4)	3.3 (0.5)
No	4048/14545	23.0 (0.4)	34.9 (0.5)	48/14556	0.2 (0.0)	0.5 (0.1)	570/14556	2.9 (0.1)	5.5 (0.2)	1477/14546	7.0 (0.2)	14.9 (0.4)	165/5535	2.8 (0.2)	3.9 (0.3)
**Cigarette smoking at trial baseline**															
Never smoker	1963/7649	20.9 (0.5)	31.5 (0.6)	25/7649	0.1 (0.0)	0.5 (0.1)	303/7649	2.8 (0.2)	5.3 (0.3)	831/7642	7.5 (0.3)	15.2 (0.5)	54/1895	2.6 (0.4)	3.5 (0.5)
Current smoker	834/2841	25.1 (0.9)	38.7 (1.2)	10/2845	0.3 (0.1)	0.5 (0.2)	91/2845	2.5 (0.3)	4.9 (0.6)	257/2843	6.4 (0.5)	14.6 (1.0)	32/1389	2.4 (0.4)	3.1 (0.6)
Former Smoker	2202/6838	27.4 (0.6)	40.9 (0.8)	19/6845	0.2 (0.1)	0.4 (0.1)	312/6845	3.6 (0.2)	6.4 (0.4)	641/6844	6.5 (0.3)	14.2 (0.6)	132/4159	3.0 (0.3)	4.1 (0.4)
**Diabetes classification (as of 1/1/1999)**															
Diabetes	1834/7091	22.3 (0.5)	34.0 (0.7)	23/7091	0.2 (0.1)	0.5 (0.1)	256/7091	2.9 (0.2)	5.2 (0.3)	725/7086	7.3 (0.3)	16.4 (0.6)	84/2889	2.8 (0.3)	3.7 (0.4)
Non-diabetes	2800/8974	25.6 (0.5)	37.8 (0.6)	29/8981	0.2 (0.0)	0.5 (0.1)	410/8981	3.3 (0.2)	6.2 (0.3)	883/8976	6.6 (0.3)	13.8 (0.5)	116/4031	2.7 (0.3)	3.6 (0.4)
**History of Coronary Heart Disease (CHD) (as of 1/1/1999)**															
Yes	1462/4845	26.3 (0.7)	38.8 (0.9)	15/4848	0.2 (0.1)	0.4 (0.1)	219/4848	3.6 (0.3)	7.0 (0.5)	469/4844	7.4 (0.4)	15.1 (0.7)	74/2593	2.8 (0.3)	4.0 (0.6)
No	3537/12484	23.3 (0.4)	35.3 (0.5)	39/12492	0.2 (0.0)	0.5 (0.1)	487/12492	2.9 (0.2)	5.3 (0.2)	1260/12486	6.7 (0.2)	14.6 (0.4)	144/4851	2.8 (0.3)	3.7 (0.3)
**Atherosclerotic Cardiovascular Disease (ASCVD) at trial baseline**															
Yes	2757/9477	24.8 (0.5)	37.0 (0.6)	31/9482	0.2 (0.0)	0.5 (0.1)	399/9482	3.2 (0.2)	6.2 (0.3)	962/9478	7.5 (0.3)	15.0 (0.5)	113/4351	2.5 (0.3)	3.4 (0.4)
No	2242/7852	23.3 (0.5)	35.3 (0.6)	23/7858	0.2 (0.0)	0.5 (0.1)	307/7858	2.9 (0.2)	5.2 (0.3)	767/7852	6.3 (0.3)	14.4 (0.5)	105/3093	3.2 (0.3)	4.2 (0.4)
**History of myocardial infarction (MI) or stroke (as of 1/1/1999)**															

Yes	1311/4701	25.1 (0.7)	38.0 (1.0)	11/4705	0.1 (0.1)	0.4 (0.1)	183/4705	3.2 (0.3)	6.3 (0.5)	503/4700	8.4 (0.4)	17.2 (0.8)	61/2388	2.7 (0.4)	3.4 (0.5)
No	3688/12628	23.8 (0.4)	35.7 (0.5)	43/12635	0.2 (0.0)	0.5 (0.1)	523/12635	3.0 (0.2)	5.6 (0.3)	1226/12630	6.4 (0.2)	13.9 (0.4)	157/5056	2.9 (0.2)	3.9 (0.3)
**History of coronary artery bypass graft (CABG) (as of 1/1/1999)**															
Yes	862/2676	27.8 (0.9)	42.1 (1.3)	9/2677	0.3 (0.1)	0.4 (0.1)	129/2677	3.9 (0.4)	7.2 (0.7)	245/2676	6.8 (0.5)	14.7 (1.0)	57/1721	3.1 (0.4)	4.8 (0.8)
No	4137/14653	23.5 (0.4)	35.2 (0.5)	45/14663	0.2 (0.0)	0.5 (0.1)	577/14663	2.9 (0.1)	5.5 (0.2)	1484/14654	6.9 (0.2)	14.8 (0.4)	161/5723	2.7 (0.2)	3.5 (0.3)
**Other ASCVD at trial baseline**															
Yes	1342/4564	25.0 (0.7)	36.9 (0.9)	16/4567	0.2 (0.1)	0.6 (0.2)	185/4567	2.9 (0.3)	6.0 (0.5)	447/4565	7.4 (0.4)	14.2 (0.7)	43/1934	2.1 (0.3)	2.9 (0.5)
No	3657/12765	23.8 (0.4)	36.0 (0.5)	38/12773	0.2 (0.0)	0.4 (0.1)	521/12773	3.1 (0.2)	5.6 (0.3)	1282/12765	6.8 (0.2)	14.9 (0.4)	175/5510	3.1 (0.2)	4.0 (0.3)
**Major ST segment depression (as of 1/1/1999)**															
Yes	376/1389	23.5 (1.2)	35.4 (1.6)	4/1389	0.1 (0.1)	0.6 (0.3)	68/1389	3.5 (0.5)	7.4 (1.0)	134/1389	6.9 (0.7)	14.9 (1.3)	20/554	3.6 (0.9)	4.4 (1.0)
No	4611/15887	24.2 (0.4)	36.3 (0.5)	50/15898	0.2 (0.0)	0.5 (0.1)	637/15898	3.0 (0.1)	5.6 (0.2)	1589/15888	6.9 (0.2)	14.7 (0.4)	198/6872	2.7 (0.2)	3.7 (0.3)
**Left ventricular hypertrophy (LVH) by Minnesota code (as of 1/1/1999)**															
Hard LVH	141/675	20.3 (1.7)	27.3 (2.2)	1/675	0.2 (0.2)	0.2 (0.2)	22/675	3.3 (0.8)	4.8 (1.1)	63/674	6.9 (1.1)	18.7 (2.8)	8/266	3.3 (1.2)	4.9 (2.0)
No/Soft LVH	4369/14765	24.4 (0.4)	37.2 (0.5)	50/14774	0.2 (0.0)	0.5 (0.1)	634/14774	3.2 (0.2)	6.0 (0.2)	1485/14767	6.9 (0.2)	14.7 (0.4)	190/6471	2.8 (0.2)	3.8 (0.3)
**Lipid Lowering Trial (LLT) participant**															
Yes	1207/4217	23.8 (0.7)	35.2 (0.9)	10/4219	0.1 (0.1)	0.4 (0.1)	147/4219	2.8 (0.3)	4.6 (0.4)	403/4218	6.3 (0.4)	13.6 (0.7)	44/1757	2.3 (0.4)	3.1 (0.5)
No	3792/13112	24.2 (0.4)	36.6 (0.5)	44/13121	0.2 (0.0)	0.5 (0.1)	559/13121	3.1 (0.2)	6.1 (0.3)	1326/13112	7.1 (0.2)	15.1 (0.4)	174/5687	3.0 (0.2)	3.9 (0.3)
**Obesity (BMI ≥ 30 kg/m²) at trial baseline**															
Yes	1967/6874	22.8 (0.5)	35.9 (0.7)	26/6881	0.2 (0.1)	0.6 (0.1)	344/6881	3.7 (0.2)	6.7 (0.4)	785/6876	7.3 (0.3)	17.0 (0.6)	80/2570	3.1 (0.4)	3.8 (0.4)
No	3032/10455	25.0 (0.4)	36.4 (0.6)	28/10459	0.2 (0.0)	0.4 (0.1)	362/10459	2.6 (0.2)	5.0 (0.3)	944/10454	6.7 (0.3)	13.2 (0.4)	138/4874	2.7 (0.2)	3.7 (0.3)

n= number of patients experiencing event; N= number of patients eligible to be studied for event; SE= Standard Error

Cancer diagnoses excluded non-melanoma skin cancers, and included patients from inpatient, outpatient, and carrier data.

†18-year time to event includes events occurring up to, but not including year 19 (e.g. throughout the year 2017)

Table S4: Adjusted* Hazard Ratios (HR) of having 5 outcomes from any diagnosis in Medicare Inpatient, Outpatient and Physician Carries claims on ≥1 times by the 3 study drugs and other factors (1999 to 2017) calculated using Standard Cox Proportional Hazard Modeling

Demographic	Cancer				Angioedema				Insomnia				Depression				Erectile Dysfunction (in men)			
	6-year		18-year		6-year		18-year		6-year		18-year		6-year		18-year		6-year		18-year	
	HR (95% CI)	P-value	HR (95% CI)	P-value	HR (95% CI)	P-value	HR (95% CI)	P-value	HR (95% CI)	P-value	HR (95% CI)	P-value	HR (95% CI)	P-value	HR (95% CI)	P-value	HR (95% CI)	P-value	HR (95% CI)	P-value
**Randomized group**																				
Chlorthalidone vs Amlodipine	1.06 (0.99, 1.14)	0.079	1.01 (0.95, 1.07)	0.862	0.82 (0.54, 1.45)	0.632	0.82 (0.58, 1.15)	0.247	1.18 (1.04, 1.34)	**0.008**	1.07 (0.97, 1.17)	0.160	1.08 (0.98, 1.19)	0.105	1.02 (0.95, 1.10)	0.526	1.05 (0.87, 1.27)	0.621	0.91 (0.76, 1.08)	0.265
Lisinopril vs Amlodipine	1.00 (0.92, 1.08)	0.923	1.03 (0.96, 1.10)	0.384	1.63 (1.17, 3.20)	**0.010**	1.63 (1.14, 2.33)	**0.007**	0.88 (0.77, 1.02)	0.092	0.90 (0.81, 1.00)	**0.046**	0.97 (0.87, 1.08)	0.544	0.95 (0.87, 1.03)	0.188	1.01 (0.82, 1.25)	0.932	1.09 (0.90, 1.33)	0.360
Lisinopril vs Chlorthalidone‡	1.06 (0.99, 1.13)	0.099	1.03 (0.98, 1.10)	0.247	1.33 (1.13, 2.60)	**0.011**	1.33 (1.00, 1.79)	0.053	1.04 (0.92, 1.19)	0.505	0.96 (0.87, 1.05)	0.398	1.05 (0.95, 1.15)	0.361	0.97 (0.90, 1.04)	0.398	1.06 (0.88, 1.27)	0.545	0.99 (0.84, 1.17)	0.924
**Age group (as of 1/1/1999)**																				
Age <70	1.00 (ref)		1.00 (ref)		1.00 (ref)		1.00 (ref)		1.00 (ref)		1.00 (ref)		1.00 (ref)		1.00 (ref)		1.00 (ref)		1.00 (ref)	
Age 70-79	1.01 (0.94, 1.07)	0.866	1.21 (1.15, 1.27)	**<0.001**	1.03 (0.50, 1.13)	0.167	1.03 (0.78, 1.35)	0.853	0.74 (0.66, 0.83)	**<0.001**	0.96 (0.89, 1.04)	0.366	0.81 (0.74, 0.89)	**<0.001**	1.10 (1.03, 1.18)	**0.004**	0.57 (0.49, 0.67)	**<0.001**	0.70 (0.60, 0.81)	**<0.001**
Age 80+	0.76 (0.69, 0.83)	**<0.001**	1.38 (1.28, 1.50)	**<0.001**	1.10 (0.22, 0.71)	**0.002**	1.10 (0.70, 1.74)	0.669	0.38 (0.32, 0.46)	**<0.001**	0.90 (0.78, 1.03)	0.135	0.64 (0.57, 0.73)	**<0.001**	1.47 (1.34, 1.62)	**<0.001**	0.20 (0.14, 0.27)	**<0.001**	0.38 (0.27, 0.52)	**<0.001**
**Gender**																				
Female† vs Male	0.78 (0.73, 0.83)	**<0.001**	0.67 (0.63, 0.70)	**<0.001**	1.32 (0.90, 2.06)	0.148	1.32 (0.97, 1.80)	0.073	1.25 (1.11, 1.41)	**<0.001**	1.14 (1.05, 1.25)	**0.003**	1.52 (1.39, 1.67)	**<0.001**	1.57 (1.46, 1.69)	**<0.001**	---	---	---	---
**Race/Ethnicity**																				
Black vs Non-Black	0.91 (0.85, 0.97)	**0.006**	0.78 (0.74, 0.83)	**<0.001**	2.64 (1.25, 2.85)	**0.002**	2.64 (1.98, 3.54)	**<0.001**	0.66 (0.58, 0.75)	**<0.001**	0.64 (0.58, 0.70)	**<0.001**	0.73 (0.66, 0.80)	**<0.001**	0.68 (0.64, 0.74)	**<0.001**	0.99 (0.82, 1.20)	0.922	1.23 (1.04, 1.47)	**0.018**
**Hispanic/Latino Ethnicity**																				
Hispanic vs Non-Hispanic	1.02 (0.95, 1.11)	0.534	0.78 (0.73, 0.83)	**<0.001**	0.68 (0.55, 1.71)	0.903	0.68 (0.44, 1.05)	0.086	1.05 (0.91, 1.21)	0.494	0.69 (0.62, 0.77)	**<0.001**	0.93 (0.83, 1.04)	0.200	0.62 (0.57, 0.68)	**<0.001**	1.01 (0.81, 1.25)	0.959	0.96 (0.78, 1.17)	0.668
**Education level**																				
Less than high school vs more	0.90 (0.84, 0.96)	**<0.001**	0.94 (0.89, 0.99)	**0.018**	0.83 (0.50, 1.18)	0.223	0.83 (0.62, 1.12)	0.221	0.83 (0.74, 0.93)	**0.002**	0.97 (0.89, 1.05)	0.432	0.90 (0.82, 0.99)	**0.030**	1.03 (0.96, 1.10)	0.478	0.81 (0.69, 0.96)	**0.013**	0.96 (0.83, 1.12)	0.604
**Treatment with antihypertensive drugs prior to trial baseline**																				
Treated vs Untreated	0.98 (0.88, 1.08)	0.624	1.10 (1.01, 1.20)	**0.027**	1.44 (0.47, 1.79)	0.806	1.44 (0.83, 2.48)	0.193	1.18 (0.96, 1.44)	0.109	1.18 (1.02, 1.36)	**0.023**	1.10 (0.95, 1.28)	0.203	1.09 (0.98, 1.21)	0.124	1.15 (0.88, 1.50)	0.313	1.18 (0.93, 1.51)	0.166
**Aspirin use (as of 1/1/1999)**																				
Yes vs No	1.00 (0.94, 1.07)	0.939	0.95 (0.90, 1.01)	0.086	1.05 (0.85, 1.87)	0.249	1.05 (0.78, 1.40)	0.762	1.07 (0.96, 1.20)	0.244	0.95 (0.87, 1.03)	0.216	0.90 (0.82, 0.98)	**0.017**	0.94 (0.88, 1.01)	0.089	1.25 (1.06, 1.47)	**0.007**	1.08 (0.93, 1.26)	0.333
**HDL cholesterol <35 mg/dl (as of 1/1/1999)**																				
Yes vs No	0.91 (0.84, 0.98)	**0.010**	1.04 (0.97, 1.11)	0.299	1.00 (0.40, 1.22)	0.207	1.00 (0.66, 1.52)	0.984	0.75 (0.65, 0.87)	**<0.001**	0.96 (0.86, 1.07)	0.447	0.86 (0.77, 0.96)	**0.008**	1.03 (0.94, 1.12)	0.507	1.03 (0.86, 1.23)	0.752	1.04 (0.89, 1.23)	0.610
**Cigarette smoking at trial baseline**																				
Never smoker	1.00		1.00		1.00		1.00		1.00		1.00		1.00		1.00		1.00		1.00	
Current smoker	0.91 (0.83, 0.99)	**0.028**	1.19 (1.10, 1.28)	**<0.001**	1.25 (0.46, 1.38)	0.409	1.25 (0.84, 1.87)	0.276	0.58 (0.49, 0.69)	**<0.001**	1.04 (0.92, 1.18)	0.516	0.83 (0.73, 0.93)	**0.002**	1.27 (1.15, 1.39)	**<0.001**	0.46 (0.35, 0.59)	**<0.001**	0.73 (0.58, 0.91)	**0.006**
Former Smoker	0.98 (0.92, 1.05)	0.656	1.09 (1.03, 1.15)	**0.004**	1.15 (0.62, 1.49)	0.853	1.15 (0.86, 1.55)	0.344	0.98 (0.87, 1.10)	0.723	1.08 (0.99, 1.18)	0.079	0.94 (0.86, 1.03)	0.218	1.11 (1.04, 1.19)	**0.003**	0.81 (0.68, 0.98)	**0.028**	0.93 (0.79, 1.10)	0.414
**Diabetes classification (as of 1/1/1999)**																				
Diabetes vs Non-diabetes	0.83 (0.78, 0.89)	**<0.001**	0.99 (0.94, 1.04)	0.736	1.03 (0.45, 1.00)	0.051	1.03 (0.78, 1.36)	0.839	0.71 (0.64, 0.80)	**<0.001**	1.06 (0.98, 1.15)	0.161	0.90 (0.83, 0.99)	**0.021**	1.23 (1.15, 1.31)	**<0.001**	0.59 (0.50, 0.70)	**<0.001**	0.81 (0.69, 0.94)	**0.006**
**History of Coronary Heart Disease (CHD) (as of 1/1/1999)**																				
Yes vs No	1.07 (0.98, 1.16)	0.125	1.00 (0.93, 1.07)	1.000	0.81 (0.63, 1.81)	0.813	0.81 (0.55, 1.19)	0.286	1.03 (0.89, 1.20)	0.681	1.07 (0.95, 1.19)	0.259	1.00 (0.89, 1.12)	0.997	0.98 (0.90, 1.07)	0.691	0.95 (0.75, 1.19)	0.637	0.93 (0.76, 1.15)	0.528
**Atherosclerotic Cardiovascular Disease (ASCVD) at trial baseline**																				
Yes vs No	0.91 (0.83, 1.00)	0.060	1.03 (0.95, 1.11)	0.495	1.52 (0.60, 1.95)	0.790	1.52 (1.01, 2.30)	**0.045**	0.98 (0.83, 1.16)	0.796	1.06 (0.94, 1.20)	0.351	1.00 (0.88, 1.14)	0.985	1.01 (0.91, 1.11)	0.846	0.84 (0.66, 1.07)	0.156	1.04 (0.83, 1.30)	0.765
**History of myocardial infarction (MI) or stroke (as of 1/1/1999)**																				
Yes vs No	0.87 (0.80, 0.94)	**<0.001**	0.99 (0.93, 1.06)	0.842	0.99 (0.41, 1.11)	0.121	0.99 (0.68, 1.43)	0.939	0.88 (0.76, 1.01)	0.070	1.03 (0.92, 1.15)	0.609	1.09 (0.98, 1.21)	0.129	1.27 (1.17, 1.38)	**<0.001**	0.72 (0.59, 0.89)	**0.002**	0.84 (0.69, 1.02)	0.077

**History of coronary artery bypass graft (CABG) (as of 1/1/1999)**																				
Yes vs No	0.97 (0.88, 1.06)	0.473	1.01 (0.93, 1.09)	0.828	1.13 (0.63, 2.06)	0.658	1.13 (0.71, 1.79)	0.612	0.84 (0.71, 1.00)	**0.046**	1.07 (0.94, 1.21)	0.299	0.95 (0.84, 1.09)	0.482	1.06 (0.96, 1.18)	0.225	1.06 (0.83, 1.34)	0.645	1.07 (0.86, 1.33)	0.528
**Other ASCVD at trial baseline**																				
Yes vs No	1.06 (0.98, 1.16)	0.138	1.04 (0.97, 1.11)	0.316	0.90 (0.48, 1.40)	0.471	0.90 (0.61, 1.31)	0.576	1.04 (0.90, 1.21)	0.591	1.04 (0.93, 1.16)	0.520	1.00 (0.89, 1.12)	0.970	1.07 (0.98, 1.17)	0.122	0.85 (0.68, 1.05)	0.129	0.86 (0.70, 1.05)	0.137
**Major ST segment depression (as of 1/1/1999)**																				
Yes vs No	1.00 (0.89, 1.11)	0.932	1.07 (0.97, 1.18)	0.161	0.92 (0.43, 1.71)	0.659	0.92 (0.55, 1.55)	0.762	0.91 (0.74, 1.12)	0.361	1.07 (0.92, 1.25)	0.369	0.90 (0.77, 1.05)	0.177	0.97 (0.86, 1.10)	0.672	1.03 (0.77, 1.37)	0.864	1.27 (0.97, 1.65)	0.084
**Left ventricular hypertrophy (LVH) by Minnesota code (as of 1/1/1999)**																				
Hard LVH vs No/Soft LVH	0.87 (0.74, 1.02)	0.080	0.90 (0.78, 1.03)	0.123	0.67 (0.22, 1.66)	0.326	0.67 (0.31, 1.44)	0.301	0.78 (0.59, 1.05)	0.101	0.89 (0.71, 1.12)	0.322	0.93 (0.76, 1.13)	0.452	1.18 (1.01, 1.39)	**0.037**	0.99 (0.67, 1.48)	0.980	0.99 (0.68, 1.45)	0.976
**Lipid Lowering Trial (LLT) participant**																				
Yes vs No	1.03 (0.96, 1.10)	0.374	0.99 (0.94, 1.05)	0.803	0.86 (0.53, 1.30)	0.426	0.86 (0.63, 1.16)	0.316	1.05 (0.93, 1.18)	0.458	0.99 (0.91, 1.09)	0.907	1.03 (0.94, 1.13)	0.540	0.94 (0.88, 1.01)	0.094	0.98 (0.81, 1.17)	0.792	0.90 (0.76, 1.07)	0.224
**Obesity (BMI ≥ 30 kg/m^2) at trial baseline**																				
Yes vs No	1.00 (0.94, 1.07)	0.924	1.01 (0.96, 1.06)	0.684	1.40 (0.91, 1.96)	0.139	1.40 (1.07, 1.83)	**0.015**	1.22 (1.10, 1.37)	**<0.001**	1.24 (1.15, 1.34)	**<0.001**	1.06 (0.98, 1.15)	0.170	1.11 (1.04, 1.18)	**0.002**	0.99 (0.84, 1.17)	0.935	0.93 (0.80, 1.08)	0.327
**Blood pressure change from the trial baseline to the latest BP reading prior to 1/1/1999, per 10 mmHg**																				
Systolic BP	0.99 (0.97, 1.01)	0.446	1.00 (0.99, 1.02)	0.651	1.01 (0.92, 1.16)	0.593	1.01 (0.93, 1.10)	0.790	1.00 (0.97, 1.04)	0.933	1.01 (0.98, 1.04)	0.451	1.00 (0.97, 1.02)	0.949	1.00 (0.98, 1.03)	0.633	0.98 (0.93, 1.03)	0.445	1.01 (0.96, 1.06)	0.821
Diastolic BP	1.03 (0.99, 1.06)	0.113	1.02 (0.99, 1.05)	0.252	1.04 (0.83, 1.26)	0.817	1.04 (0.90, 1.21)	0.574	1.02 (0.96, 1.08)	0.602	1.00 (0.96, 1.05)	0.847	1.01 (0.96, 1.05)	0.759	1.00 (0.96, 1.03)	0.847	1.07 (0.98, 1.17)	0.131	1.02 (0.93, 1.11)	0.686

Cancer diagnoses excluded non-melanoma skin cancers, and included patients from inpatient, outpatient, and carrier data.
18-year time to event includes events occurring up to, but not including year 19 (e.g. throughout the year 2017)
HR= Hazard Ratio; CI= Confidence Interval
* Adjusted for each covariate shown, in addition to all others presented in the table.
†Estrogen use was evaluated in women only, which prevented simultaneous inclusion of sex and estrogen as covariates
‡Contrast estimates were garnered from the same model, using a different reference group for randomized group (Chlorthalidone or Amlodipine).
